# Sotagliflozin attenuates liver-associated disorders in cystic fibrosis rabbits

**DOI:** 10.1172/jci.insight.165826

**Published:** 2024-02-15

**Authors:** Xiubin Liang, Xia Hou, Mohamad Bouhamdan, Yifei Sun, Zhenfeng Song, Carthic Rajagopalan, Hong Jiang, Hong-Guang Wei, Jun Song, Dongshan Yang, Yanhong Guo, Yihan Zhang, Hongmei Mou, Jifeng Zhang, Y. Eugene Chen, Fei Sun, Jian-Ping Jin, Kezhong Zhang, Jie Xu

**Affiliations:** 1 Center for Advanced Models for Translational Sciences and Therapeutics, University of Michigan Medical Center, Ann Arbor, Michigan, USA.; 2 Department of Physiology, and; 3 Center for Molecular Medicine and Genetics, Wayne State University School of Medicine, Detroit, Michigan, USA.; 4 The Mucosal Immunology and Biology Research Center, Massachusetts General Hospital, Boston, Massachusetts, USA.

**Keywords:** Hepatology, Therapeutics, Genetic diseases

## Abstract

Mutations in the cystic fibrosis (CF) transmembrane conductance regulator (CFTR) gene lead to CF, a life-threating autosomal recessive genetic disease. While recently approved Trikafta dramatically ameliorates CF lung diseases, there is still a lack of effective medicine to treat CF-associated liver disease (CFLD). To address this medical need, we used a recently established CF rabbit model to test whether sotagliflozin, a sodium-glucose cotransporter 1 and 2 (SGLT1/2) inhibitor drug that is approved to treat diabetes, can be repurposed to treat CFLD. Sotagliflozin treatment led to systemic benefits to CF rabbits, evidenced by increased appetite and weight gain as well as prolonged lifespan. For CF liver-related phenotypes, the animals benefited from normalized blood chemistry and bile acid parameters. Furthermore, sotagliflozin alleviated nonalcoholic steatohepatitis–like phenotypes, including liver fibrosis. Intriguingly, sotagliflozin treatment markedly reduced the otherwise elevated endoplasmic reticulum stress responses in the liver and other affected organs of CF rabbits. In summary, our work demonstrates that sotagliflozin attenuates liver disorders in CF rabbits and suggests sotagliflozin as a potential drug to treat CFLD.

## Abstract 2

Sotagliflozin ameliorates cystic fibrosis-associated liver disease in a rabbit disease model.

## Introduction

Mutations in the cystic fibrosis (CF) transmembrane conductance regulator (CFTR) gene (1) lead to CF, the most common life-threatening autosomal recessive disease among people of European descent (2). Lung disease is the primary cause of CF morbidity and mortality. CF-associated liver disease (CFLD) is a common extrapulmonary manifestation in CF, affecting approximately one-third of patients (3). In 2019, the FDA approved Trikafta for treating CF patients who carry the most prevalent CFTR-F508del mutation (4). Although Trikafta greatly improves patients’ pulmonary functions, benefits for liver disease have yet to be verified, and emerging data suggest that it may add a burden to the liver conditions of CF patients (5–10). Given that the life expectancy of CF patients is expected to continuously improve in the post-Trikafta era, addressing liver disease is now a priority for CF research.

The lack of an effective medicine for CFLD is due in part to the lack of clinically relevant, yet laboratory friendly, animal models. We recently developed CF rabbits using CRISPR/Cas9 technology (11, 12). These animals manifest many typical CF features, including liver and metabolic disorders (11–14). Compared with other nonrodent models, such as pigs (15), ferrets (16), and sheep (17), CF rabbits are relatively cost effective to house and maintain. Compared with the mouse (18) and rat (19) models, CF rabbits are of large size, making many experimental procedures more practical. These features make the CF rabbit an attractive model system for the study of CF liver- and metabolically related disorders.

Sodium-glucose cotransporters 1 and 2 (SGLT1/2) belong to the family of glucose transporters, encoded by *SLC5A1* and *SLC5A2* (20), respectively. SGLT2 is almost exclusively expressed in the apical membrane of proximal convoluted renal tubule cells, a site that is minimally affected in CF. In contrast, SGLT1 is not only expressed in the kidney but also in many other CF-relevant organs such as the lung, the intestine, and the liver (21).

The past decade has witnessed the success of several SGLT inhibitor (SGLTi) drugs to treat diabetes. Examples include canagliflozin, dapagliflozin, empagliflozin for type 2 diabetes, and sotagliflozin for type 1 diabetes (22–24). Accumulating clinical data now show that these drugs provide benefits to diseases beyond diabetes, such as heart failure and renal diseases (25–27). In preclinical model systems, beneficial effects of SGLTi drugs were reported on a wide spectrum of diseases that include cancer, Alzheimer, atherosclerosis, and others (28–31). In a study using lung organoids we demonstrated that sotagliflozin is potentially useful for treating CF lung diseases (32).

In the present work, we found an upregulation of SGLT1 in different tissues, including the liver of CF rabbits and human CF airway lineage cells. This observation raised the question of whether inhibition of SGLT1 would benefit CF and specifically CFLD. To answer this, we treated CF rabbits with the most potent clinically approved SGLT1i, sotagliflozin, and found that the drug provided systematic benefits, and particularly attenuated liver disease. Intriguingly, our data showed that sotagliflozin treatment represses hepatic endoplasmic reticulum (ER) stress and the associated inflammatory responses. The present work not only provides preclinical support for repurposing sotagliflozin to treat CFLD, but also has implications for expanding this class of drugs to treat other ER stress– and inflammation-related liver diseases such as nonalcoholic steatohepatitis (NASH).

## Results

### SGLT1 is upregulated in CF rabbit tissues and CF patient–derived cells.

We recently generated a CF rabbit line (CF-9) that carries a 9-base pair (9-bp) deletion in the CFTR gene leading to the deletion of 3 amino acids (P477, S478, and E479) in nucleotide-binding domain 1 (NBD1) of the protein (11). We reported that CF-9 rabbits (interchangeably referred to as “CF rabbits” hereafter; other CFTR mutation types are specified) exhibit many typical CF phenotypes, such as growth retardation, intestinal obstruction, airway abnormalities, and liver disorders (11, 13).

**Figure 1:**
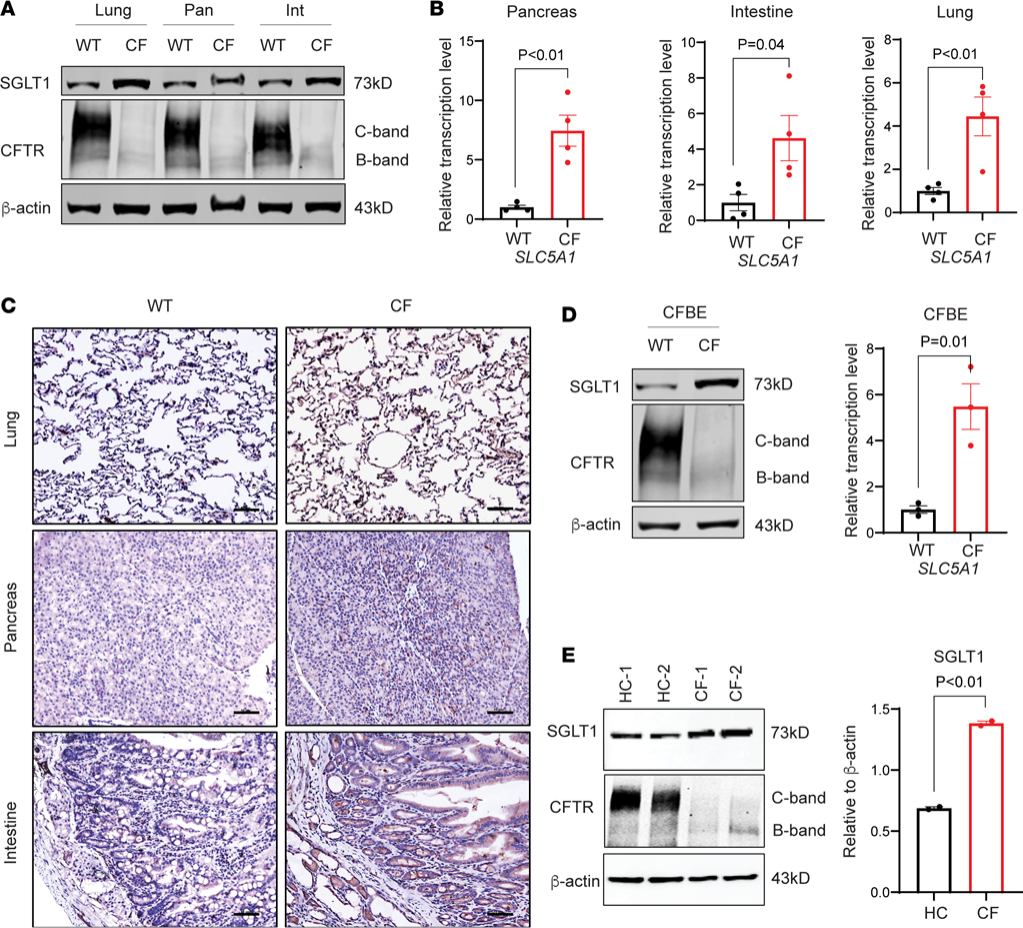
SGLT1 is upregulated in CF conditions. (**A**) Western blot of SGLT1 and CFTR in the lung, pancreas (pan), and intestine (int) in the wild-type (WT) and CF-9 (CF) rabbits. (**B**) RT-qPCR of *SLC5A1* in the lung, pancreas, and intestine in the WT (*n* = 4) and CF (*n* = 4) rabbits. Data were analyzed and compared using unpaired, 2-tailed Student’s *t* test. (**C**) Immunohistochemical staining of SGLT1 in the lung, pancreas, and intestine of the WT and CF rabbits. Scale bars: 50 μm. (**D**) Western blot (left) of SGLT1 and RT-qPCR (right) of *SLC5A1* in CF patient–derived bronchial epithelial (CFBE) cells. The RT-qPCR data (*n* = 3) were analyzed and compared using unpaired, 2-tailed Student’s *t* test. (**E**) Western blot (left) and quantification (right) of SGLT1 in CFBE cells. The quantification data (*n* = 2) were analyzed and compared using unpaired, 2-tailed Student’s *t* test. HC-1 and -2, healthy controls; CF-1 and -2, CF patients with homozygous F508del mutation.

We measured the SGLT1 expression by Western blotting, quantitative real-time PCR (RT-qPCR), and immunohistochemistry (IHC) staining analyses in the lung, pancreas, intestine, and liver in CF rabbits. We discovered that SGLT1 was universally upregulated in all these tissues in the CF-9 rabbits (Figure 1, A–C), as well as in those of CF rabbits carrying different CFTR mutations, which include a CF-1 line that mimics a class I null mutation (11) and the recently developed CFTR-F508del line (12) that represents the most dominant patient mutation (Supplemental Figure 1, A and B; supplemental material available online with this article; https://doi.org/10.1172/jci.insight.165826DS1). In contrast, while the SGLT2 expression was highly abundant in the kidney tissue, as expected, it was very low in the CF-affected organs (Supplemental Figure 1C). Based on these observations, our studies were focused on SGLT1.

As observed in CF rabbits and consistent with our prior report (32), the SGLT1 signal was high in CF patient–derived bronchial epithelial cells carrying the CFTR-F508del mutation, but low in the cells with the WT CFTR genotype (Figure 1D). Similarly, in patient primary cell–derived airway epithelial cells, the SGLT1 level was higher in the CF patients with the CFTR-F508del mutation than those in the healthy control individuals (Figure 1E). Together, these data reveal a robust upregulation of SGLT1 in CF tissues and cells. Such upregulation exists in individuals of different CFTR mutation types and is conserved in rabbits and humans.

### Sotagliflozin extended the lifespan of CF rabbits.

The reciprocal relationship between SGLT1 and CFTR in CF individuals led us to hypothesize that pharmacological inhibition of SGLT1 may be beneficial to CF. To test this, we selected sotagliflorizin to treat CF rabbits. Sotagliflorizin has a dual SGLT1/2 inhibition capacity, with the IC_50_ for SGLT1 and SGLT2 at 36 nM and 1.8 nM, respectively (33). Of note, it is the most potent SGLT1i of all clinically approved SGLTi drugs (Supplemental Table 1).

Oral administration of sotagliflozin in a capsule (15 mg/kg, once daily) to a WT rabbit led to an immediate spike of the urine glucose level on the next day, which returned to normal soon after drug withdrawal, confirming that the drug is effective in rabbits (Supplemental Figure 2A). In the following experiments, we used the same dose and delivery route to treat CF rabbits. Animals in the treatment group received sotagliflozin in a capsule daily, whereas animals in the control group received an empty capsule daily (i.e., vehicle control).

**Figure 2:**
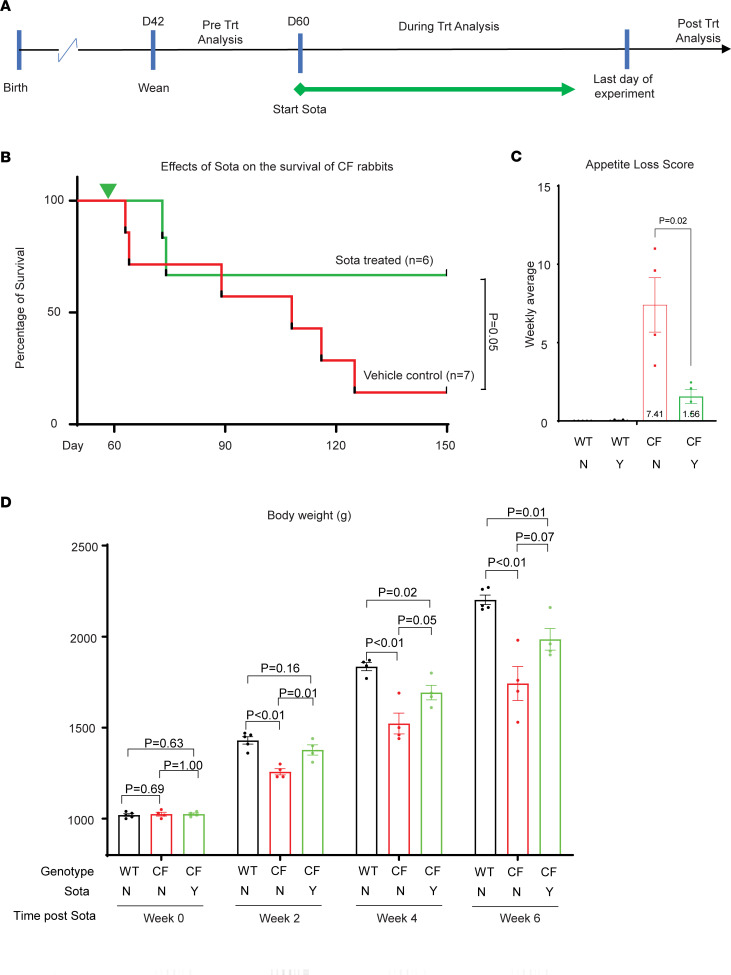
Sotagliflozin promotes weight gain and extends lifespan of CF rabbits. (**A**) Illustration of the sotagliflozin treatment regime. Trt, treatment; Sota, sotagliflozin. (**B**) Survival curve of CF rabbits treated with (*n* = 6) or without (*n* = 7) sotagliflozin. The survival rates were analyzed and compared by the χ^2^ test. (**C**) Appetite loss scores of CF rabbits treated (*n* = 4) with or without (*n* = 4) sotagliflozin and WT rabbits treated with (*n* = 5) or without (*n* = 5) sotagliflozin. The data were analyzed and compared using unpaired, 2-tailed Student’s *t* test. (**D**) Body weight changes of the WT rabbits without sotagliflozin treatment (*n* = 4) and the CF rabbits treated with (*n* = 4) or without (*n* = 4) sotagliflozin. The data were analyzed and compared using unpaired, 2-tailed Student’s *t* test.

We started the sotagliflozin treatment in a cohort of CF rabbits at age 60.5 ± 0.7 days (*n* = 6). The animals were treated until they were removed due to moribund conditions or until they reached the experiment end point (when the animal reached 150 days of age or older) (Figure 2A). A much higher survival rate until 150 days of age was observed in sotagliflozin-treated CF rabbits when compared with that of a cohort of age-matched control vehicle-treated CF rabbits (*n* = 7) (Figure 2B). Four out of 6 sotagliflozin-treated CF rabbits (67%) lived to the experiment end point (between 150 and 200 days of age, without signs of morbidity, euthanized for sample collection), in comparison with only 14% (1 of 7) animals in the nontreated group that lived until 150 days of age (Figure 2B). These findings demonstrate that sotagliflozin treatment initiated in 60-day-old CF rabbits can greatly extend their lifespan.

### Sotagliflozin promoted appetite and weight gain of CF rabbits.

As a SGLT1/2 dual inhibitor, sotagliflozin blocks glucose absorption in the intestines (through SGLT1) and glucose reabsorption in the renal tubules (through SGLT1 and 2). This raised the concern that the drug may worsen CF patients’ nutritional conditions. To address this concern, we monitored food intake and body weight gain of CF rabbits treated with sotagliflozin (*n* = 4) in comparison to the control CF rabbits without sotagliflozin (*n* = 4).

Food intake was monitored by a daily loss of appetite (LOA) score: a zero LOA score indicates a full consumption of the food, whereas a high LOA score indicates severe LOA. WT rabbits had close to zero LOA days throughout the experiment, regardless of sotagliflozin treatment (Figure 2C). While high LOA scores were recorded for CF rabbits without sotagliflozin, CF rabbits treated with sotagliflozin had a much reduced LOA score (Figure 2C), indicating that sotagliflozin has an appetite-promoting effect. Consequently, a positive impact of sotagliflozin on the weight gain was observed. Starting at a similar weight, sotagliflozin-treated CF rabbits grew much faster, on average, weighing 9.5%, 11.2%, and 13.9% more than control vehicle-treated CF rabbits at 2, 4, and 6 weeks after treatment, respectively (Figure 2D).

To gain insights into the mechanistic basis by which sotagliflozin promotes appetite and weight gain, we examined the fecal microbiota as a proxy for the intestinal microbiome composition. In a previous study, we reported that CF rabbits suffer from intestinal dysbiosis characterized by significantly lower numbers of observed species and decreased species richness in comparison with those of the WT animals (14). Recent studies suggest that SGLT inhibition could alter the composition of the intestinal microbiome (34). Therefore, we compared the fecal microbiota between CF rabbits treated with sotagliflozin to those without. While it was confirmed that CF rabbits suffer from intestinal dysbiosis, no effects of sotagliflozin on the diversity and richness of the intestine microbiome were observed at this stage (Supplemental Figure 2, B and C).

Next, we measured the serum lipase activity (SLA) in these CF rabbits. In our previous work, we noticed a lower SLA level in the CF rabbits than that in the WT ones and speculated that this contributed to the malnutritional phenotype of CF rabbits (11). Confirming this earlier finding, CF rabbits had lower SLA levels than WT rabbits prior to the drug treatment (Supplemental Figure 2D). Intriguingly, sotagliflozin treatment normalized the SLA in CF rabbits to a level comparable to that of the WT, whereas in the nontreated rabbits the SLA level remained low (Supplemental Figure 2D). This provided an explanation, at least partially, for how sotagliflozin improved the weight gain of the CF rabbits.

Taken together, these findings suggest that sotagliflozin promoted the appetite and weight gain in young CF rabbits. The data should alleviate any concerns that sotagliflozin may adversely affect young CF individuals’ nutritional intake and growth.

### Sotagliflozin does not induce spontaneous lung infections in CF rabbits.

Pulmonary disease is the leading cause of morbidity and mortality in CF patients. Because sotagliflozin is an inhibitor of the glucose transporter SGLT1, which is expressed in the airway epithelial cells (35), one concern of using this drug for CF is that the elevated glucose level in the airway lumen may aggravate the lung infections. Our prior work showed that spontaneous lung infections are rare events in CF rabbits, likely due to the still relatively short lifespan (11). Nevertheless, we collected bronchoalveolar lavage (BAL) fluid (BALF) from rabbits treated with or without sotagliflozin. While the BALF glucose levels were slightly higher in both the WT and CF rabbits that received the drug treatment than those without, which is in line with the effect of SGLT inhibition, they were all in a normal low-level range, between 0.3 and 0.7 mg/dL (Supplemental Figure 2E). Furthermore, when the BALFs from the sotagliflozin-treated rabbits were plated on culture dishes, no bacterial growth was identified, similarly to those from the rabbits without sotagliflozin treatment (Supplemental Figure 2F). Together, these results confirm that sotagliflozin does not induce bacterial infections in CF rabbits.

### Homeostatic effects of sotagliflozin on the blood chemistry parameters of CF rabbits.

**Figure 3:**
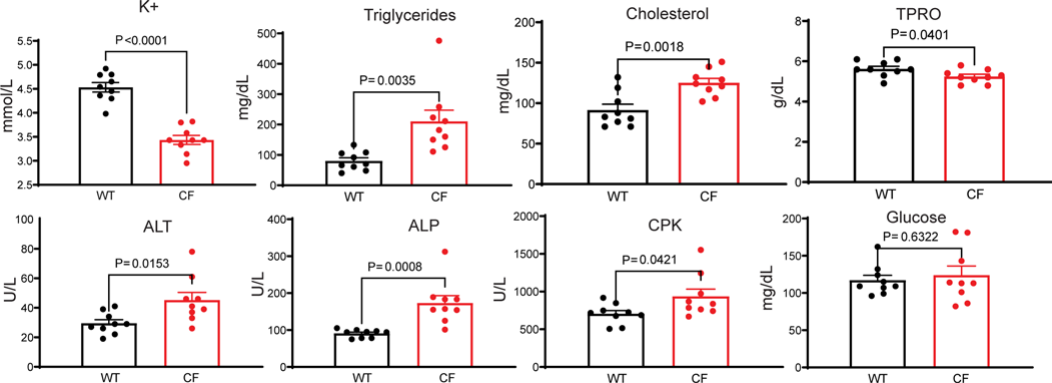
Effects of sotagliflozin on the blood chemistry parameters of CF rabbits. Blood chemistry values of K^+^, triglycerides, cholesterol, TPRO, ALT, ALP, CPK, and glucose of WT (*n* = 9) and CF (*n* = 9) rabbits prior to sotagliflozin treatment. The data were analyzed and compared using unpaired, 2-tailed Student’s *t* test.

Many CF patients show abnormalities in their blood chemistry parameters (BCPs) (36). CF rabbits, in comparison with WT rabbits, had many abnormal BCPs, which included lower levels of potassium (K^+^), higher levels of triglyceride and cholesterol, elevated alanine aminotransferase (ALT), alkaline phosphatase (ALP), and creatine phosphokinase (CPK), and decreased total protein (TPRO) concentrations (Figure 3 and Supplemental Figure 3).

To evaluate sotagliflozin’s effect on BCPs, blood samples were collected and the BCP values were compared between the animals treated with or without the drug. Sotagliflozin had minimal impact on the BCPs of the WT animals (Supplemental Table 2). In the CF animals without sotagliflozin treatment, many parameters deteriorated along the time course, as indicated by gradual deviations from the normal range (Supplemental Figure 3), consistent with the disease progression process. In contrast, many BCPs of the CF animals treated with sotagliflozin remained unchanged or even improved (Table 1 and Supplemental Figure 3). The BCPs in sotagliflozin-treated CF rabbits, reflected by the levels of K^+^, Ca^2+^, triglycerides, cholesterol, ALT, and TPRO, were significantly improved compared with those of the CF animals without sotagliflozin (Table 1). We assessed the drug’s effects on the glucose levels since it is approved in Europe for treating type 1 diabetes (37). At this stage, the effects of sotagliflozin on the blood glucose levels of CF rabbits were not apparent (Table 1). A comprehensive follow-up study to investigate the effects of this drug on CF-related diabetes is warranted. These data show that sotagliflozin has a homeostatic effect on the BCPs of CF rabbits, suggesting that the drug may benefit CF individuals in terms of electrolyte balance, lipid metabolism, and liver function.

### SGLT1 is expressed in the hepatocytes of CF rabbits.

**Figure 4:**
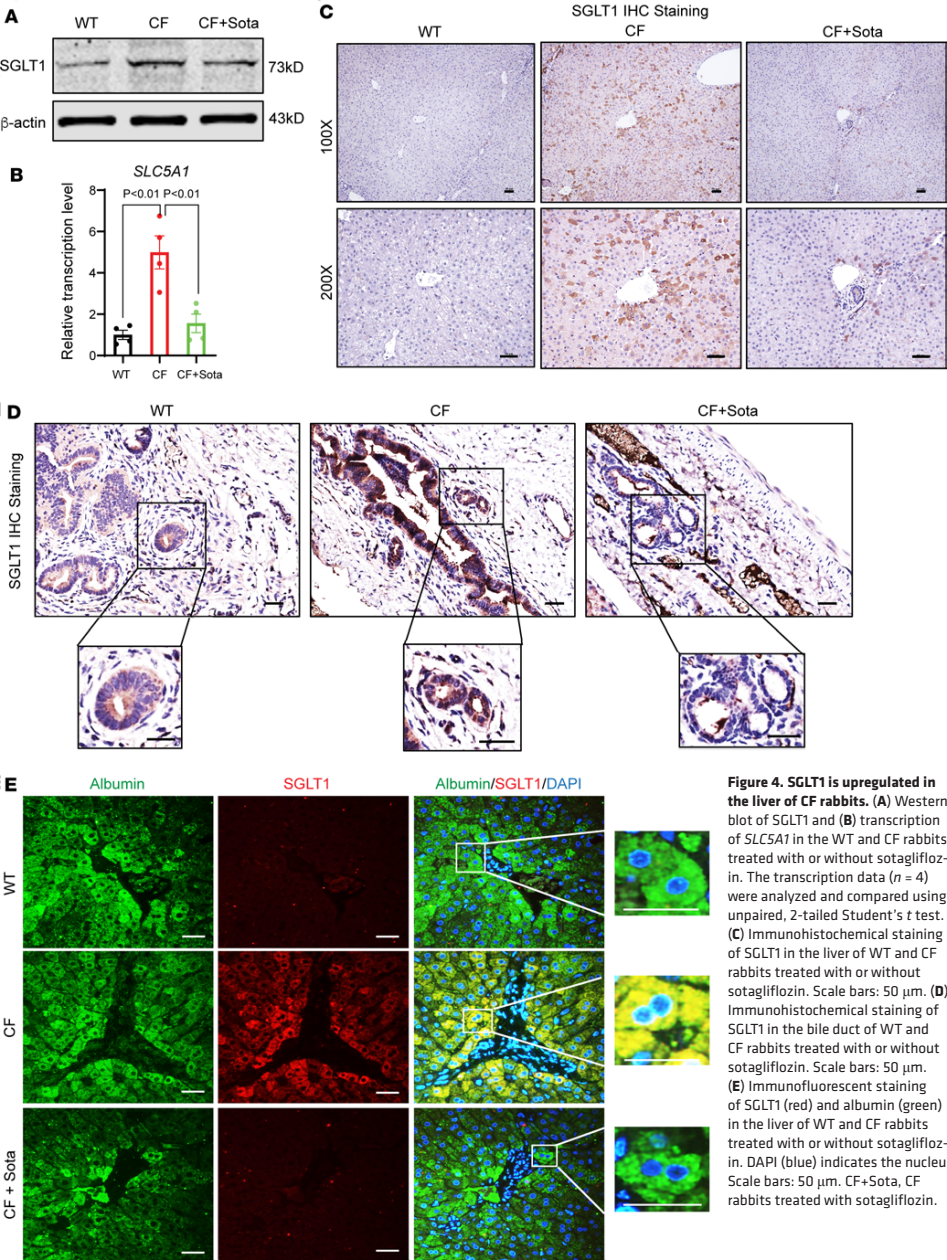
SGLT1 is upregulated in the liver of CF rabbits. (**A**) Western blot of SGLT1 and (**B**) transcription of *SLC5A1* in the WT and CF rabbits treated with or without sotagliflozin. The transcription data (*n* = 4) were analyzed and compared using unpaired, 2-tailed Student’s *t* test. (**C**) Immunohistochemical staining of SGLT1 in the liver of WT and CF rabbits treated with or without sotagliflozin. Scale bars: 50 μm. (**D**) Immunohistochemical staining of SGLT1 in the bile duct of WT and CF rabbits treated with or without sotagliflozin. Scale bars: 50 μm. (**E**) Immunofluorescent staining of SGLT1 (red) and albumin (green) in the liver of WT and CF rabbits treated with or without sotagliflozin. DAPI (blue) indicates the nucleus. Scale bars: 50 μm. CF+Sota, CF rabbits treated with sotagliflozin.

We next examined the effects of sotagliflozin on the livers of CF rabbits. Several SGLTi drugs, including sotagliflozin, have gained major regulatory agencies’ approval for treating diabetes. Some of these have shown unexpected beneficial effects on cardiovascular and renal disease risks (22–24), and on non-CF liver diseases (38–42). As shown in Figure 4, A–C, SGLT1 was upregulated in the CF rabbit livers. We then delineated the SGLT1 expression profile at the cell-type level, specifically in the hepatocytes and the bile duct epithelial cells (cholangiocytes). Although it is known that SGLT1 is expressed in cholangiocytes (43), it is less clear whether it is expressed in hepatocytes. As expected, SGLT1 signals were much higher in the cholangiocytes of CF than that in the WT rabbits, as determined by IHC staining (Figure 4D). However, while the SGLT1 signal was barely detectable in the liver sections of WT rabbits, abundant SGLT signals were detected in the livers of CF rabbits, and colocalized with the hepatocyte marker albumin (Figure 4E). In contrast, hepatic SGLT2 signal levels were very low in both the WT and CF rabbits (Supplemental Figure 4C). These findings suggest that, under CF pathological conditions, SGLT1 is upregulated in cholangiocytes and hepatocytes, implicating SGLT1 as a potential therapeutic target for CFLD.

### Sotagliflozin alleviates NASH-like phenotypes in CF rabbits.

**Figure 5:**
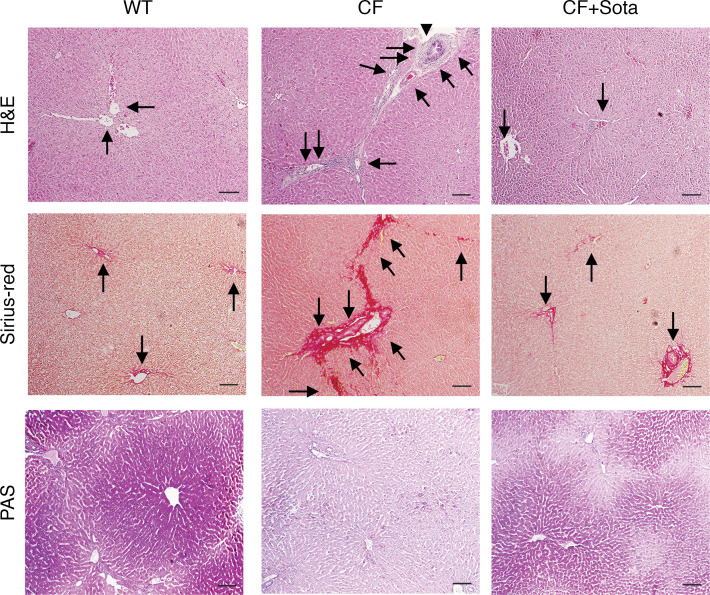
Sotagliflozin improves NASH-like phenotypes in the liver of CF rabbits. H&E, Sirius red, and PAS staining of CF rabbits treated with or without sotagliflozin. In the H&E panel, arrows indicate inflammatory infiltration, and arrowheads indicate Mallory bodies. In the H&E panel, the arrows point to the representative duct structures with or without biliary cirrhosis. In the Sirius red panel, arrows indicate normal glycogen or perivenular fibrosis (WT or CF+Sota group) and portal or bridging fibrosis (CF group). The PAS panel shows the content and distribution of hepatic glycogens (red aggregate staining) across WT, CF, and CF+Sota groups. In Supplemental Figure 5, low- and medium-magnification images for H&E, Sirus red, and PAS staining are provided for overviews of the changes in content and distribution of hepatic glycogens and fibrosis across the groups. CF+Sota, CF rabbits treated with sotagliflozin. Scale bars: 20 μm.

Many CF patients develop hepatic steatosis and NASH associated with multifactorial etiologies (44, 45). We determined whether a NASH-like phenotype developed in CF rabbits, and whether sotagliflozin treatment has any effect. Based on H&E staining of the liver tissue sections and Sirius red staining of the hepatic collagen deposition, we identified increased hepatic steatosis, lobular and portal inflammation, as well as portal and bridging fibrosis in the liver of CF rabbits, as compared with WT controls (Figure 5 and Supplemental Figure 5). Periodic acid–Schiff (PAS) staining revealed diminished hepatic glycogen storage in CF rabbits (Figure 5 and Supplemental Figure 5). By a quantitative analysis of NASH grading and staging scores (46, 47), we confirmed that many CF rabbits developed a profound NASH-like phenotype, characterized by hepatic steatosis, hepatic inflammation, and portal and bridging fibrosis (Figure 5, Table 2, and Supplemental Figure 5).

Notably, sotagliflozin treatment of the CF rabbits alleviated hepatic fibrosis and enhanced hepatic glycogen storage capacity, as compared with the livers of the animals treated with vehicle control (Figure 5 and Supplemental Figure 5). Consistently, the nonalcoholic fatty liver disease (NAFLD) grading and staging scoring showed that the NASH-relevant parameters, including lobular inflammation, portal inflammation, lobular necroinflammation, Mallory bodies, and fibrosis stage were all significantly improved in the sotagliflozin-treated CF rabbit livers, compared with those in the vehicle-treated group (Table 2). Interestingly, the drug treatment led to reduced levels of SGLT1 protein as well as *SLC5A1* transcripts in the CF rabbit livers (Figure 4, A–E). Together, these data demonstrate beneficial effects of sotagliflozin on alleviating NASH-like phenotypes in CF rabbit livers.

### Beneficial effect of sotagliflozin on the bile duct and bile acid profiles in CF rabbits.

CF rabbits developed CF-associated focal biliary fibrosis and cirrhosis around the bile duct accompanied with mucus plug, as shown by H&E staining and Sirius red staining of collagens (Figure 5). Sotagliflozin treatment led to signs of alleviated biliary cirrhosis and inflammation, and diminished viscous mucus plug in CF rabbits (Figure 5).

**Figure 6:**
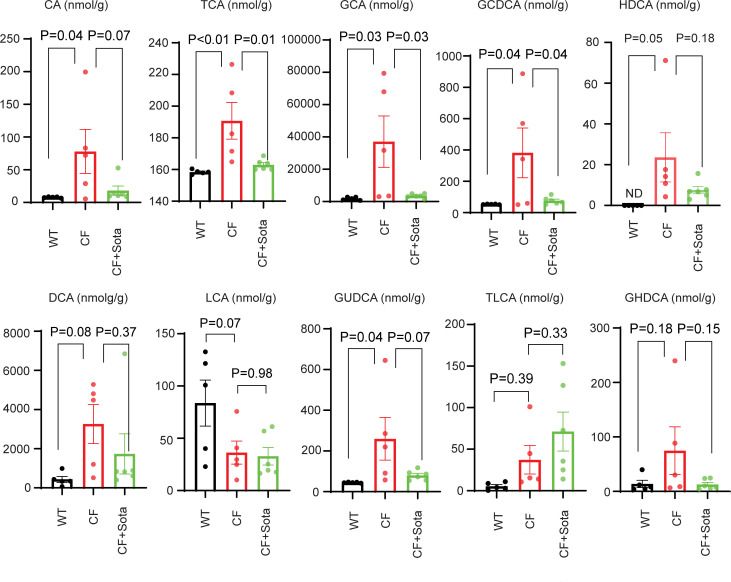
Sotagliflozin improves bile acid homeostasis in the liver of CF rabbits. The values of major bile acid species from the WT (*n* = 5) rabbits, and CF rabbits treated (*n* = 6) with or without (*n* = 5) sotagliflozin are shown. The data were analyzed and compared using unpaired, 2-tailed Student’s *t* test. CA, cholic acid; TCA, taurocholic acid; GCA, glycocholic acid; GCDCA, glycochenodeoxycholic acid; HDCA, hyodeoxycholic acid; DCA, deoxycholic acid; LCA, lithocholic acid; GUDCA, glycoursodeoxycholic acid; TLCA, taurolithocholic acid; GHDCA, glycohyodeoxycholic acid; CF+Sota, CF rabbits treated with sotagliflozin.

Bile acid (BA) dysregulation contributes to the development of CFLD (48). BA-targeted metabolomics analysis showed that most BA species were altered in either the bile (Supplemental Figure 6) or liver samples (Figure 6) of the CF rabbits, compared with those of WT animals. To evaluate the effects of sotagliflozin on BA species, we collected bile fluid and liver tissues from age-matched CF rabbits treated with sotagliflozin or vehicle control (Supplemental Table 3). Prominent improvement was observed in the liver BA profile of CF rabbits treated with sotagliflozin (Figure 6). All the primary BAs, including cholic acid (CA), taurocholic acid (TCA), glycocholic acid (GCA), and glycochenodeoxycholic acid (GCDCA) were highly elevated in the livers of the CF rabbits. With sotagliflozin treatment, these values were normalized to levels comparable to those in the WT animals. Similar effects were observed on many secondary BAs, including hyodeoxycholic acid (HDCA), deoxycholic acid (DCA), glycoursodeoxycholic acid (GUDCA), and glycohyodeoxycholic acid (GHDCA). While the effects of sotagliflozin treatment on the BA profile of the bile fluid were not as robust as on those of the liver samples, we did observe a trend of improvement with taurolithocholic acid (TLCA), HDCA, and GUDCA (Supplemental Figure 6). These data indicate that sotagliflozin treatment can mitigate hepatic biliary cirrhosis and restore BA homeostasis in the bile fluid and more obviously in the liver of CF rabbits.

### Sotagliflozin alleviates ER stress in CF rabbit livers.

ER stress and the unfolded protein response (UPR) contribute to the pathogenesis in CF lung and liver diseases (13, 49, 50). Consistently, we recently reported the observation of elevation of ER stress and the UPR in CF rabbit livers (13). The positive correlation between the levels of SGLT1 and the extent of ER stress response in CF rabbit livers prompted us to determine whether inhibiting SGLT1 with sotagliflozin has any effects on the otherwise elevated ER stress responses.

**Figure 7:**
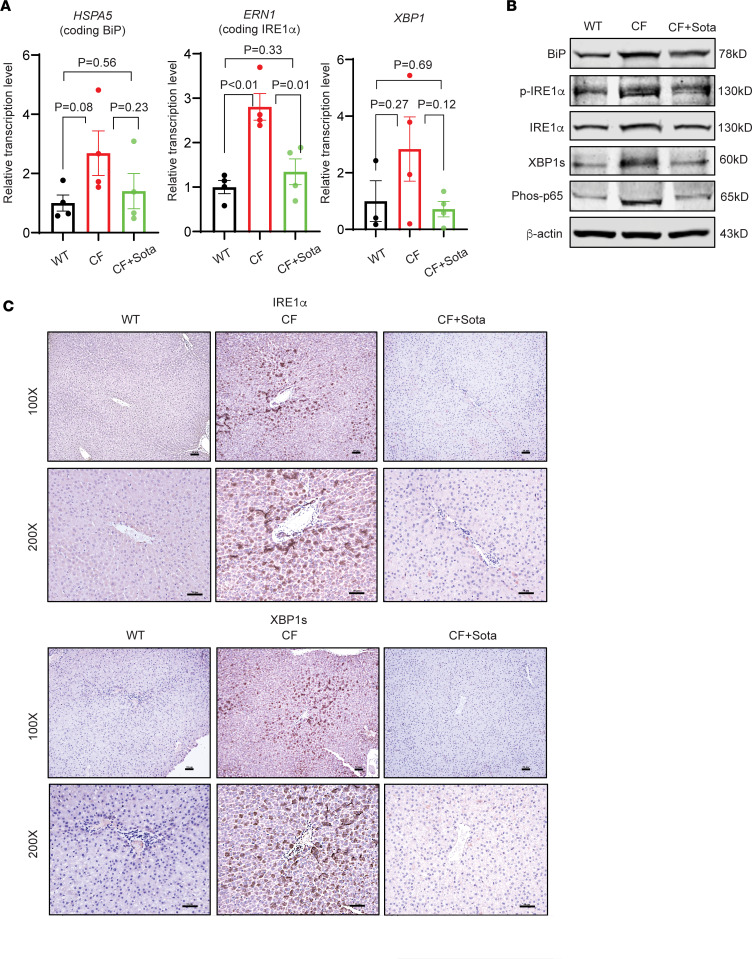
Sotagliflozin attenuates ER stress in the liver of CF rabbits. (**A**) RT-qPCR of *HSPA5*, *ERN1*, and *XBP1* in the liver tissues of WT rabbits (*n* = 4), and CF rabbits treated with (*n* = 4) or without (*n* = 4) sotagliflozin. The data were analyzed and compared using unpaired, 2-tailed Student’s *t* test. (**B**) Western blot of ER stress and inflammation markers BiP, p-IRE1α, IRE1α, XBP1s, and p-p65 in the liver tissues of WT and CF rabbits treated with or without sotagliflozin. (**C**) IHC staining of IRE1α and XBP1s in the liver tissues of CF rabbits treated with or without sotagliflozin. Scale bars: 50 μm. CF+Sota, CF rabbits treated with sotagliflozin.

We conducted IHC, Western blotting, and RT-qPCR assays to determine the main markers of the canonical BiP/IRE1α/XBP1–mediated UPR pathway in the CF rabbit livers. The ER stress markers in the IRE1α/XBP1–mediated UPR pathway (Figure 7, A–C), along with SGLT1 (Figure 4), were upregulated in the liver of CF rabbits. The sotagliflozin treatment effectively reduced these ER stress markers (Figure 7, A–C) and SGLT1 (Figure 4) to levels comparable to those of the WT animals. Of note, sotagliflozin treatment also reduced ER-associated protein degradation–associated (ERAD-associated) E3 ligase HRD1, the target of the IRE1α/XBP1 UPR pathway (51) (Supplemental Figure 7A). Interestingly, other ER stress markers, including *ATF4* and *HSP90B1*, were elevated in the liver of CF rabbits; however, sotagliflozin treatment did not have major effects on modulating them in the CF rabbit livers (Supplemental Figure 7B).

Furthermore, we examined the activation of a major inflammatory pathway, mediated by NF-κB, in CF rabbit livers in the presence or absence of sotagliflozin treatment. Compared with WT, levels of phosphorylated p65 protein, the indicator of the NF-κB–mediated inflammatory pathway, were increased in rabbit livers (Figure 7B). Upon sotagliflozin treatment, the levels of phosphorylated p65 were decreased. The levels of 2 inflammatory cytokines, *TNF* and *IL6*, in sotagliflozin-treated CF rabbit livers displayed a similar pattern of reduction by sotagliflozin treatment, although the differences were not statistically significant (Supplemental Figure 7C). These data indicate that ER stress and inflammatory responses prevail in CF rabbit livers and that sotagliflozin treatment may improve CFLD-like phenotypes in CF animals through modulating ER stress and inflammatory responses.

We also examined expression of major glucose and lipid metabolic genes in the livers of CF rabbits in the presence or absence of sotagliflozin treatment (Supplemental Figure 8). Expression of the gene encoding the rate-limiting enzyme in gluconeogenesis, phosphoenolpyruvate carboxykinase 1 (*PCK1*), was decreased in the CF rabbit livers, but sotagliflozin treatment significantly increased the expression of hepatic *PCK1* (Supplemental Figure 8). Expression of the key enzyme of glycogen synthesis, glycogen synthase 2 (*GYS2*), was decreased in CF rabbit livers, while sotagliflozin treatment increased hepatic *GYS2* expression in CF rabbits (Supplemental Figure 8). Additionally, we examined the expression of a key enzyme in bile acid synthesis, cytochrome P450 family 7 subfamily A member 1 (CYP7A1) (52), and major metabolic regulators of fatty acid oxidation and gluconeogenesis, including FGF21 and PPARα (53–55). Sotagliflozin treatment normalized the expression of the corresponding coding genes *CYP7A1*, *FGF21*, and *PPARA* in the livers of CF rabbits (Supplemental Figure 8).

### Sotagliflozin mitigates ER stress in major nonhepatic organs in CF rabbits.

We next examined the effect of sotagliflozin on the levels of SGLT1 and major ER stress markers in major nonhepatic CF-affected organs, including the lungs, intestine, and pancreas. Similar to the findings in the liver, markedly elevated levels of ER stress markers, including BiP, phosphorylated IRE1α (p-IRE1α), and the activated form of XBP1 (XBP1s), along with the increased SGLT1 signals, were observed in the lungs, intestine, and pancreas of the CF rabbits without sotagliflozin treatment (Supplemental Figures 9–11). Likewise, sotagliflozin effectively reduced these ER stress markers and SGLT1 in these nonhepatic organs of CF rabbits (Supplemental Figures 9–11). These findings demonstrate the effectiveness of sotagliflozin in repressing the ER stress response in nonhepatic, CF-affected organs.

## Discussion

CFLD is the third-ranking cause of mortality in CF, affecting one-third of patients. Ursodeoxycholic acid (UDCA) is being used to treat CFLD; however, its efficacy is limited (56). Many late-stage CFLD patients require and undergo liver transplantation. In October 2019, the FDA approved Trikafta, which greatly improves patients’ pulmonary functions (4), leading to improved lifespan. The effects of Trikafta on extrapulmonary organs, however, remain to be fully investigated. Some studies have suggested that Trikafta may impose an adverse burden on the CF patient liver (5–10). Indeed, given the potential of hepatotoxicity reported in 10%–15% of patients treated with modulator therapies, these agents are not recommended to patients with advanced liver disease, as described in the Trikafta product label (57). Furthermore, there are many CF patients who do not benefit from or do not have access to Trikafta (58). Taking these together, how to mitigate liver disease in patients treated with or without Trikafta is a priority subject for CF translational research.

The present work provides preclinical support to repurpose sotagliflozin for treating CFLD. Sotagliflozin has been tested in multiple clinical trials and has shown a high range of safe dosage, up to 2,000 mg in a single dose (59). Recent clinical trials have shown that sotagliflozin provided unexpected benefits to diseases beyond diabetes, such as renal and cardiovascular diseases (26, 60). How this drug affects CF individuals has not been investigated. As shown by this work, the improvement in liver-related conditions in CF rabbits with sotagliflozin treatment was unexpected and exciting; it normalizes many BCPs, as well as the concentrations of BA species in both the liver and the bile fluid, and it attenuates liver fibrosis and other NASH-related phenotypes. Together, these orchestrate a major benefit of sotagliflozin treatment for the liver, metabolic conditions, and general health under CF deficiency.

How SGLTi drugs, such as sotagliflozin, benefit diseases beyond diabetes, such as CFLD, remain elusive. In the present work, we investigated ER stress response pathways underlying CF deficiency and the beneficial effects of sotagliflozin treatment. The crosstalk between ER stress and inflammatory responses facilitates the progression of metabolic diseases, including obesity, diabetes, atherosclerosis, and liver diseases (61–63). Both ER stress and inflammation are hallmarks for many CF-related symptoms (49, 64). We demonstrate that sotagliflozin is effective in alleviating the otherwise abnormally elevated ER stress in major CF organs, including the lungs, intestine, pancreas, and liver, especially in the IRE1α-mediated UPR pathway. It is known that IRE1α, the primary UPR transducer, is activated to promote NF-κB and interferon pathways under inflammatory conditions (63, 65). Activation of IRE1α increases the production of the proinflammatory cytokines (i.e., IL-1β, TNF, IL-6) in response to ER stress through splicing of the mRNA encoding XBP1, which encodes a potential transcription factor to drive proinflammatory cytokine expression (66–68). Our data show that sotagliflozin treatment reduced the levels of NF-κB, significantly ameliorated lobular and portal inflammation, and attenuated the expression of cytokines, included TNF-α and IL-6 in CF rabbit liver tissues. Such antiinflammatory effects of sotagliflozin are consistent with other reports of SGLTis in cardiovascular and renal disease (69, 70). Moreover, the ER stress–attenuating effect by sotagliflozin was observed in all organs examined, which could explain the observation of improved conditions in multiple organs of CF rabbits, as well as the extended lifespan.

**Figure 8:**
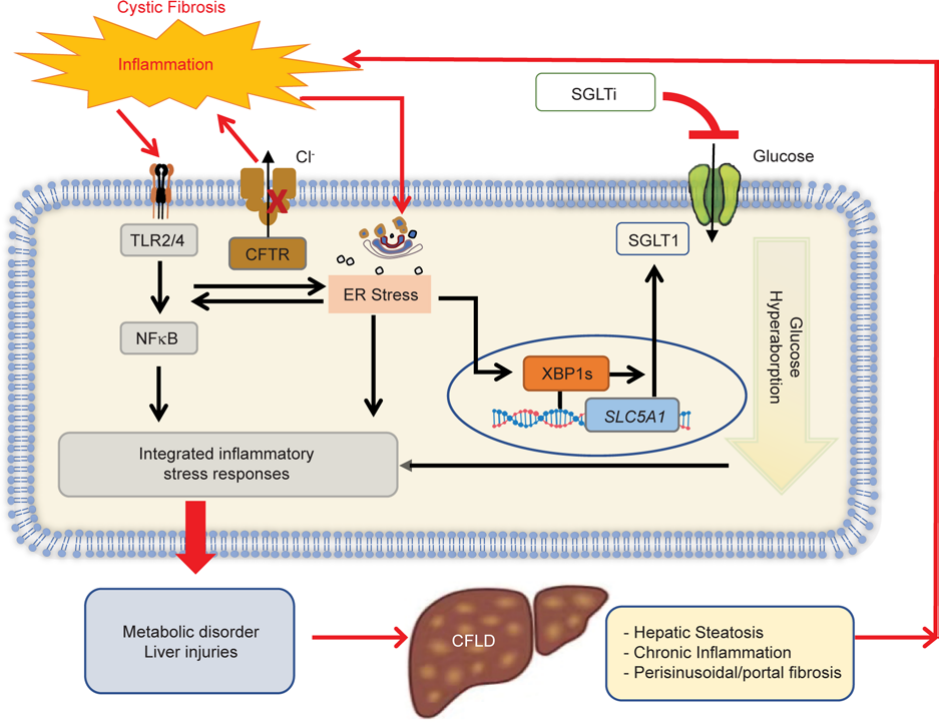
Proposed mechanism of action of how SGLT inhibition benefits CFLD. A viscous circle is formed by CFTR mutation → inflammation and ER stress → SGLT1 upregulation → liver-associated disorders → CFLD. SGLT1 inhibition achieved with sotagliflozin disrupts this circle and is therefore beneficial for CF liver.

One interesting and unexpected finding is that sotagliflozin treatment reduced the level of SGLT1 in CF rabbit liver and other tissues. It remains to be tested how this channel-blocking drug leads to changes in its target protein at the transcriptional and translational levels. Nevertheless, this observation suggests that there exists a viscous circle of CF → ER stress → SGLT1 upregulation → CF in the liver and other affected tissues (Figure 8). Thus, pharmacological disruption of this circle, as achieved with sotagliflozin, could provide hepatic and global benefits to CF individuals.

The observation of upregulated SGLT1 in CF rabbit hepatocytes is worth noting. Because CFTR is not expressed in hepatocytes in healthy or CF individuals, hepatocytes have been understudied in CFLD. SGLT1 was not expected to be expressed in hepatocytes either, but our data show that under pathological conditions such as CF, SGLT1 is activated in hepatocytes. This finding suggests that the upregulated SGLT1 in CF hepatocytes represents what we believe is a novel etiology factor for CFLD, in addition to the prevailing theory of bile duct obstruction caused by the loss of CFTR function in cholangiocytes. We reason that sotagliflozin treatment might have worked through both avenues, i.e., improving the ion channel homeostasis of the bile duct epithelial cells on one hand, and suppressing the hyperabsorption of glucose by the hepatocytes on the other. These remain to be investigated in follow-up studies.

Regarding the safety and side effects of the sotagliflozin treatment in CF, the present work did not identify any major concerns. We paid particular attention to 2 aspects. First, we investigated whether sotagliflozin adversely affects nutritional intake of CF individuals. The data demonstrate the opposite, i.e., the drug promoted young CF rabbits’ appetite and weight gain, which could be partially explained by the back-to-normal serum lipase levels in sotagliflozin-treated animals. Secondly, we investigated whether the sotagliflozin treatment would lead to elevated glucose levels in the airway lumen and consequently increase incidence of bacterial infection. The data show that the drug has minimal effects on glucose levels in the BALF, and none of the CF rabbits, regardless of sotagliflozin treatment, showed signs of lung infection. Nevertheless, the safety as well as efficacy parameters should be evaluated in additional preclinical models, such as another CF animal model and CF patient–derived cellular models.

We would like to point out several limitations of the present work. First, while our data support the notion that sotagliflozin works through inhibiting SGLT1 in CF rabbits, we cannot exclude the contribution from inhibiting SGLT2. Sotagliflozin is the most effective clinically approved SGLT1i (Supplemental Table 1), but its IC_50_ for SGLT1 (36 nM) is still 20-fold higher than that of SGLT2 (1.8 nM). The SGLT2 inhibition by sotagliflozin in renal tubule cells may have contributed to the observed phenotypes, especially glucose-related changes in sotagliflozin-treated animals. For the future work, development of a *SLC5A1*/*CFTR* double-knockout rabbit model may help definitively delineate the contributions of SGLT1 or SGLT2. Secondly, the present work uses CF rabbits with a mutation (i.e., CF-9) that is not present in CF patients. To further confirm our observations, it is necessary to evaluate sotagliflozin in the recently developed CFTR-F508del rabbits (12) and in other CF animals carrying clinically relevant mutations. Thirdly, in the present work, we did not treat the CF rabbits with Trikafta. Given that most CF patients are already taking or will be taking Trikafta, it is imperative to evaluate the combinational effects of sotagliflozin in CF rabbits treated with Trikafta. Fourthly, the present work focused on the liver-related pathologies. It should be noted that the systematic improvement of CF rabbit conditions after sotagliflozin treatment likely comes not only from the liver but also from other organs/systems. For example, we demonstrated that sotagliflozin alleviated the otherwise elevated ER stress response in the lungs, pancreas, and intestine. Many metabolic and electrolytic parameters were normalized in sotagliflozin-treated CF rabbits evidenced by the blood chemistry data. Follow-up work is needed (and ongoing) to investigate the beneficial effects of sotagliflozin beyond the liver in CF individuals. In addition, although the present work monitored the effects of sotagliflozin on the CF rabbits for several months, it is still a short-term study, considering that CF patients live for decades. A follow-up study in the scale of years in CF rabbits will provide valuable preclinical knowledge toward the long-term effects of the drug on CF liver conditions such as steatotic and cholestatic abnormalities, as well as other organ systems.

In summary, the present work demonstrates that SGLT1 is upregulated in both the liver and the other CF-affected organs of CF rabbits. Sotagliflozin treatment benefits CF rabbits globally, as evidenced by better appetite scores and weight gain, improved BCPs, and elongated lifespan. In particular, the drug attenuated NASH phenotypes, including fibrosis, and normalized BA species in both the liver and bile. Furthermore, we show that the integrated ER stress and inflammatory responses represent a major driving force in the pathogenesis of CFLD, and that sotagliflozin treatment benefits CFLD by relieving the hepatic ER stress and inflammatory responses. Our work merits future preclinical and clinical evaluations of sotagliflozin’s efficacy and safety in treating CFLD.

## Methods

### Sex as a biological variable

Animals of both sexes were used; however, sex was not considered as a biological variable in the present work.

### Animals

WT and CF rabbits were used in the study. The CF rabbits were developed and maintained as described in a previous study (11). WT and CF rabbits, after weaning, were randomly divided into groups of control and treatment. The rabbits in treatment groups received sotagliflozin (15 mg/kg/day, Sun-Shine Chemical Technology Co., Ltd) at 60 days old by daily gavage. If the CF animals in either treatment group or control group were in good general condition after 4 weeks of treatment, then the treatment was extended as long as possible; 150 days of age was used to as the cutoff day to calculate the survival rate.

Animals were monitored daily for general conditions, including the following: veterinarian score, body weight, food intake, body temperature, and behavior. LOA score was recorded daily: a score of 0 indicates no food left (i.e., completion consumption of the food); a score of 1 indicates 25% of food left; a score of 2 indicates 50% of food left; a score of 3 indicates 75% of food left; and a score of 4 indicates all food was left (i.e., no consumption at all). Seven consecutive daily LOA scores were added as the weekly accumulated LOA score. The weekly average LOA score was calculated as the average of all available weekly accumulated LOA scores for each animal.

The full chemistry panel in serum, including liver/kidney function, glucose, lipid profiles, and electrolytes before and after treatment were performed by the In-Vivo Animal Core at the University of Michigan. The blood samples were collected from animals after overnight fasting. Feces were collected before the treatment and at the end of the experiments for microbiome analysis. Upon euthanasia, BALF was collected for glucose and microbiological analyses. Tissues including lung, liver, pancreas, and intestine were banked for histopathology and RT-qPCR/protein analysis.

### Chemicals and reagents

Chemicals were purchased from Sigma-Aldrich unless indicated otherwise. CFTR antibodies were obtained from the CF foundation. Antibodies against GRP78, IRE1α, XBP1s, and β-actin were purchased from Cell Signaling Technology. SGLT1 antibodies were purchased from Abcam and Invitrogen. The specificity of the SGLT1 antibody for IHC staining was confirmed by using an IgG isotype control, a secondary antibody–only control, and by using a nontargeting primary antibody control (Supplemental Figure 4). The secondary antibodies were from LI-COR Biosciences and Jackson ImmunoResearch Laboratories. The PAS staining kit was purchased from Thermo Fisher Scientific. The assay kits and antibody information are listed in Supplemental Table 4.

### Cell culture

CF bronchial epithelial cells expressing WT CFTR were cultured in MEM with 10% FBS and 0.5 μg/mL puromycin at 37°C with 5% CO_2_ in a humidified incubator. CF bronchial epithelial cells expressing CFTR-F508del were cultured in MEM with 10% FBS and 1 μg/mL puromycin.

### Examination of SGLT1 on healthy control and CF human epithelial cells

Human adult airway basal cells were isolated from fresh discarded lung tissues at Massachusetts General Hospital under IRB-approved protocols (nos. 2017P001479 and 2013P002332). Healthy control and CF airway basal cells (harboring CFTR-F508del/F508del) at their earliest passage were used to avoid cell expansion–associated effects. Mature airway epithelial cells were generated on an air-liquid interface using a previously reported protocol (71, 72). The cell lysates were prepared from airway liquid interface culture at 16 days for Western blotting analysis to quantify SGLT1 expression.

### Histology

All histology slide examinations and scorings were performed in a “grouped masking” manner unless otherwise stated. In a grouped masking, the examiner/scorer had information regarding the study groups but did not know the specific treatments for each group (73).

#### H&E, PAS, and Sirius red staining.

Liver tissues from WT and CF rabbits were fixed with 4% neutral paraformaldehyde and embedded in paraffin. The tissues were sectioned at 5 μm thick and subjected to H&E, PAS, or Sirius red staining as described previously (74).

#### Histological scoring for NASH activities.

Hepatic steatosis, hepatocyte ballooning, lobular and portal inflammation, Mallory bodies, and fibrosis were examined and scored according to the modified Brunt scoring system for NAFLD (46, 75). The grade scores were calculated based on the scores of steatosis, hepatocyte ballooning, lobular and portal inflammation, and Mallory bodies. The stage scores (0–3) were based on the liver fibrosis: 0, none; 1, mild; 2, moderate; and 3, severe. The fibrosis stages were determined based on the 0–4 stage system: 0, none; 1, zone 3 perisinusoidal fibrosis; 2, zone 3 perisinusoidal fibrosis plus portal fibrosis; 3, perisinusoidal fibrosis, portal fibrosis, plus bridging fibrosis; and 4, cirrhosis.

### BALF collection and bacterial culture

BALF collection was conducted as described in our previous study (11). In brief, the chest cavity of rabbits was opened under anesthesia. BAL was performed on the right lobes by instilling and retrieving sterile PBS (10 mL) 3 times with a syringe (retrieved volume approximately 7 mL). The supernatant of 5 mL of BALF after centrifugation at 3,000*g* for 10 minutes was stored at –80°C for glucose measurement. One milliliter of BALF was centrifugated at 3,000*g* at 4°C for 10 minutes, the supernatant was aspirated down to 100 μL, and the pellet resuspended and then inoculate on Columbia blood agar plates for bacterial culture. BALF samples using 200, 100, and 50 μL from severe combined immunodeficiency rabbits (SCID rabbits) were inoculated on culture plates as a positive control (76). Sterile PBS (200 μL) was used as a negative control.

### Measurements of glucose and lipase

BALF was collected from overnight-fasted WT and CF rabbits with or without sotagliflozin treatment for glucose assays. Urine samples (1 mL/animal) from overnight-fasted WT rabbits were collected for glucose assays to measure urine glucose concentrations. Serum samples collected from overnight-fasted animals were used for lipase measurement. Glucose and serum lipase were measured using commercial kits according to the manufacturer’s instructions. All reagents are listed in Supplemental Table 4.

### Fecal microbiome

Night fecal samples were collected in the morning from WT and CF rabbits with or without sotagliflozin treatment, as described in our previous study (26). In brief, fecal samples from animals (300 mg) were submitted to BGI Americas Corporation for sample extraction, 16S/18S/ITS amplicon sequencing, and bioinformatics.

### Bile protein measurement and BA analysis

Total protein concentration in the bile fluid was tested using a BioTek Synergy H1 Hybrid Reader for absorbance at 280 nm. For BA analysis, 50 mg liver tissue and 50 μL bile from each rabbit were submitted to the Metabolomics Core at the University of Michigan. The methods of BA analysis in biological fluids and tissues were performed as described previously (77). The description of samples is shown in Supplemental Table 3.

### Protein extraction and Western blot analysis

Cells or rabbit tissues were lysed in RIPA lysis buffer (Thermo Fisher Scientific) with the addition of protease inhibitors (Roche) and phosphatase inhibitors (Roche). Total protein concentration was determined by Bradford Protein Assay (Bio-Rad). For CFTR Western blots, the lysates were incubated at 42°C for 15 minutes, and then resolved by 5% SDS-PAGE gels. For the Western blots of other proteins, the lysates were incubated at 95°C for 10 minutes, and then resolved by 10% SDS-PAGE gels. For both CFTR and non-CFTR proteins, nonspecific membrane sites were blocked with 5% nonfat milk in Tris-buffered saline (TBS) with 0.1% Tween 20 for 2 hours at room temperature. Blots were incubated overnight at 4°C with the appropriate primary antibodies at various dilutions shown in Supplemental Table 4. After washing, membranes were incubated with the secondary antibody for 2 hours. The intensity of the protein signals was quantified using an image processing program (LI-COR Biosciences).

### IHC staining and immunofluorescent staining

For IHC staining, the sections were dewaxed 3 times with xylene for 10 minutes each, rehydrated, and then microwaved for 20 minutes with citrate buffer pH 6.0 (Sigma-Aldrich) to retrieve the epitope, as described previously (2). The sections were incubated with animal-free blocking solution (Cell Signaling Technology) for 1 hour, washed in PBS, and then incubated with primary antibodies overnight at 4°C. After 3 washes with PBS, the sections were incubated with the Signal Stain Boost Detection Reagent (Cell Signaling Technology) for 1 hour at room temperature. The tissue sections were then washed 3 times with PBS, detected using a Pierce DAB Substrate Kit (Thermo Fisher Scientific), stained with hematoxylin, and imaged by confocal microscopy. For immunofluorescent staining, the tissue sections were incubated with primary antibodies overnight at 4°C, washed, and incubated with Alexa Fluor–labeled secondary antibodies for 1 hour at room temperature. After PBS washing, both cells and tissue sections were incubated with diluted DAPI solution for 5 minutes at room temperature, mounted with an antifade mounting media, and imaged by confocal microscopy. All antibodies are listed in Supplemental Table 4.

### RNA extraction, purification, and RT-qPCR analyses

Total RNA from rabbit tissues or CF bronchial epithelial cells was extracted using TRIzol reagent (Ambion) and purified using the RNeasy kit (QIAGEN). cDNA was then synthesized with a SuperScript III Reverse Transcriptase System (Invitrogen). The mRNA was quantified by a real-time PCR system using SYBR Green Supermix (Bio-Rad). Amplification and detection of specific mRNA products were performed with the ABI PRISM 7500 Sequence Detection System (Applied Biosystems). The real-time PCR procedure was as follows: 95°C for 2 minutes, and then 39 cycles of 95°C for 5 seconds and 60°C for 30 seconds. Quantification cycle values were acquired at the log-linear phase of amplification, and melt-curve analyses were performed to ensure the specificity of the PCR primers and amplification products. The relative expression (fold change) of the genes was normalized to the amplification amount of the housekeeping gene *GAPDH*, and calculated utilizing the 2^–ΔΔCT^ method normalized to control groups. The primer sequences are listed in Supplemental Table 5.

### Statistics

Data presented in bar graphs are expressed as mean **±** standard error of the mean (SEM) and were analyzed and compared using an unpaired, 2-tailed Student’s *t* test. The survival rates in Figure 2B were analyzed and compared by the χ^2^ test. GraphPad Prism v.9.2.0 was used for all analyses. A *P* value of less than 0.05 was considered significant.

### Study approval

All animal procedures were approved by the Institutional Animal Care and Use Committee (IACUC) of Wayne State University (Detroit, Michigan) and the University of Michigan (Ann Arbor, Michigan) and were performed in accordance with the institutional guidelines.

Procedures using human adult airway basal cells that were isolated from freshly discarded lung tissues were approved by the Institutional Review Board (IRB) of the Massachusetts General Hospital (Boston, Massachusetts).

### Data availability

Data presented in the current work are available in the Supporting Data Values XLS file, and upon reasonable request from the corresponding authors.

## Author contributions

XL, YEC, KZ, and JX conceived the idea. XL, YEC, FS, KZ, JPJ, and JX designed the experiments. XL, XH, MB, YS, ZS, CR, HJ, HGW, JS, DY, YG, YZ, HM, JZ, and JX performed the experiments. XL, YEC, KZ, JPJ, and JX analyzed the data and wrote the paper. With XH’s permission, XL was assigned the first position in the authorship order because XL contributed to the original idea of this work.

## Supplementary Material

Supplemental data

Unedited blot and gel images

Supporting data values

## Figures and Tables

**Table 1:**
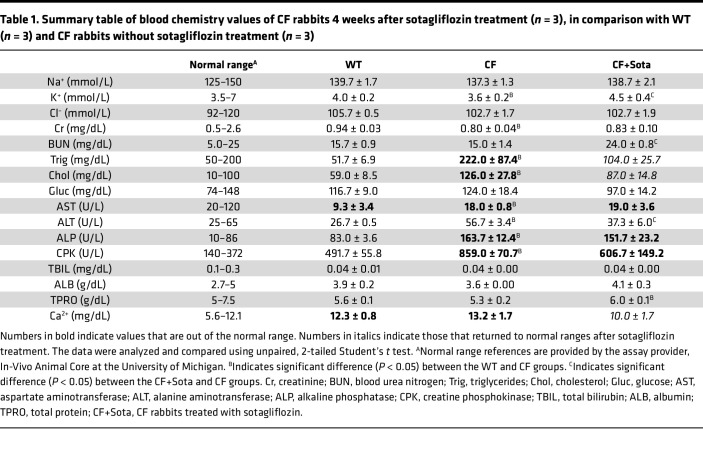
Summary table of blood chemistry values of CF rabbits 4 weeks after sotagliflozin treatment (*n* = 3), in comparison with WT (*n* = 3) and CF rabbits without sotagliflozin treatment (*n* = 3)

**Table 2:**
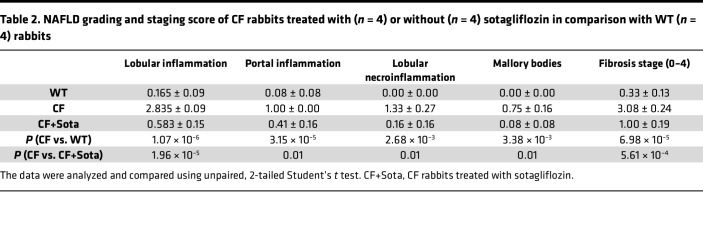
NAFLD grading and staging score of CF rabbits treated with (*n* = 4) or without (*n* = 4) sotagliflozin in comparison with WT (*n* = 4) rabbits

## Version 1 02/15/2024

In-Press Preview

## Version 2 03/22/2024

Electronic publication
