# Supplementary material for: Sotagliflozin attenuates liver-associated disorders in cystic fibrosis rabbits
Source: JCI Insight. 2024 Feb 15;9(6):e165826. doi: 10.1172/jci.insight.165826 (PMC10972622; doi:10.1172/jci.insight.165826)
Supplement: Supplemental data [file jciinsight-9-165826-s033.pdf]

Supplementary Figure 1. SGLT1 is upregulated in CF conditions. (A) Western blot of SGLT1 in the lung, pancreas, and intestine tissues of CF-1 rabbits. (B) Western blot of SGLT1 in the lung, pancreas intestine, and liver tissues of CFTR-F508del (dF) rabbits. (C) RT-qPCR of *SLC5A1* and *SLC5A2* in the intestine, pancreas, lung, liver and kidney tissues of WT (n=4) and CF-9 (n=4) rabbits. The data were analyzed and compared using unpaired, 2-tailed Student's t test.

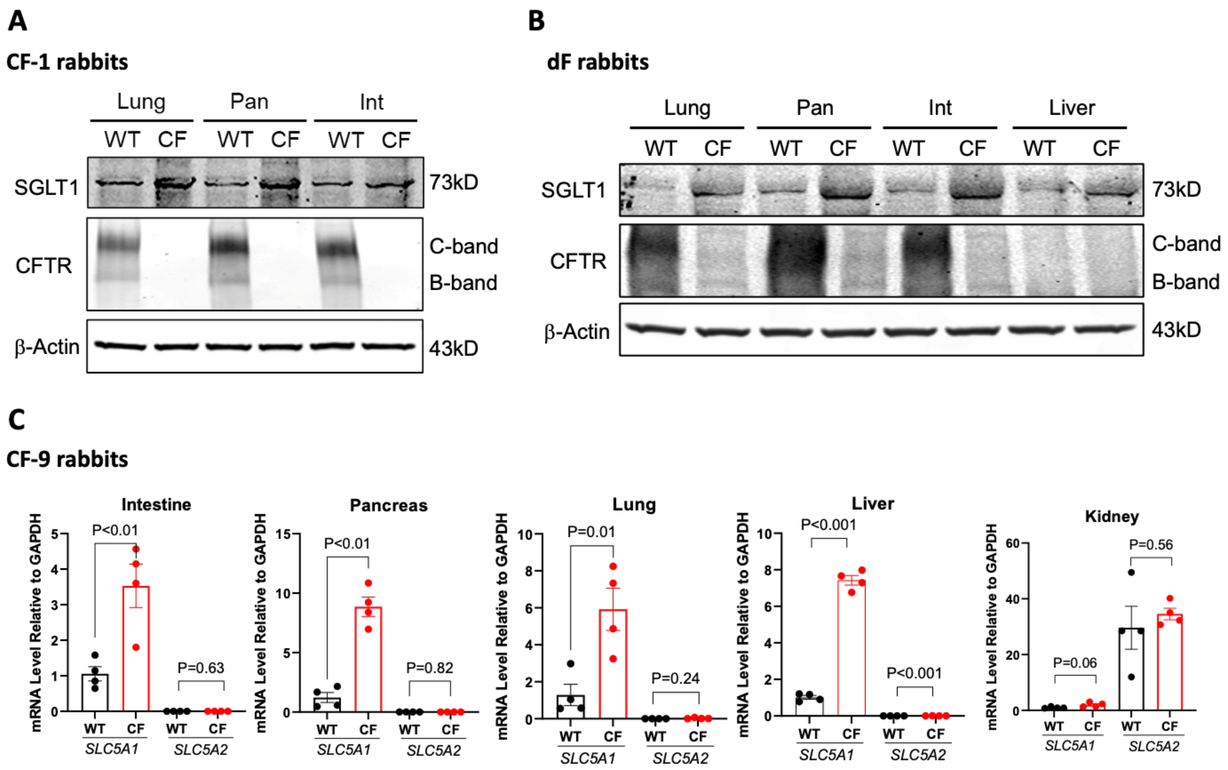

Supplementary Figure 2. Effects of Sotagliflozin on the rabbits. (A) Effect of Sotagliflozin on the urine glucose level of a WT rabbit. (B) Observed species of the feces microbiome in the WT (n=3) and CF (n=3) rabbits treated with Sotagliflozin (Sota), and WT (n=3) and CF (n=2) rabbits treated without Sotagliflozin. The data were analyzed and compared using unpaired, 2-tailed Student's t test. (C) The Shanno indexes of the feces microbiome in the WT (n=3) and CF (n=3) rabbits treated with Sotagliflozin, and WT (n=3) and CF (n=2) rabbits treated without Sotagliflozin. The data were analyzed and compared using unpaired, 2-tailed Student's t test. (D) Serum lipase activities of the CF rabbits prior to and after Sotagliflozin (Sota) treatment (n=4), in comparison to CF rabbits without (n=4) Sotagliflozin treatment. The data were analyzed and compared using unpaired, 2-tailed Student's t test. Grey box indicates the activity range of WT rabbits. (E) Glucose concentration in the Bronchoalveolar lavage (BAL) of the WT (n=5) and CF (n=6) rabbits treated with Sotagliflozin (Sota), as well as WT (n=5) and CF (n=4) rabbits without Sotagliflozin. The data were analyzed and compared using unpaired, 2-tailed Student's t test. (F) Bacterial growth after BALs from WT and CF rabbits treated with or without Sotagliflozin (Sota) were plated. N=4 animals from each group. PBS plating serves as the negative control. BALs from immunodeficient rabbits, diluted a 1x, 2x and 4x served as the positive control. Note bacterial colonies are only present in the plates plated with positive control BAL (top left three plates), but not in any other plates.

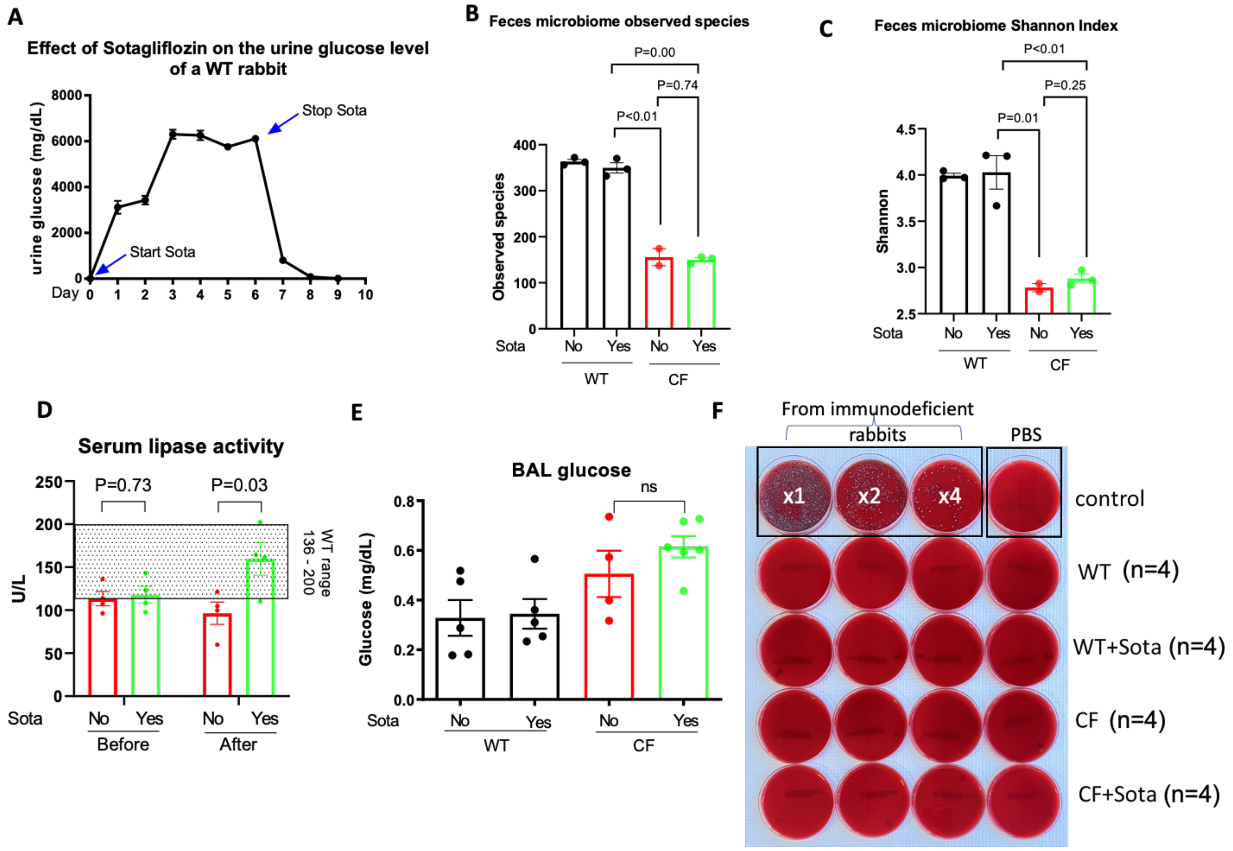

Supplementary Figure 3. Homeostasis effects of Sotagliflozin on the blood chemistry parameters of CF rabbits. Green dots: values of CF rabbits treated with Sotagliflozin (n=6). Red dots: values of CF rabbits without Sotagliflozin treatment (n=4). Not all data are available at all time points. Gray boxes indicate the normal ranges. Week 0 to 4 labeling indicates time points post Sotagliflozin treatment in the treatment group. 150 d age labeling indicates the experiment end point; at this time, all remaining CF animals, four Sotagliflozin treated and one without Sotagliflozin treatment, were sampled.

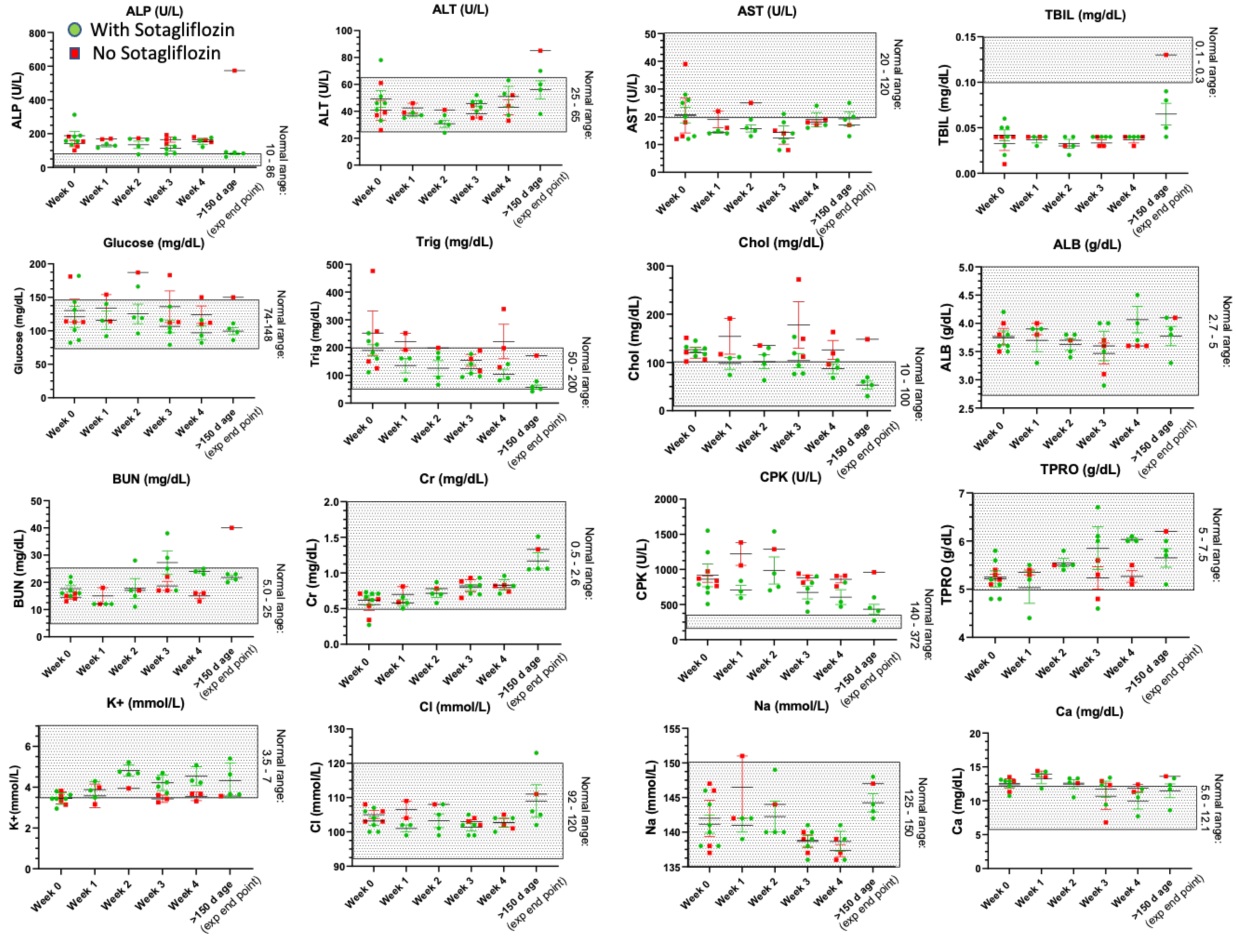

Supplementary Figure 4. Control IHC staining images to confirm the specificity of the SGLT1 antibody in WT and CF rabbit liver sections. (A) IHC staining using the IgG isotype control; Scale bar: 100 μm. (B) IHC staining using the secondary antibody-only control; Scale bar: 50 μm. (C) IHC staining of SGLT2, as a non-targeting primary antibody control. Scale bar: 50 μm.

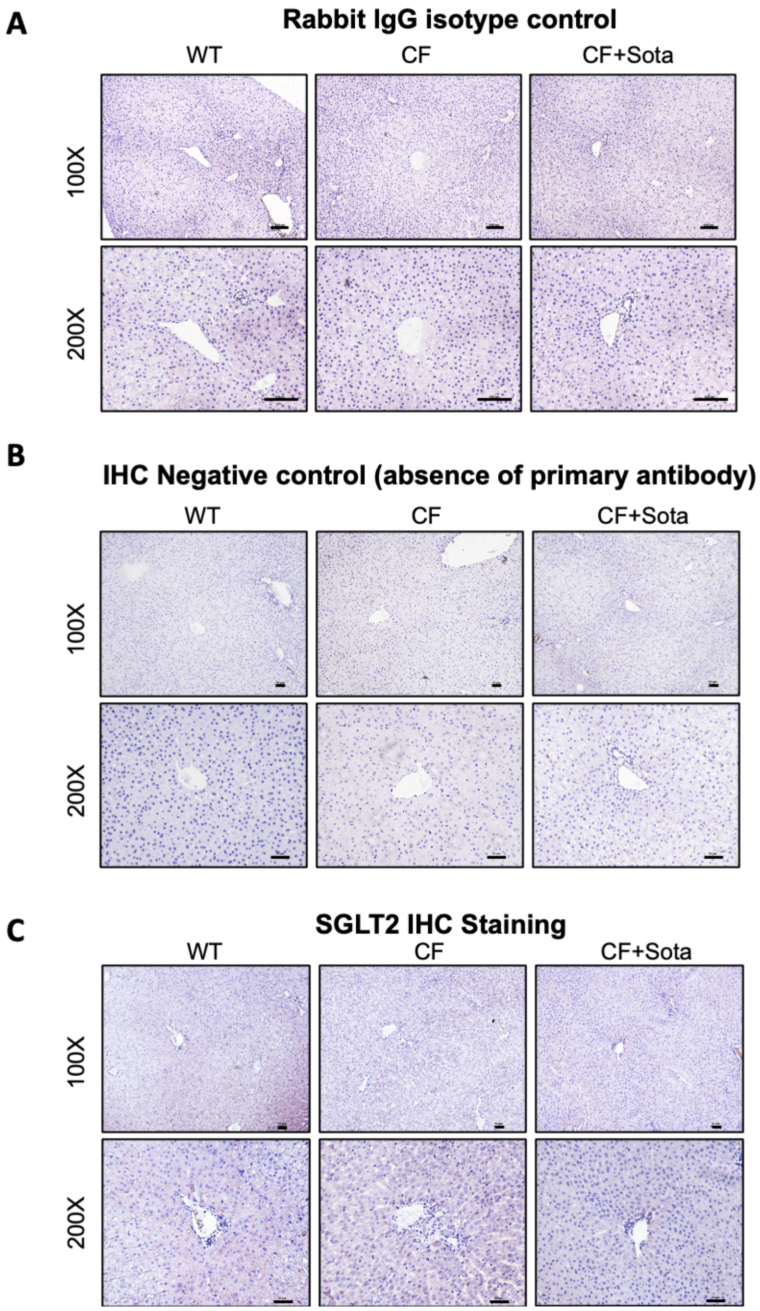

Supplementary Figure 5. Effects of Sotagliflozin on NASH-like phenotypes in the liver of CF rabbits. Top row: low magnification images of H&E staining of WT and CF rabbits treated with or without Sotagliflozin. 2nd row: low magnification images of Sirus-red staining of WT and CF rabbits treated with or without Sotagliflozin. 3rd and 4th rows: low and high magnification images of PAS staining of WT and CF rabbits treated with or without Sotagliflozin. Scale bars: 200 µm (20X); 20µm (200X).

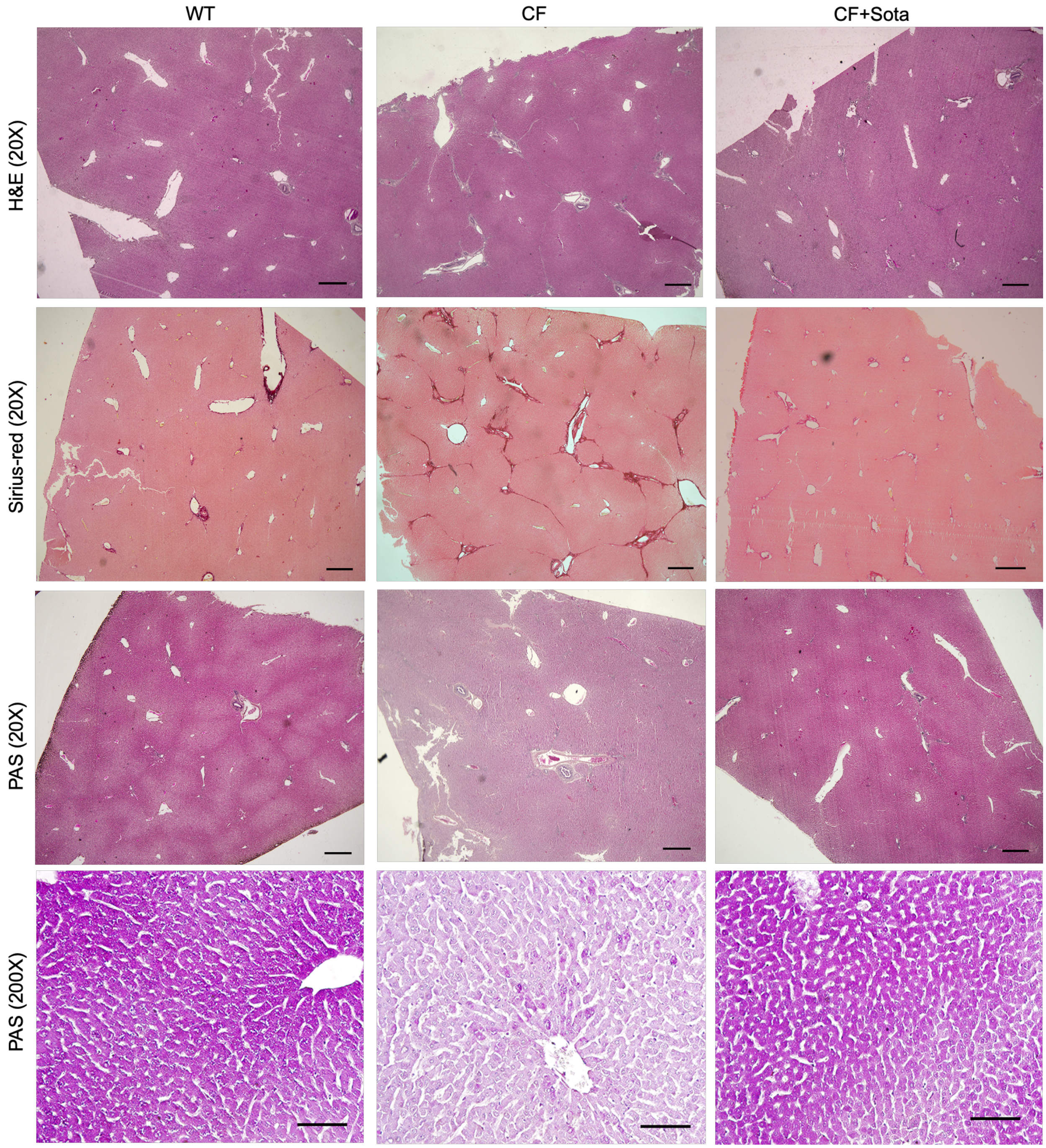

Supplementary Figure 6. Effects of Sotagliflozin on the bile acid species in the bile fluid of WT (n=5) rabbits, and CF rabbits treated with (n=4) or without (n=5) Sotagliflozin. The data were analyzed and compared using unpaired, 2-tailed Student's t test.

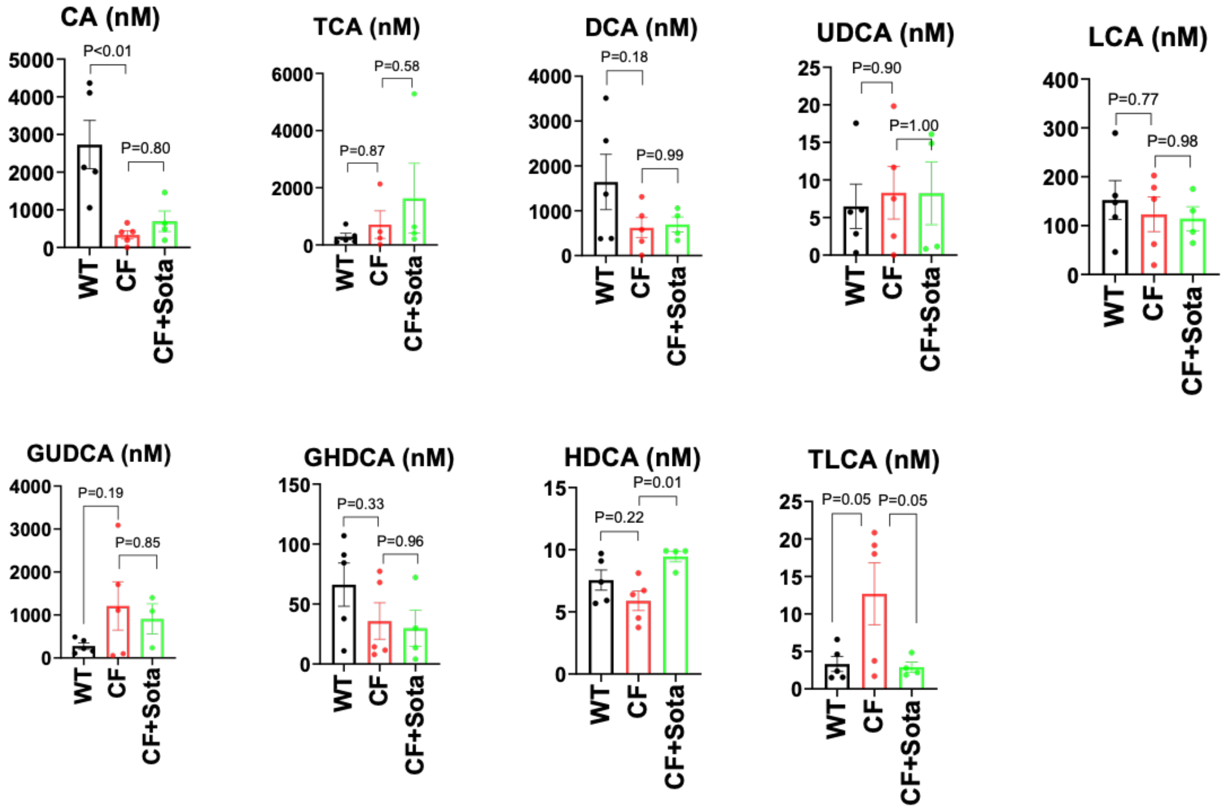

Supplementary Figure 7. **Effects of Sotagliflozin on the ER stress and inflammation related marks.** (A) Western blot of HRD1 in the liver of WT and CF rabbits treated with or without Sotagliflozin. (B) Transcription levels of ER stress related genes *ATF4* and *HSP90B1* with or without Sotagliflozin treatment (n=4). The data were analyzed and compared using unpaired, 2-tailed Student's t test. (C) Transcription levels of proinflammatory cytokines *TNF* (n=4), *LTA* (n=4), *IL1B* (n=4) and *IL6* (n=2) in the liver of CF rabbits with or without Sotagliflozin treatment. The data were analyzed and compared using unpaired, 2-tailed Student's t test.

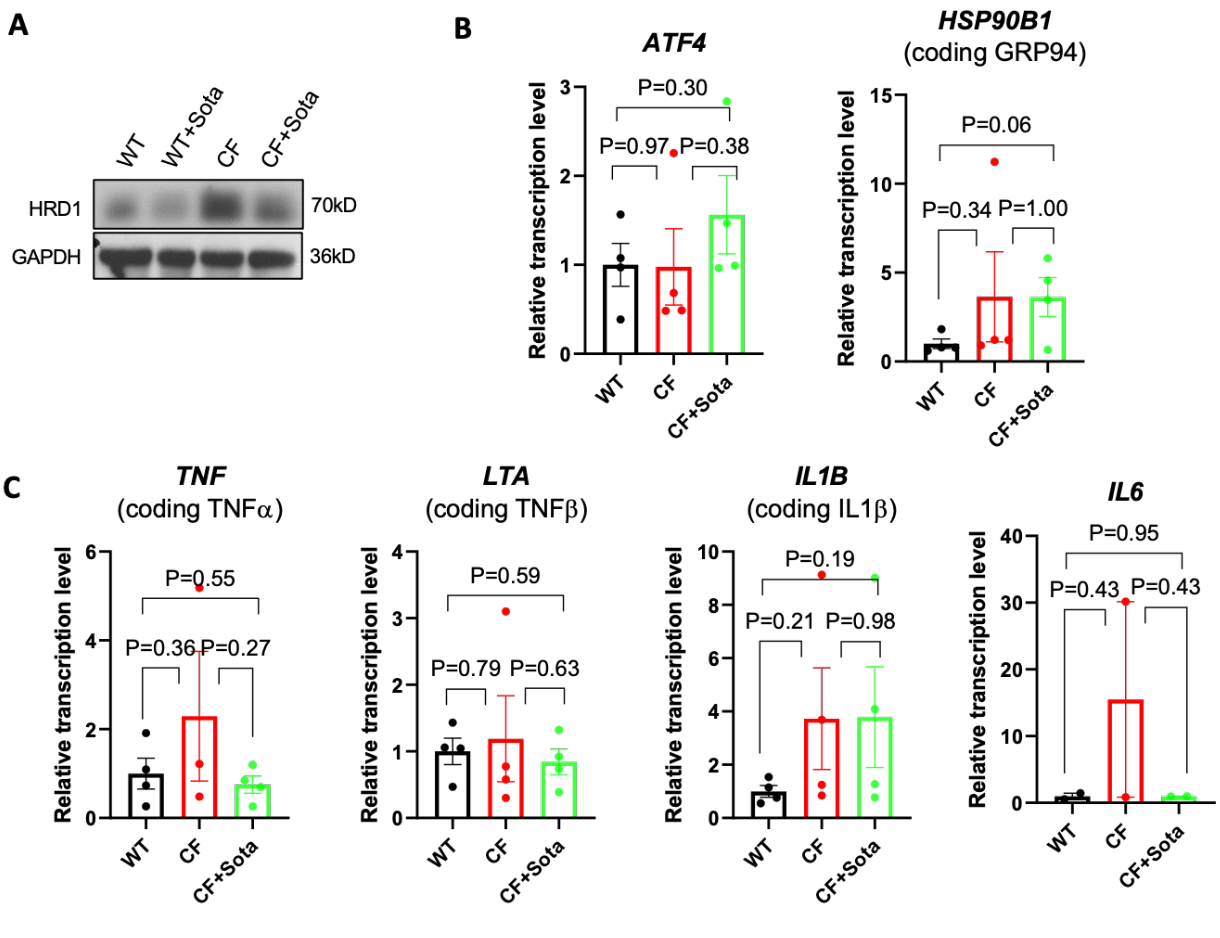

Supplementary Figure 8. Expression of glucose metabolic related genes in WT (n=4) and CF rabbits treated with (n=4) or without (n=4) Sotagliflozin (Sota). The data were analyzed and compared using unpaired, 2-tailed Student's t test.

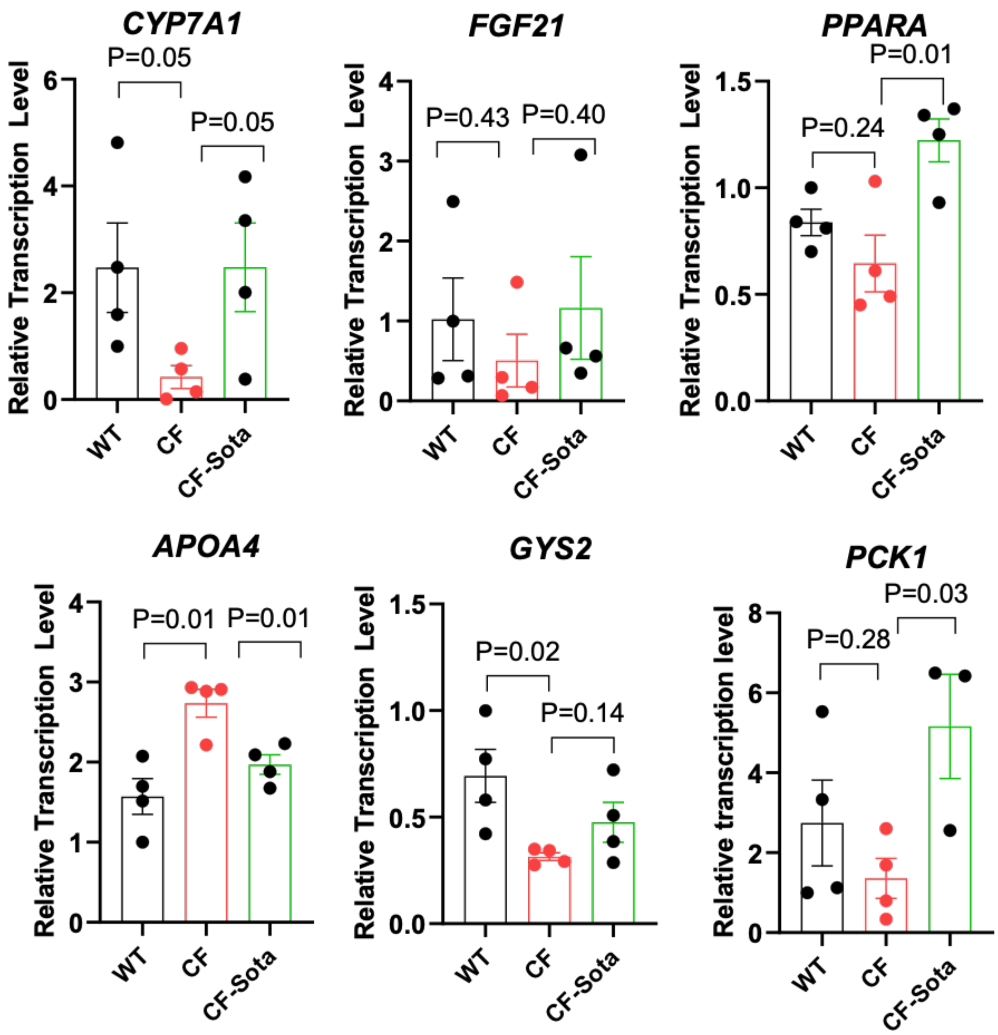

Supplementary Figure 9. Sotagliflozin attenuates ER stress in the lungs of CF rabbits. (A) RT-qPCR of *SLC5A1*, *HSPA5*, *ERN1* and *XBP1* in the lungs of WT (n=4) rabbits and CF rabbits treated with (n=4) or without (n=4) Sotagliflozin (Sota). The data were analyzed and compared using unpaired, 2-tailed Student's t test. (B) Western blot of SGLT1 and ER stress markers BiP, phosphorylated IRE1α (p-IRE1α), IRE1α, XBP1s. (C) H&E and immunohistochemistry staining of SGLT1, IRE1α and XBP1s. Scale bar: 50 μm.

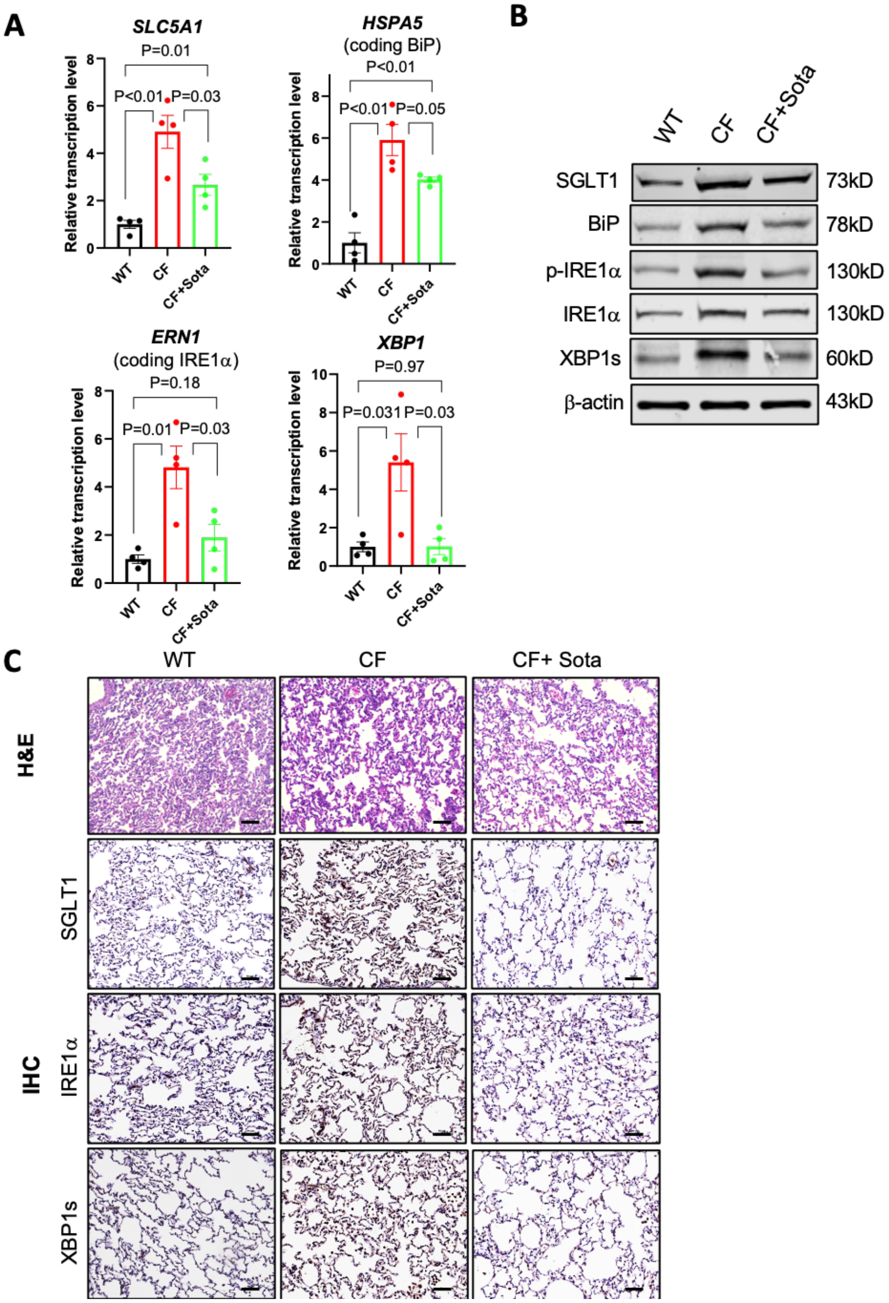

Supplementary Figure 10. Sotagliflozin attenuates ER stress in the intestine of CF rabbits. (A) RT-qPCR of *SLC5A1*, *HSPA5*, *ERN1* and *XBP1* in the intestine of WT (n=4) rabbits and CF rabbits treated with (n=4) or without (n=4) Sotagliflozin (Sota). The data were analyzed and compared using unpaired, 2-tailed Student's t test. (B) Western blot of SGLT1 and ER stress markers BiP, phosphorylated IRE1α (p-IRE1α), IRE1α, XBP1s. (C) H&E and immunohistochemistry staining of SGLT1, IRE1α and XBP1s. Scale bar: 50 μm.

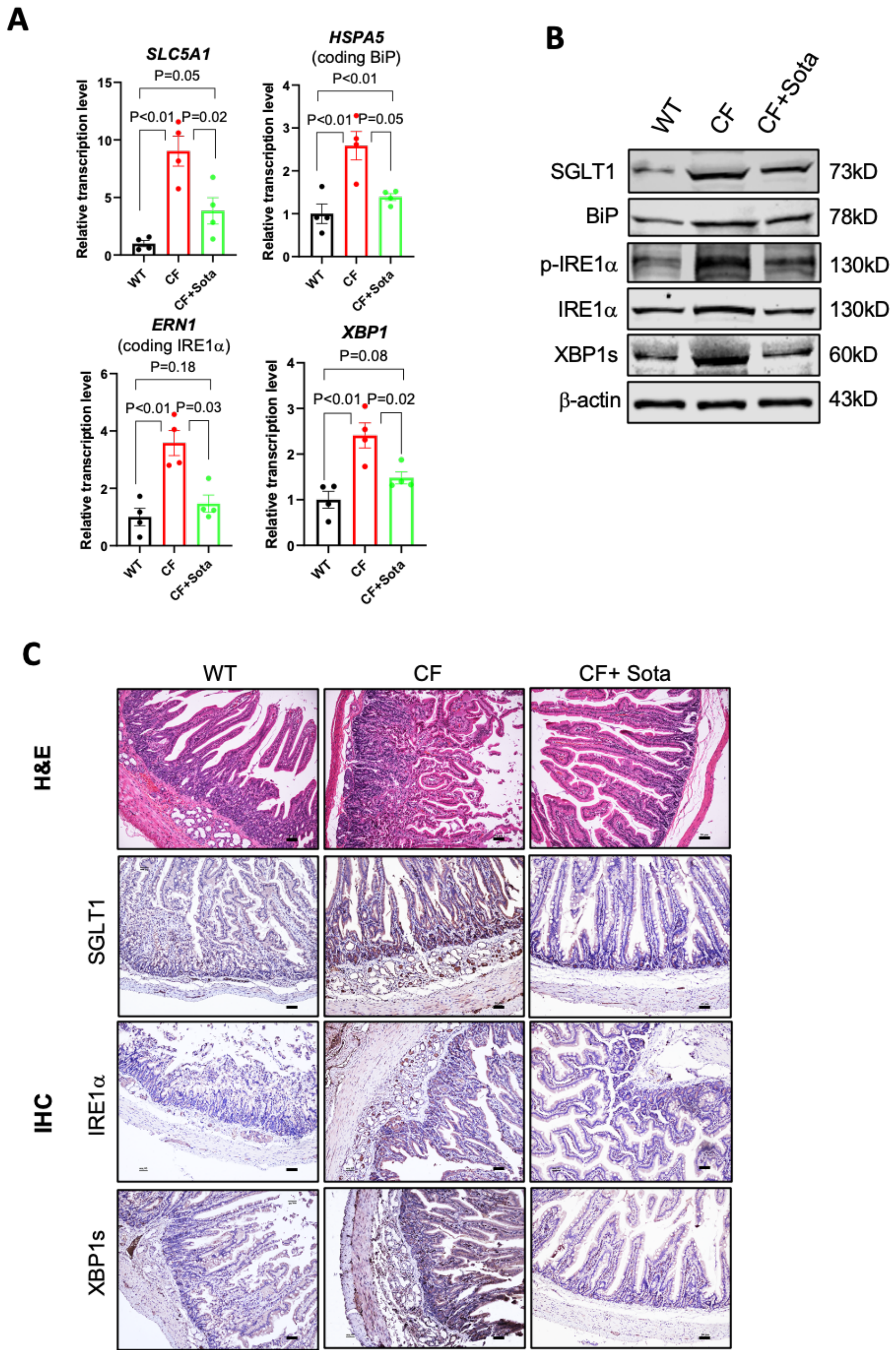

Supplementary Figure 11. Sotagliflozin attenuates ER stress in the pancreas of CF rabbits. (A) RT-qPCR of *SLC5A1*, *HSPA5*, *ERN1* and *XBP1* in the pancreas of WT (n=4) rabbits and CF rabbits treated with (n=4) or without (n=4) Sotagliflozin (Sota). The data were analyzed and compared using unpaired, 2-tailed Student's t test. (B) Western blot of SGLT1 and ER stress markers BiP, phosphorylated IRE1α (p-IRE1α), IRE1α, XBP1s. (C) H&E and immunohistochemistry staining of SGLT1, IRE1α and XBP1s. Scale bar: 50 μm.

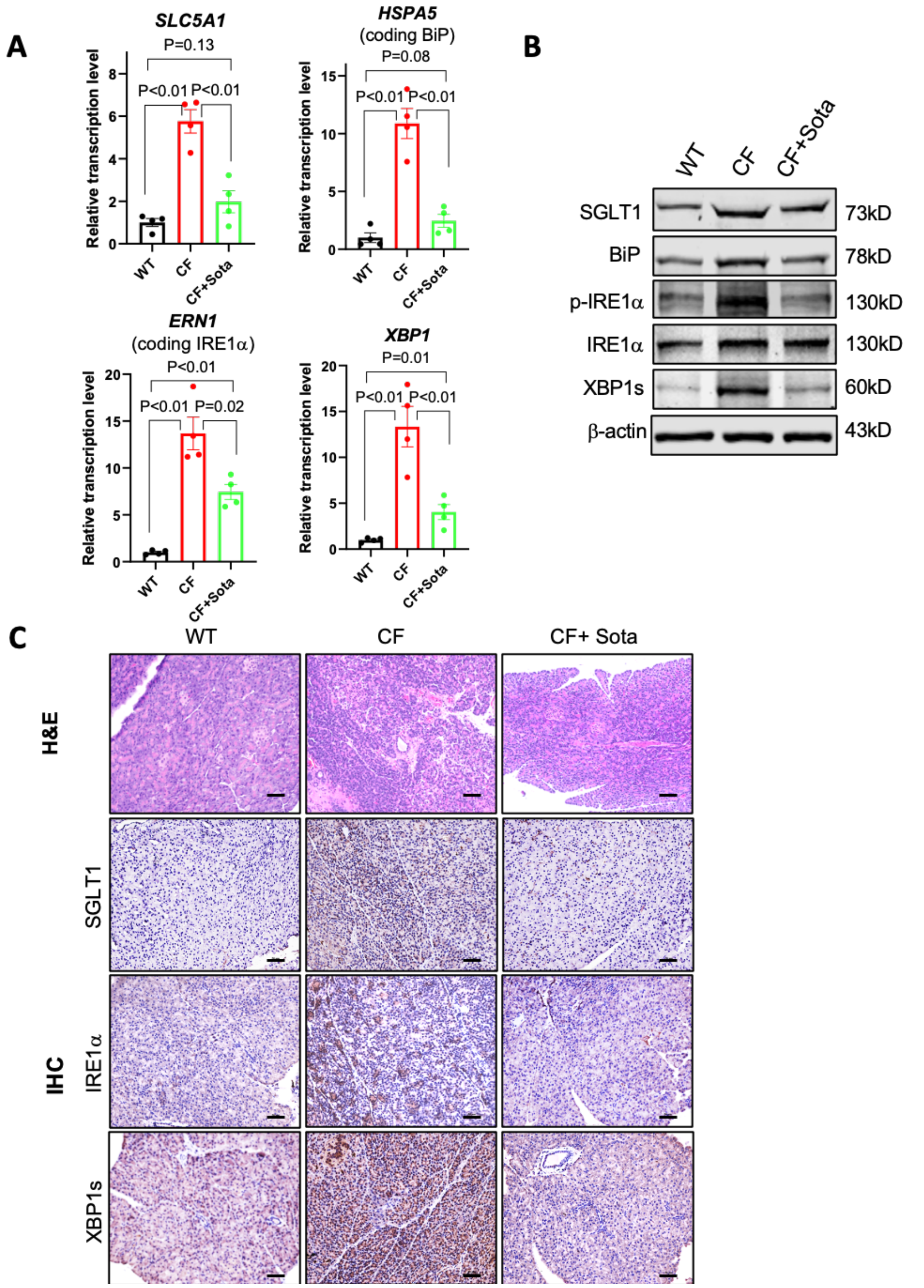

Supplementary Table 1. Selected SGLT1/2 inhibitors that have gained major regulatory agencies' approval (adapted from ref (*1*))

**Molecule**	**SGLT2 (IC50 nM)**	**SGLT1 (IC50 nM)**	**SGLT2 selectivity over SGLT1**	**FDA/EMA status/year**
Empagliflozin	3.1	8,300	~2,700-fold	FDA 2014; EMA 2014
Ertugliflozin	0.9	1,960	~2,200-fold	FDA 2017
Dapagliflozin	1.2	1,400	~1,200-fold	FDA 2014; EMA 2012
Canagliflozin	2.7	710	~260-fold	FDA 2013; EMA 2013
Sotagliflozin	1.8	36	~20-fold	FDA 2023; EMA 2019

Supplementary Table 2. Summary of blood chemistry parameters (mean ± SEM) of WT rabbits with or without Sotagliflozin treatment.

	**WT**	**WT+Sota**
**Na (mmol/L)**	139.67 ± 1.70	139.67 ± 2.62
**K (mmol/L)**	3.99 ± 0.15	4.18 ± 0.08
**Cl (mmol/L)**	105.67 ± 0.47	101.67 ± 0.94
**Cr (mg/dL)**	0.94 ± 0.03	0.89 ± 0.07
**BUN (mg/dL)**	15.67 ± 0.94	18.67 ± 4.03
**Trig (mg/dL)**	51.67 ± 6.85	54.00 ± 11.43
**Chol (mg/dL)**	59.00 ± 8.52	53.00 ± 3.74
**Gluc (mg/dL)**	116.67 ± 8.96	117.00 ± 10.61
**AST (U/L)**	9.33 ± 3.40	13.00 ± 3.74
**ALT (U/L)**	26.67 ± 0.47	32.33 ± 3.86
**ALP (U/L)**	83.00 ± 3.56	93.00 ± 3.56
**CPK (U/L)**	491.67 ± 55.82	571.67 ± 125.14
**TBIL (mg/dL)**	0.04 ± 0.01	0.05 ± 0.00
**ALB (g/dL)**	3.87 ± 0.17	3.87 ± 0.12
**TPRO (g/dL)**	5.63 ± 0.12	5.73 ± 0.17
**Ca (mg/dL)**	12.27 ± 0.82	12.30 ± 0.65

Supplementary Table 3. Animal information for the bile acid analysis.

**Sample type**	**Genotype**	**Number**	**Sota**	**Age** (mean ± SEM)
Bile	WT	5	N	132 ± 11 days
	CF	5	N	105 ± 20 days
	CF	4	Y	185 ± 35 days
Liver tissue	WT	5	N	132 ± 11 days
	CF	5	N	116 ± 18 days
	CF	6	Y	165 ± 29 days

Supplementary Table 4. Reagents and antibodies

**Reagents**			Cat#	Conditions
	Sotagliflozin (Sota)	Sun-Shine Chemical Technology Co., Ltd, Shanghai, China.		
	Glucose Assay kit	Abcam (UK)	ab65333	
	Lipase Assay kit	Abcam (UK)	ab102524	
	PAS Staining kit	Fisher Scientific	M1016460001	
	Columbia Agar	Becton Dickinson GmbH	221165	
**Primary Antibodies**				
	CFTR	Cystic Fibrosis Foundation Therapeutics (Bethesda, MD)	217<br>596	1:2500 western
	SGLT1	Abcam (UK)<br>Invitrogen (Waltham, Massachusetts)	ab97682,<br>ab14686,<br>PA5-88282	1:1000 western<br>1:100 IHC<br>1:100 IF
	SGLT2	Proteintech Group, Inc (Rosemont, IL)	24654-1-AP	1:100 IHC
	BiP/GRP78	Cell Signaling Technology (CST, Danvers, MA)	3177	1:1000 western
	p-IRE1a	ABclonal Technology (Woburn, MA)	AP0878	1:1000 western
	IRE1a	Cell Signaling Technology (CST, Danvers, MA)	3294	1:1000 western<br>1:100 IHC
	XBP1s	Cell Signaling Technology (CST, Danvers, MA)<br>BioLegend (San Diego, CA)	27901<br><br>619502	1:1000 western<br>1:100 IHC
	Albumin	Proteintech (Rosemont, IL)	66051-1-Ig	1:100 IF
	HRD1	Millipore Sigma (St. Louis, MO)	H7790	1:1000 Western
	Phospho-NF-kB p65/RelA-S536	Abclonal Technology (Woburn, MA)	AP0475	1:1000 Western
	Rabbit IgG Isotype Control	Abcam (UK)	ab172730	1:100 IHC
	GAPDH	Millipore Sigma (St. Louis, MO)	G8795	1:1000 Western

	β-actin	Cell Signaling Technology (CST, Danvers, MA)	3700	1:1000 western
**Secondary antibodies**	IRDye® 800CW Donkey anti-Rabbit	LI-COR Biosciences	D01216-10	1:3000 western
	IRDye® 680RD Donkey anti-Mouse	LI-COR Biosciences	D00226-05	1:3000 western
	Alexa Fluor® 488 AffiniPure Goat Anti-Rabbit IgG (H+L)	Jackson ImmunoResearch Laboratories	147158	1:500 IF
	Alexa Fluor® 594 AffiniPure Goat Anti-Mouse IgG (H+L)	Jackson ImmunoResearch Laboratories	151791	1:500 IF

Supplementary Table 5. qPCR primer sequences

	Gene name	Forwards Sequence 5'-3'	Reverse Sequence 5'-3'
**Human**	*SLC5A1*	TCCTCACCAAACCCATTCCG	TCCGCATCCAGGTCAATACG
	GAPDH	TGAAGGTCGGAGTCAACGG	AGAGTTAAAAGCAGCCCTGGTG
**Rabbit**	*SLC5A1*	ACTGGGTTCGCTTTTCACGA	CTGGGAATGGCTCGCATGTA
	SLC5A2	CTGGTTTTCAGTCTCCGGCA	TGGGGCTCTTCCATCTCCACT
	HSPA5	TGGGTGGTGGAACCTTTGAT	TGACACGCTGGTCGAAGTC
	ERN1	ATTGTGTACCGGGGCATGTT	CTCGTCTGATTCTCGCAGCA
	XBP1	GGGGATGGATGCCATGGTTA	GCTGCAGATGCACGTAGTCT
	ATF4	CCGGATGAGTCGTTCAGCTT	CTGTCCTCCTCCTTGACGC
	HSP90B1	AGTACGGATGGTCTGCCAAC	TCGAACGTCTTTTGACTGGCA
	TNF	CTGCACTTCAGGGTGATCG	CTACGTGGGCTAGAGGCTTG
	LTA	CAGTGGAAAGACCCCACATCTC	GACGCAGGCAGCAATTATCC
	IL1B	TTGAAGAAGAACCCGTCCTCTG	CTCATACGTGCCAGACAACACC
	IL6	CTACCGCTTTCCCCACTTCAG	TCCTCAGCTCCTTGATGGTCTC
	GAPDH	CCGAGACACGATGGTGAAGG	TGATGGCGACAACATCCACT

References in the Supplementary data

1. Q. Zeng *et al.*, Mechanisms and Perspectives of Sodium-Glucose Co-transporter 2 Inhibitors in Heart Failure. *Front Cardiovasc Med* **8**, 636152 (2021).
