# Supplementary material for: Sotagliflozin attenuates liver-associated disorders in cystic fibrosis rabbits
Source: JCI Insight. 2024 Feb 15;9(6):e165826. doi: 10.1172/jci.insight.165826 (PMC10972622; doi:10.1172/jci.insight.165826)

Full unedited gel for Figure 1A

SGLT1

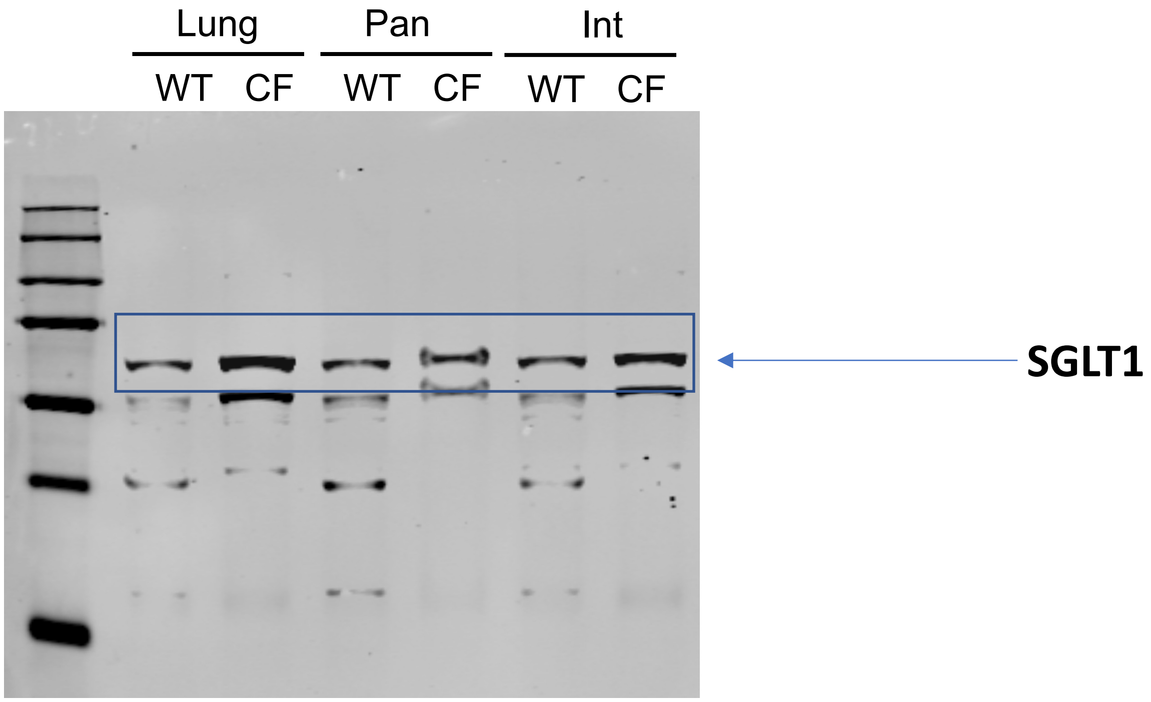

Full unedited gel for Figure 1A

β-actin

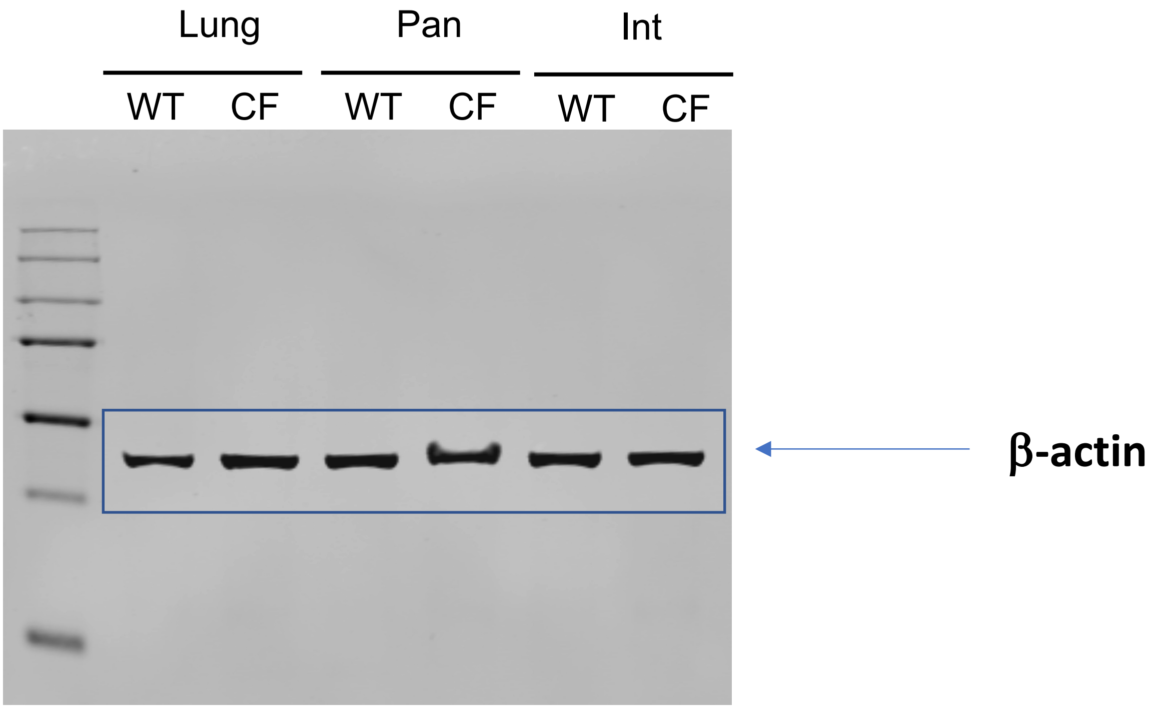

Full unedited gel for Figure 1A

CFTR

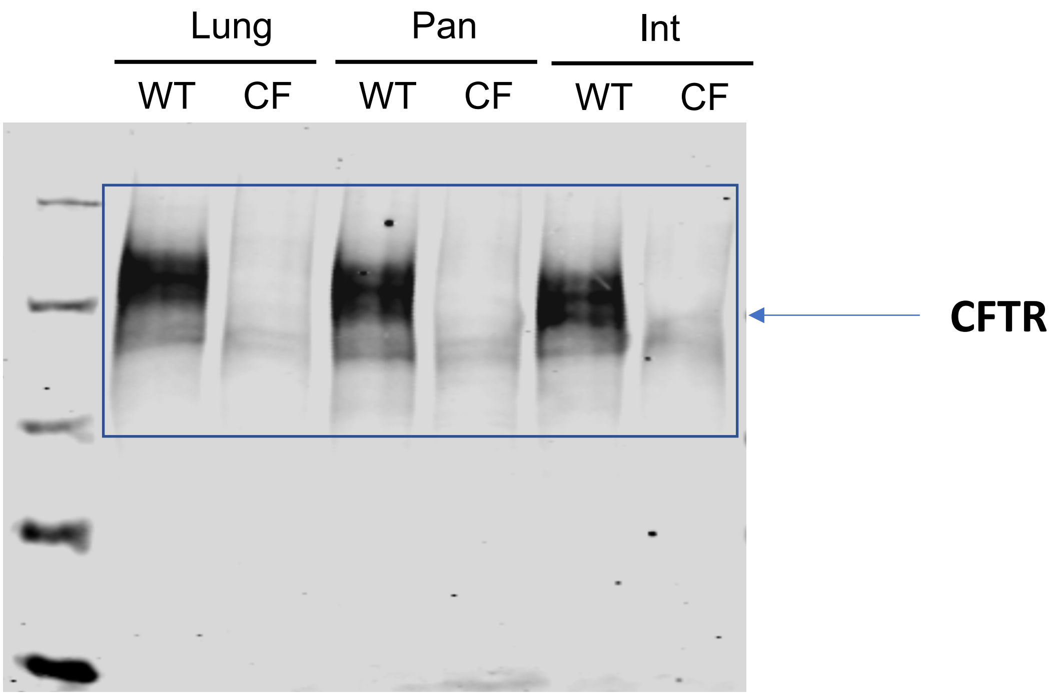

Full unedited gel for Figure 1D

SGLT1

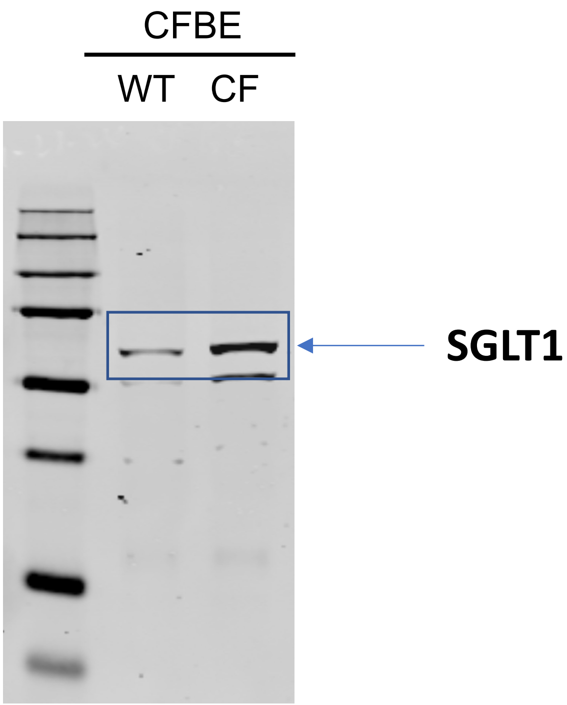

Full unedited gel for Figure 1D

β-actin

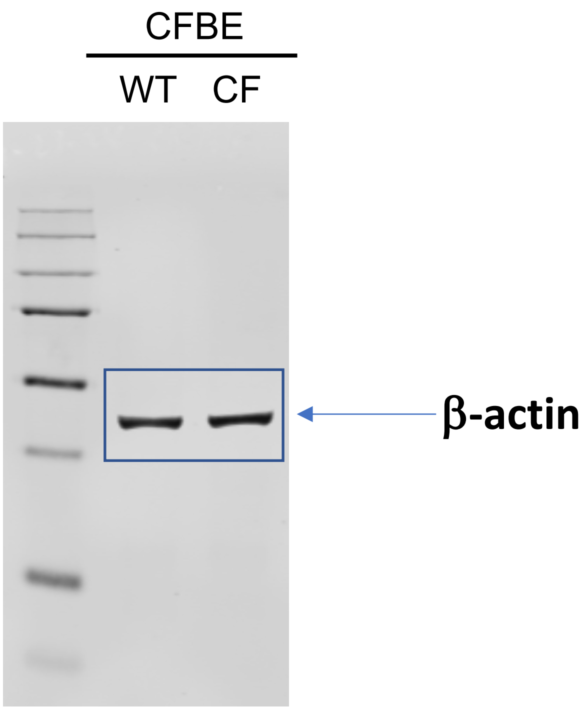

Full unedited gel for Figure 1D

CFTR

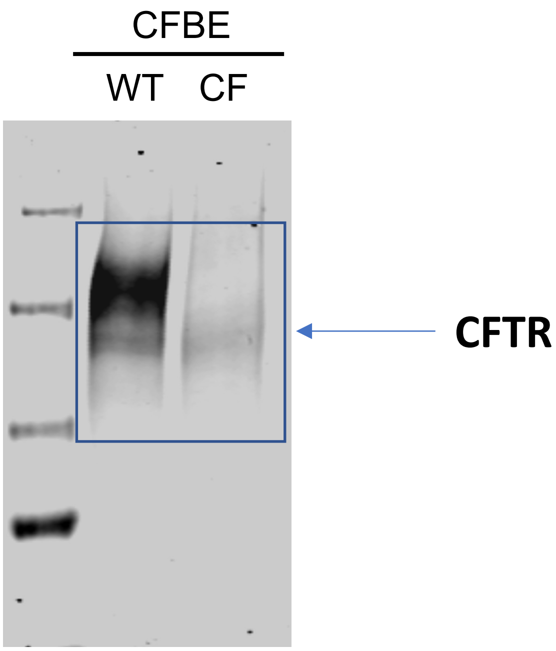

Full unedited gel for Figure 1E

SGLT1

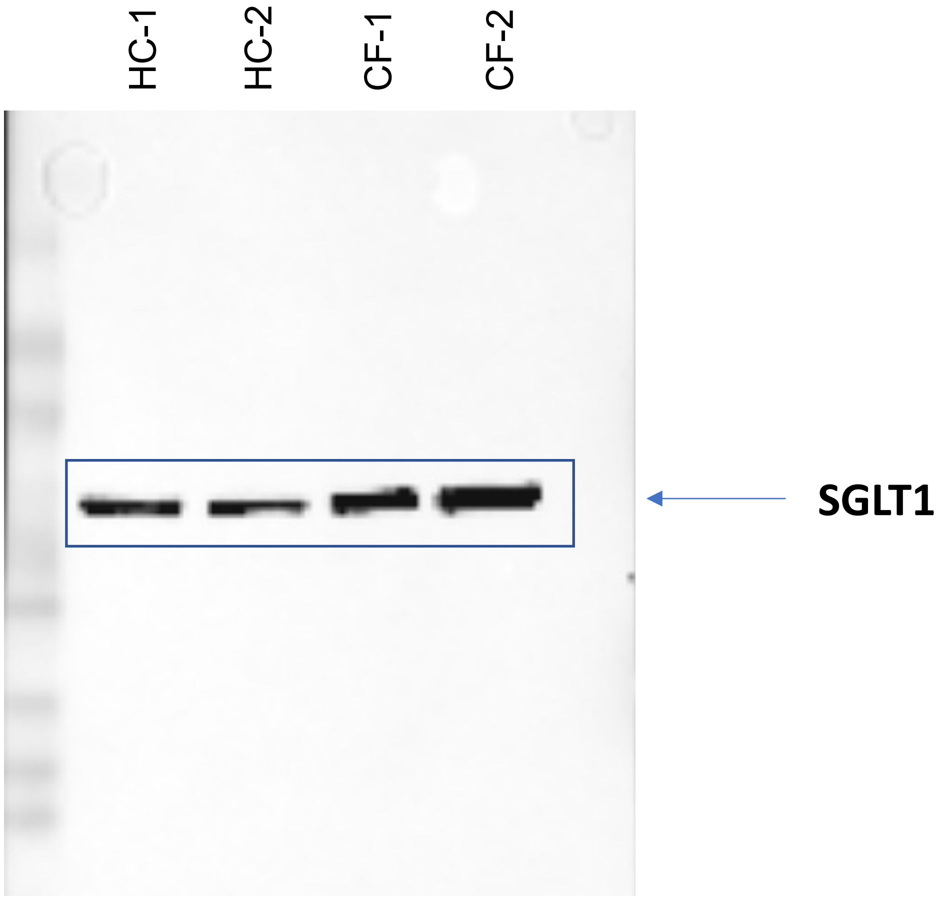

Full unedited gel for Figure 1E

β-actin

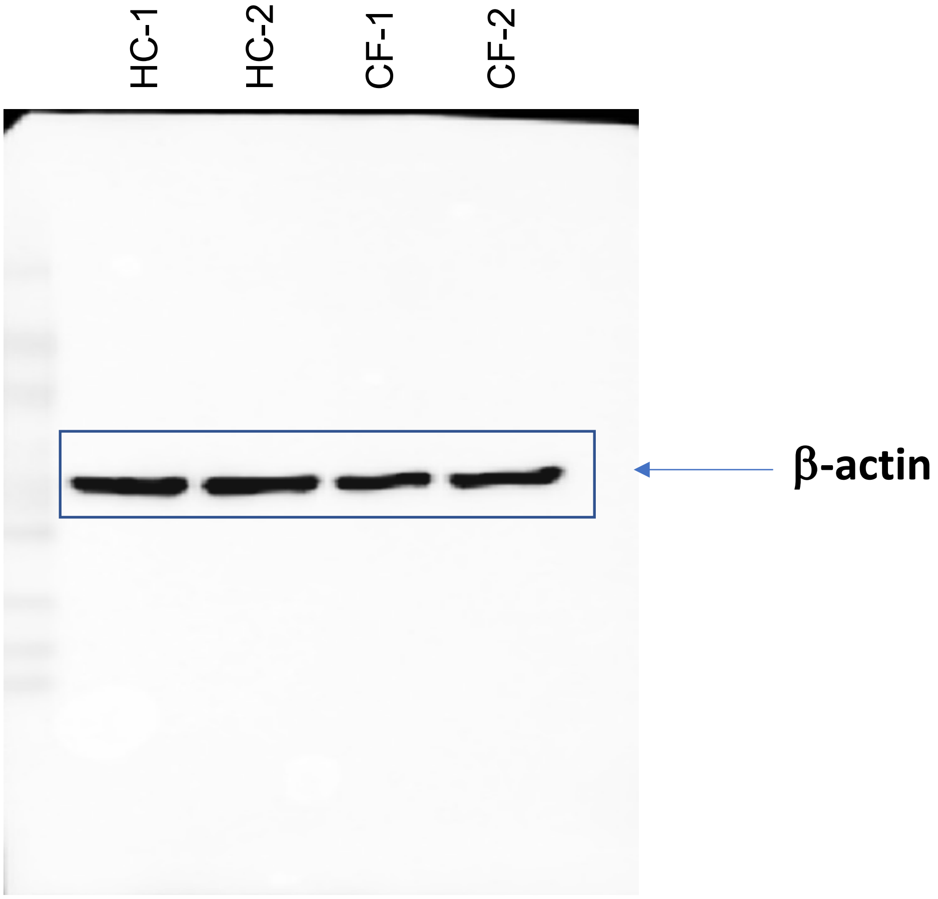

Full unedited gel for Figure 1E

CFTR

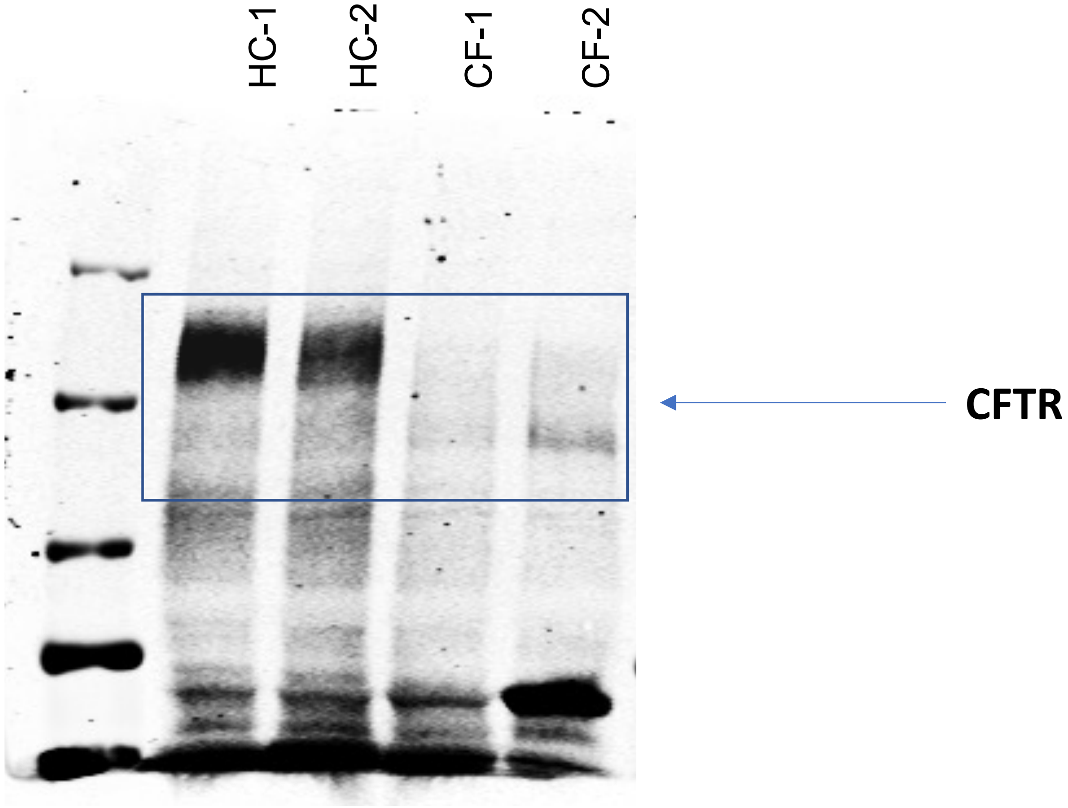

Full unedited gel for Figure 4A

SGLT1

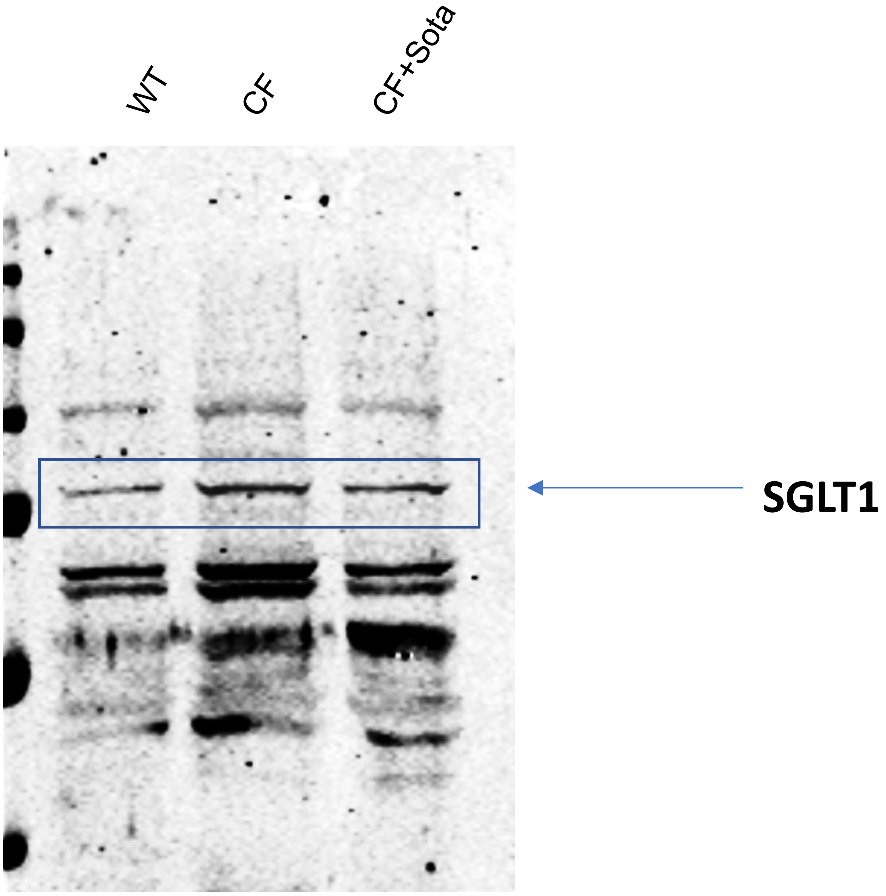

Full unedited gel for Figure 4A

β-actin

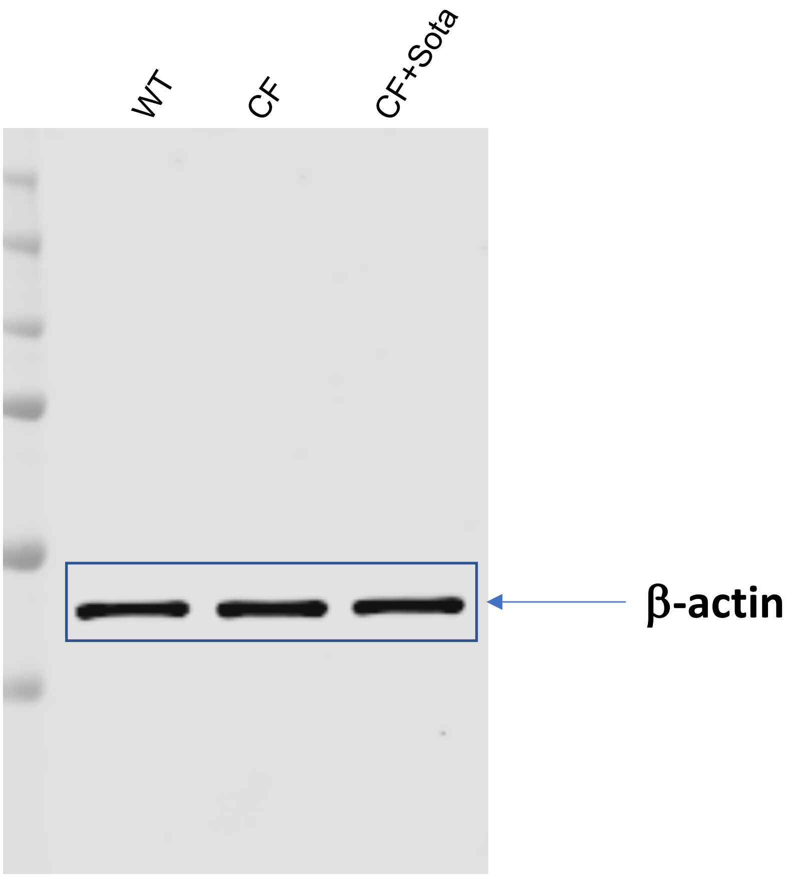

Full unedited gel for Figure 7B

GRP78

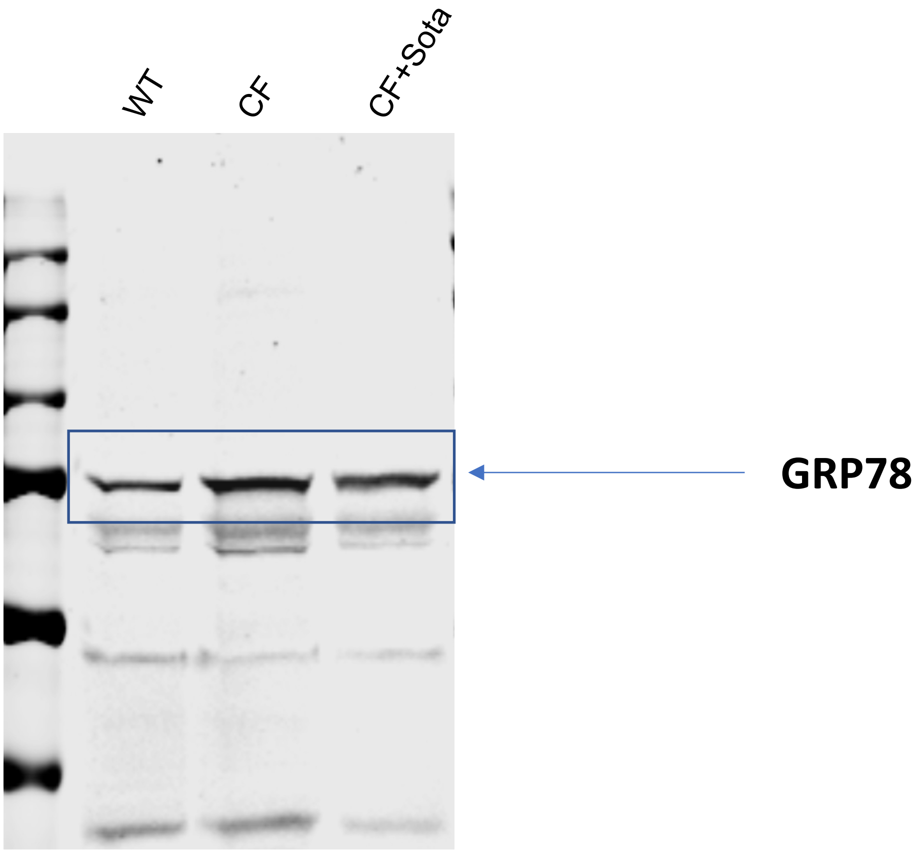

Full unedited gel for Figure 7B

p-IRE1α

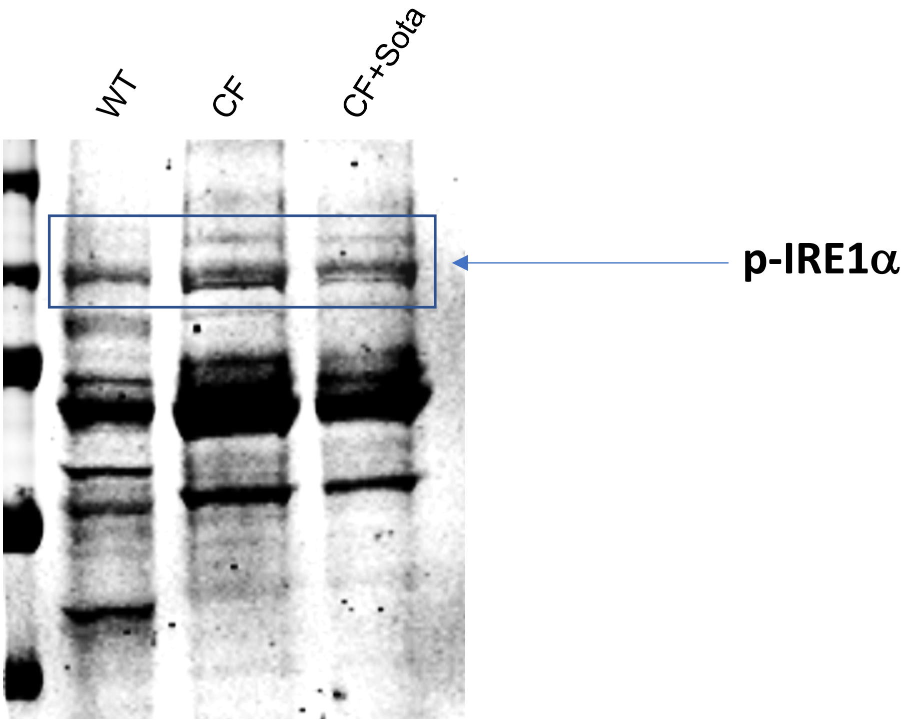

Full unedited gel for Figure 7B

IRE1α

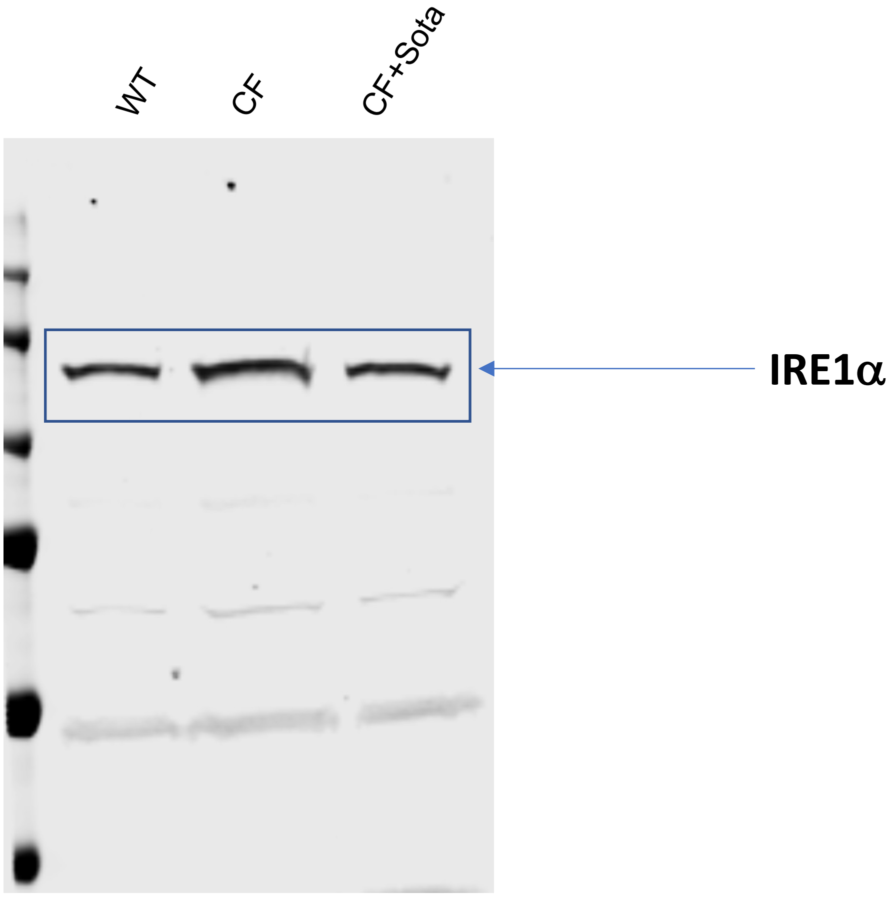

Full unedited gel for Figure 7B

XBP1s

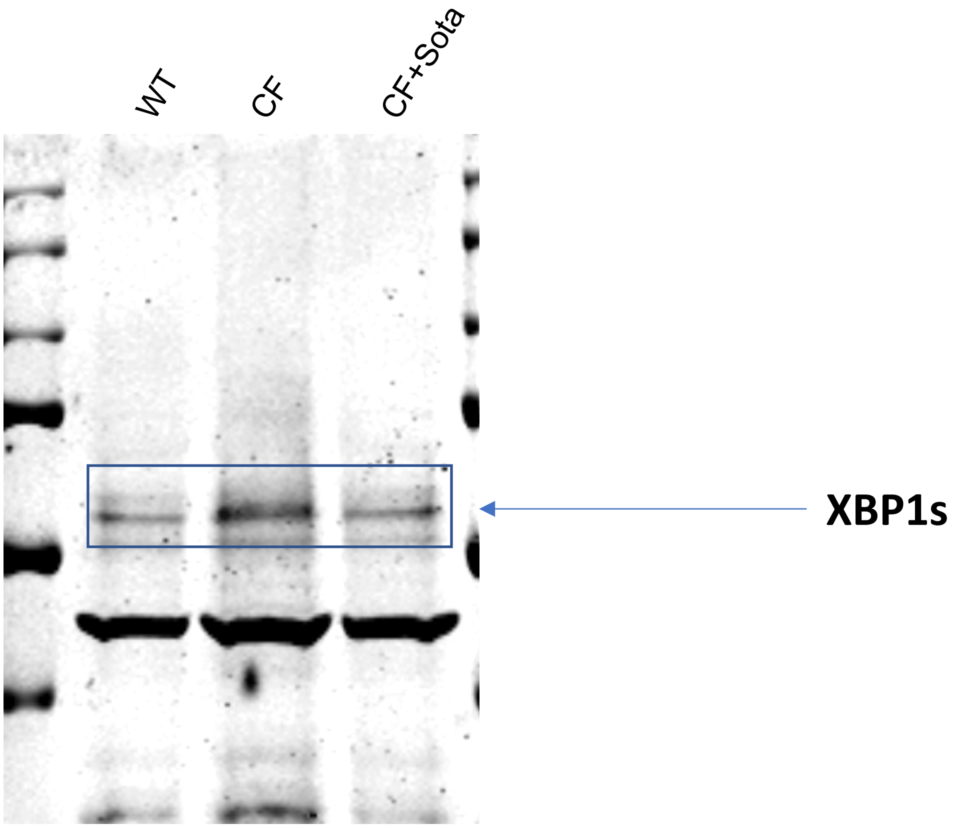

Full unedited gel for Figure 7B

Phos-P65 (NFκB)

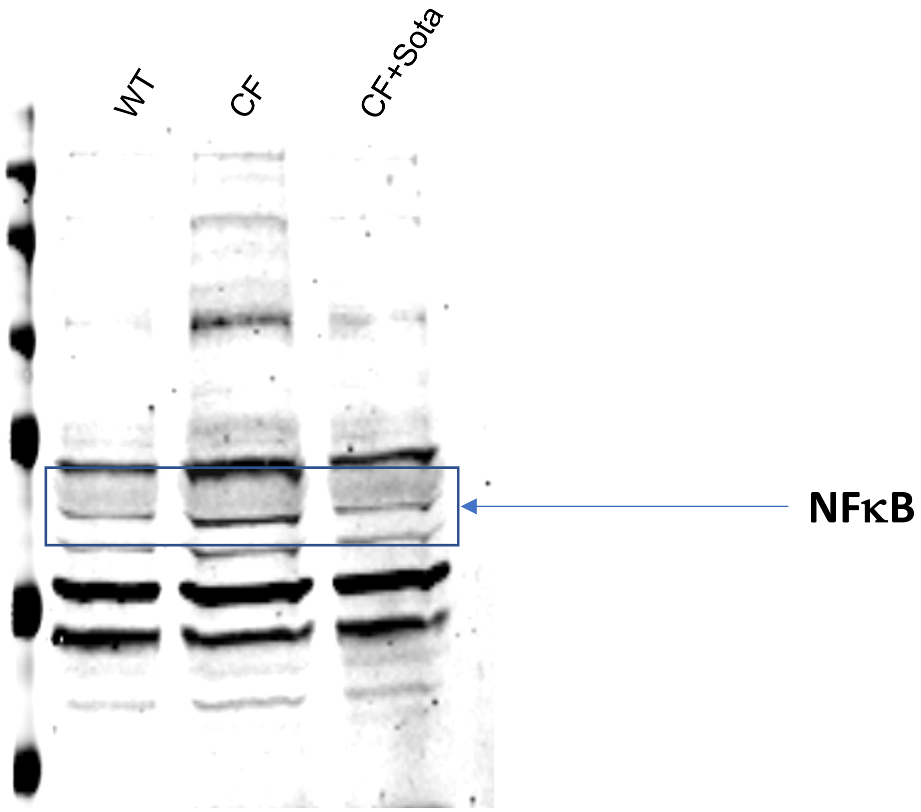

Full unedited gel for Figure 7B

β-actin

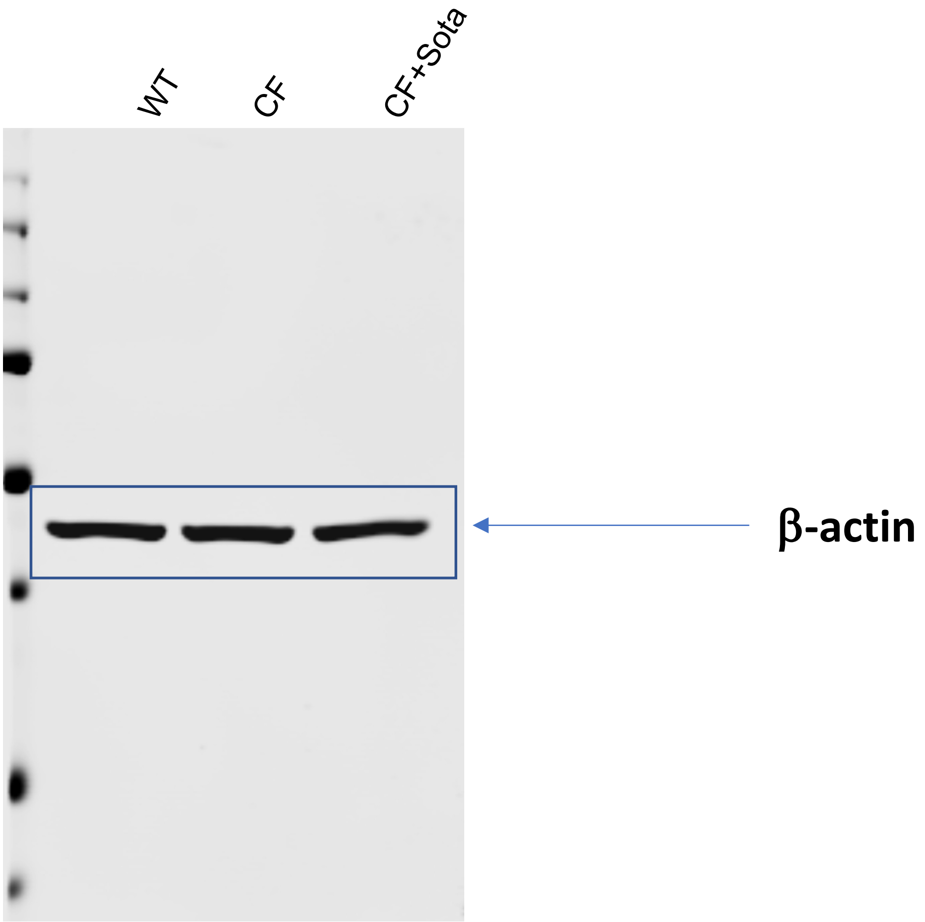

Full unedited gel for Supplementary Figure 1A

SGLT1

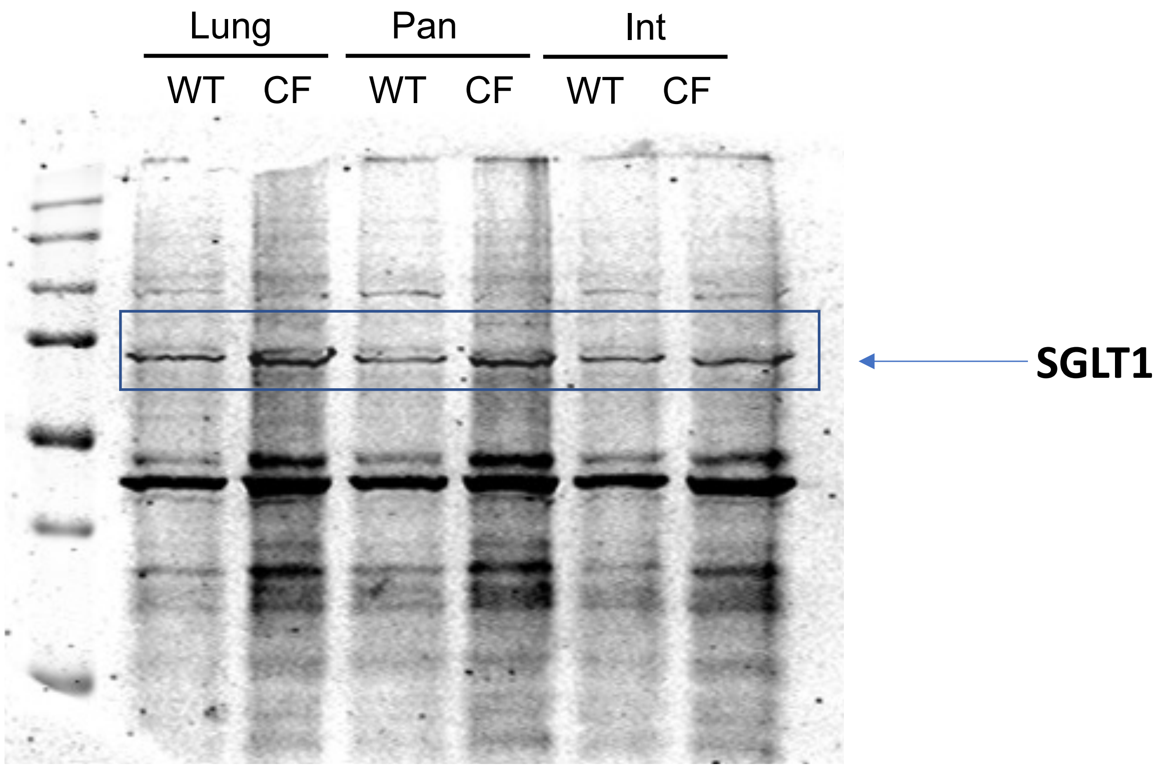

Full unedited gel for Supplementary Figure 1A

β-actin

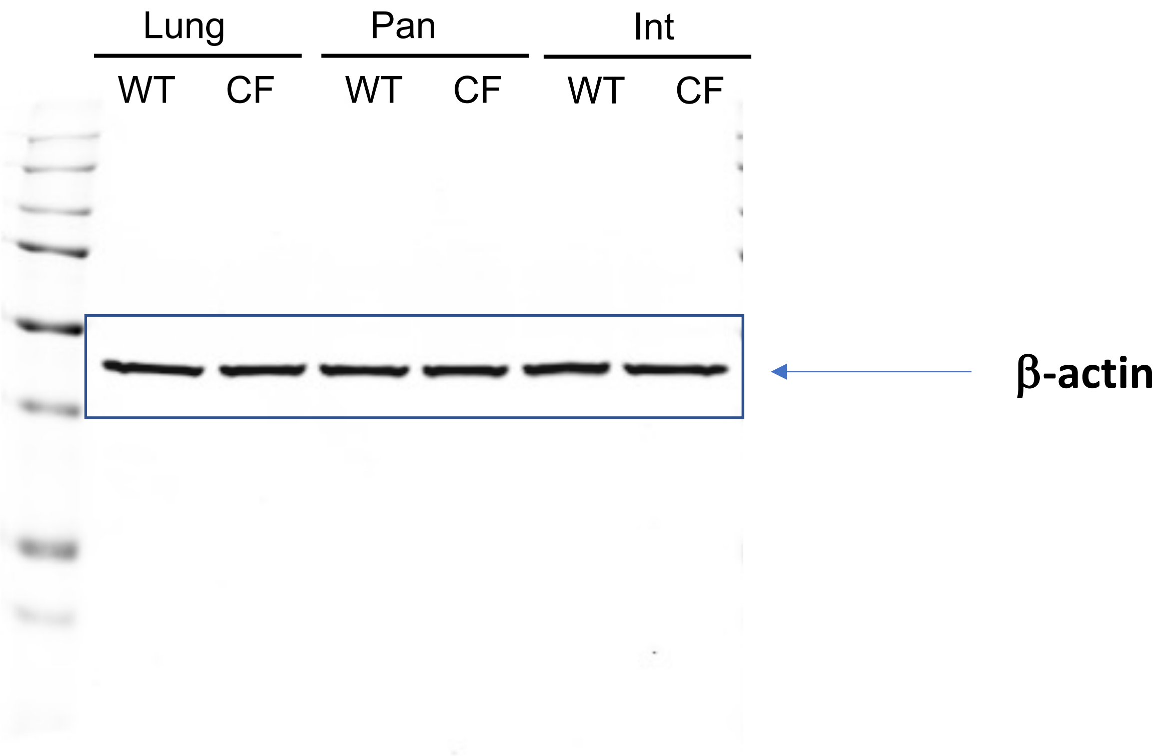

Full unedited gel for Supplementary Figure 1A

CFTR

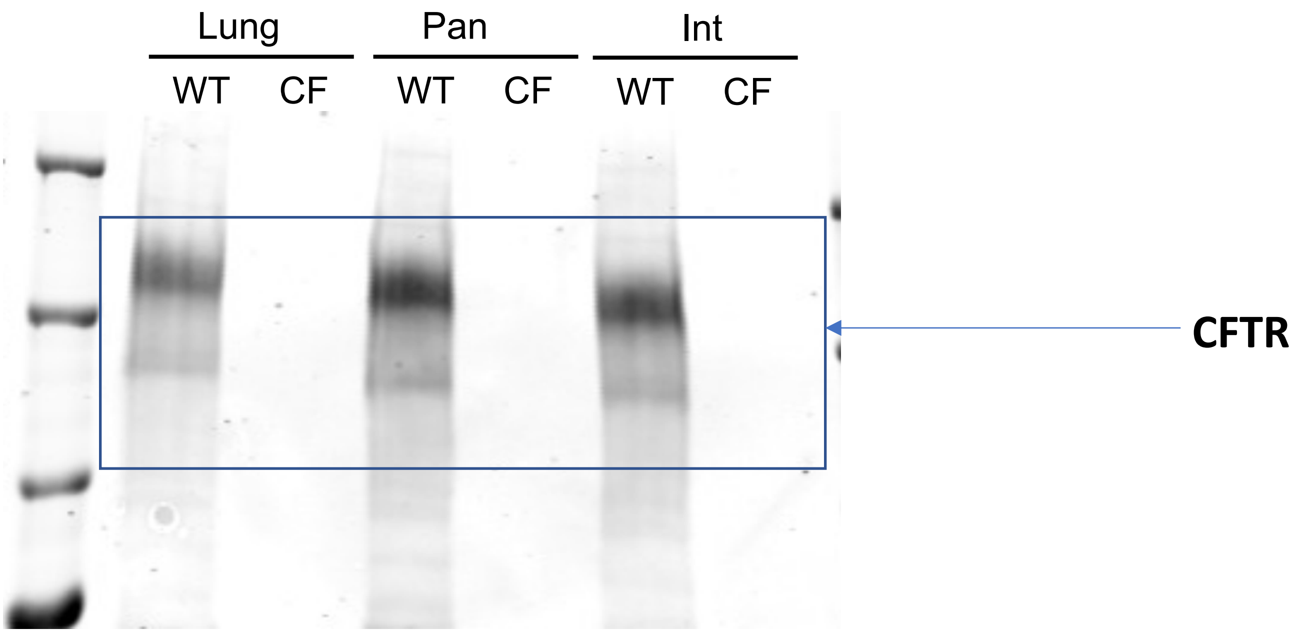

Full unedited gel for Supplementary Figure 1B

SGLT1

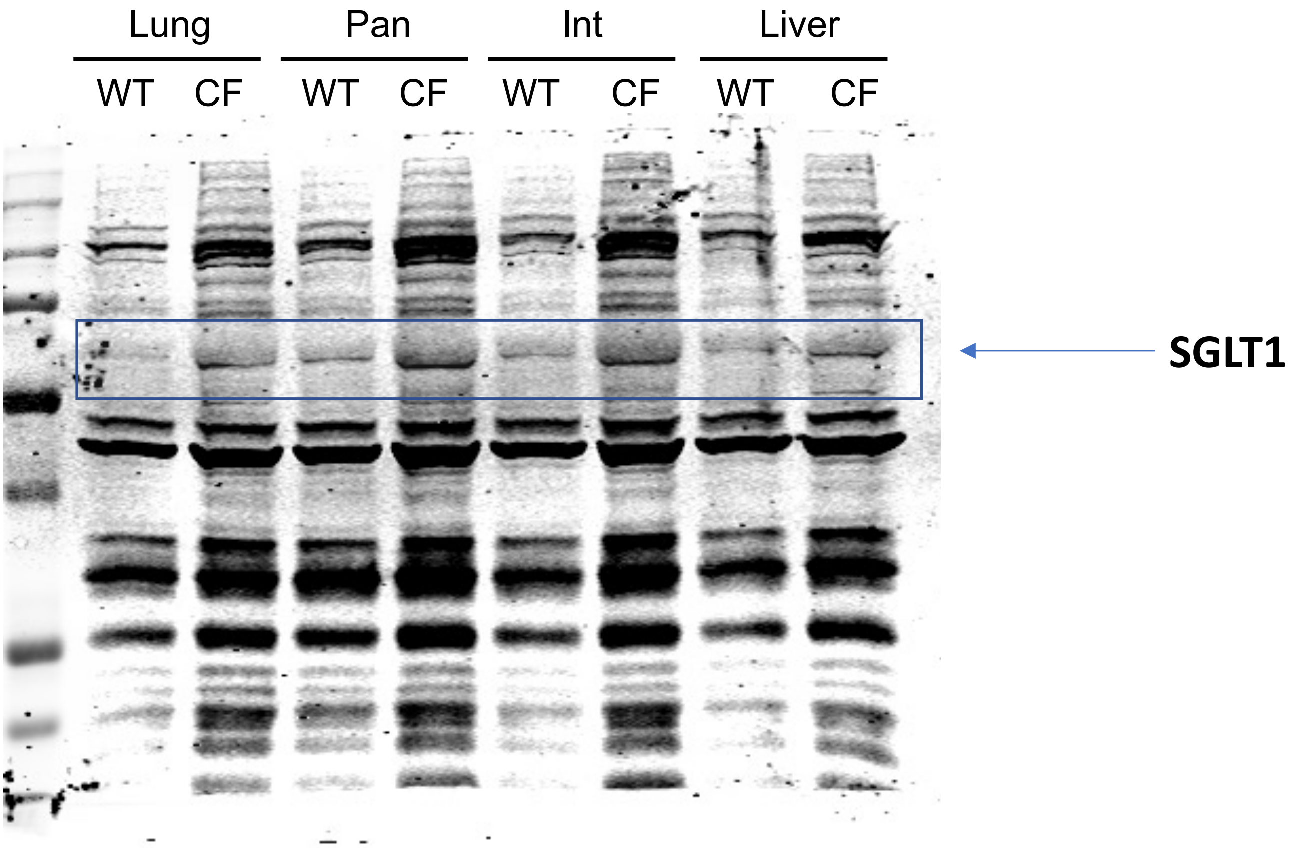

Full unedited gel for Supplementary Figure 1B

β-actin

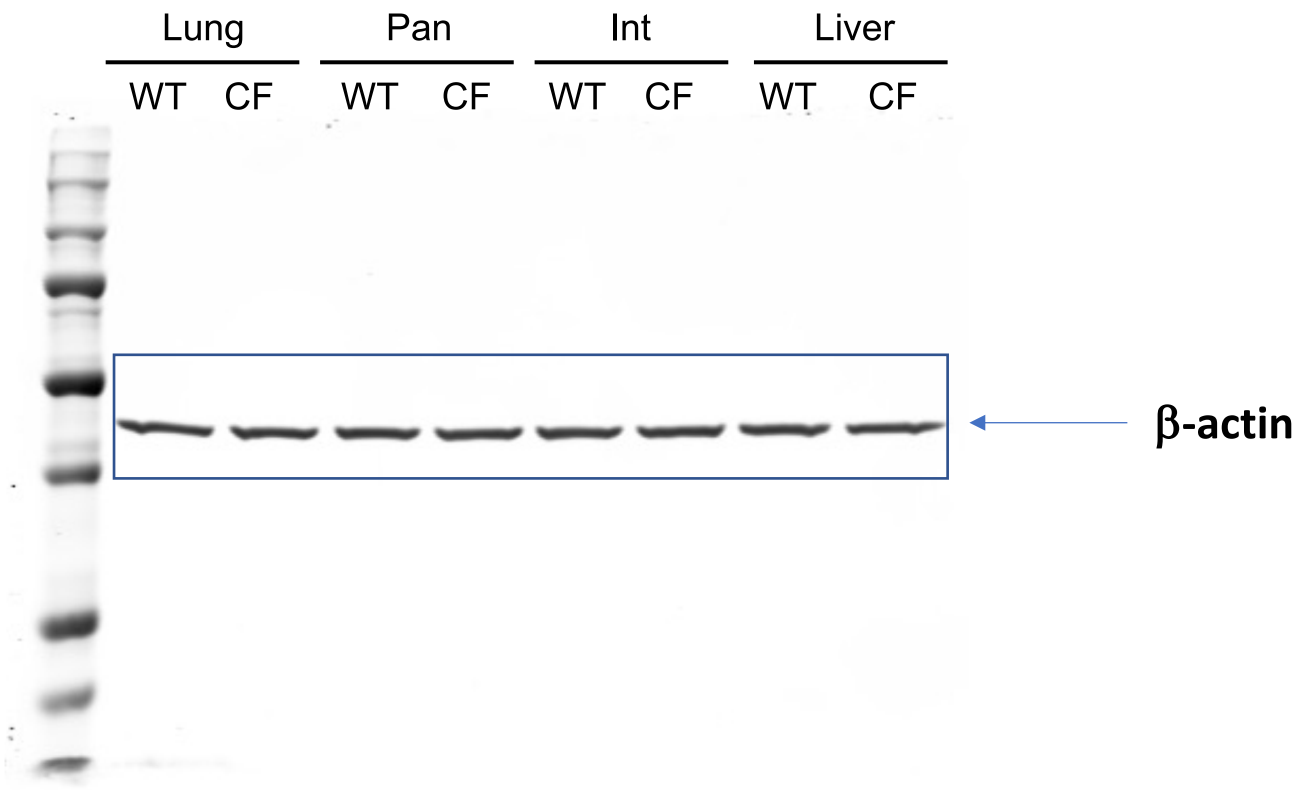

Full unedited gel for Supplementary Figure 1B

CFTR

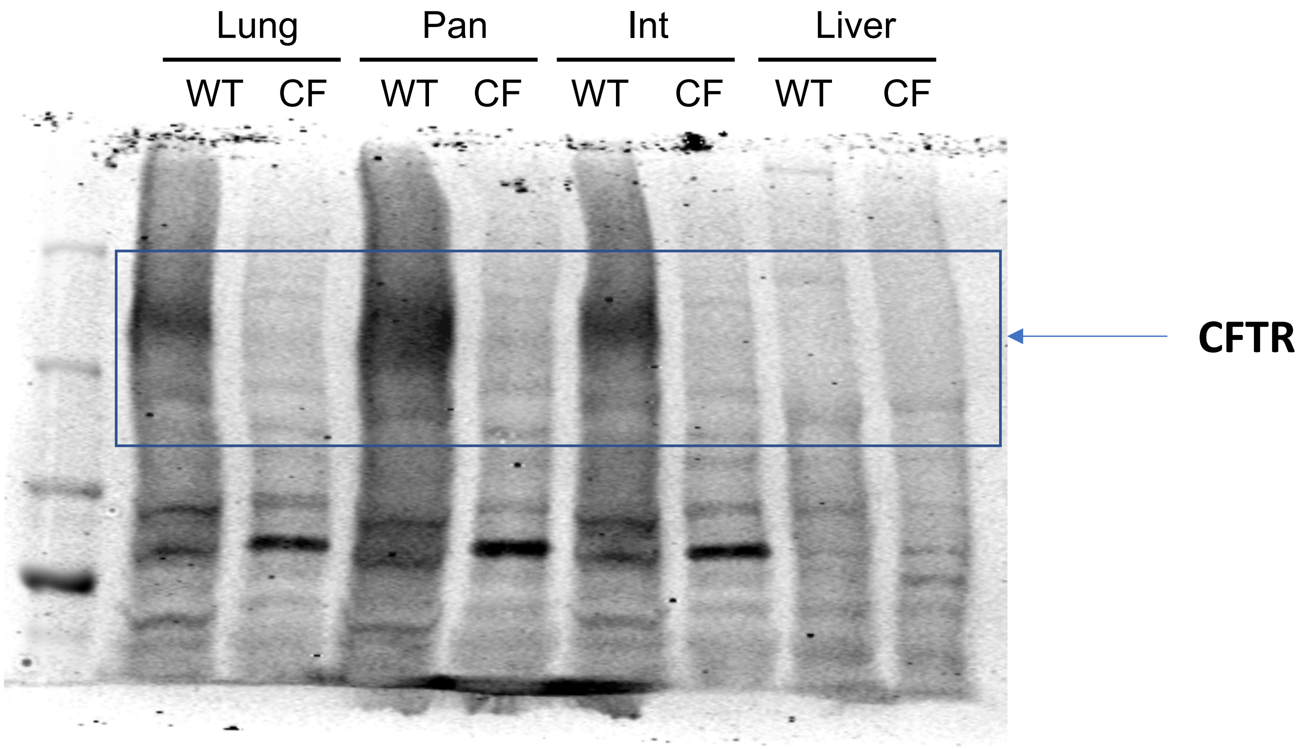

Full unedited gel for Supplementary Figure 7A

HRD1

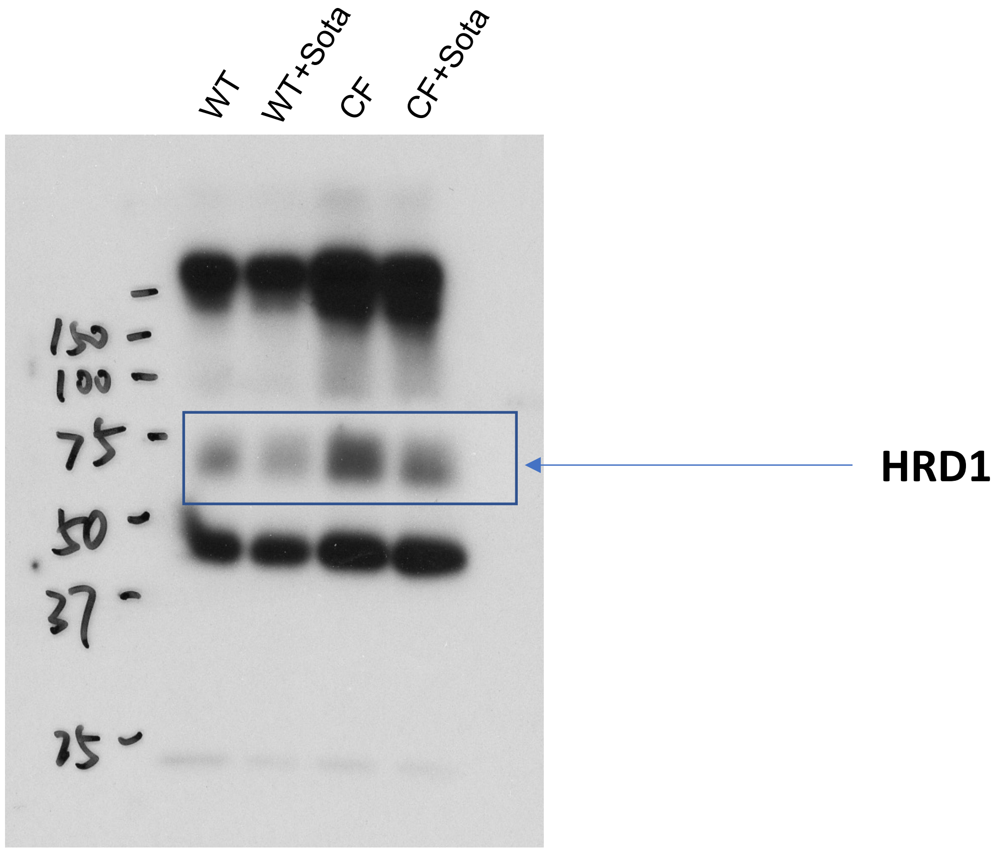

Full unedited gel for Supplementary Figure 7A

GAPDH

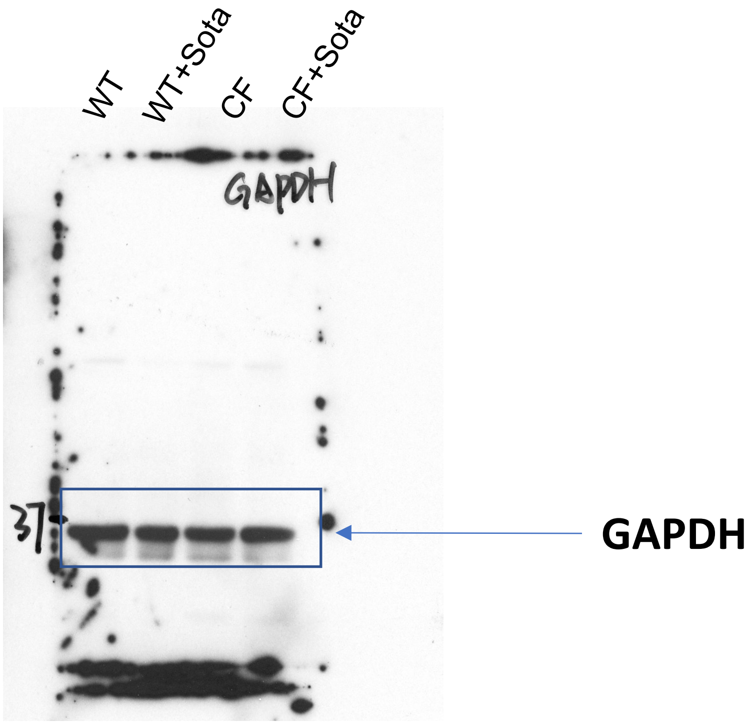

Full unedited gel for Supplementary Figure 9B

SGLT1

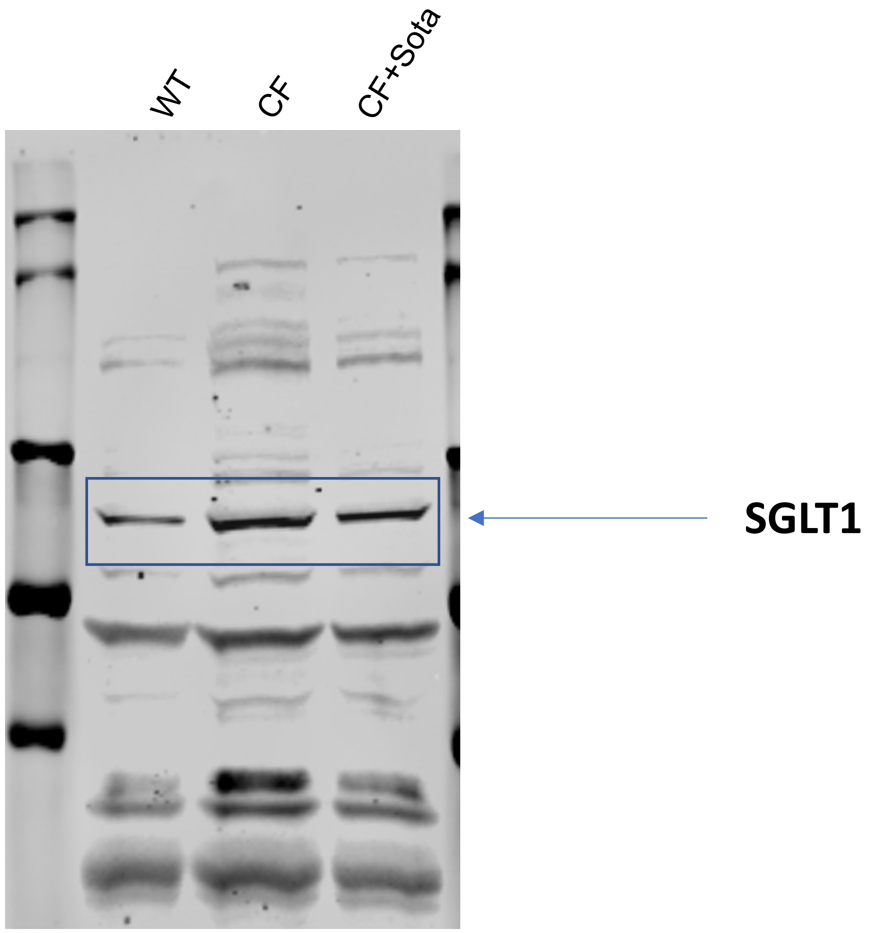

Full unedited gel for Supplementary Figure 9B

GRP78

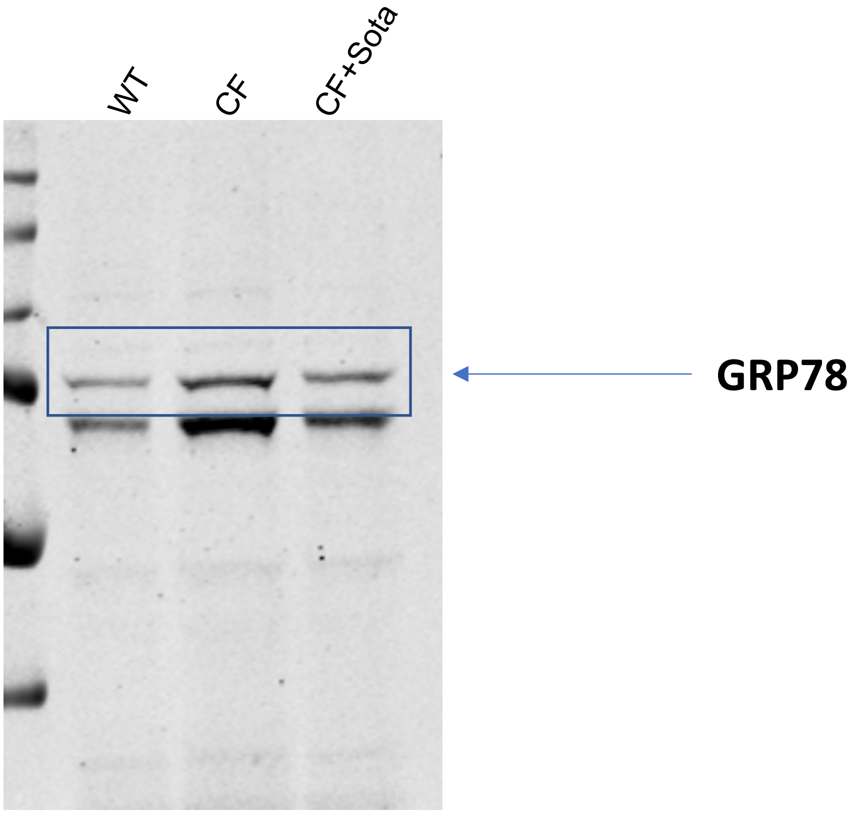

Full unedited gel for Supplementary Figure 9B

p-IRE1α

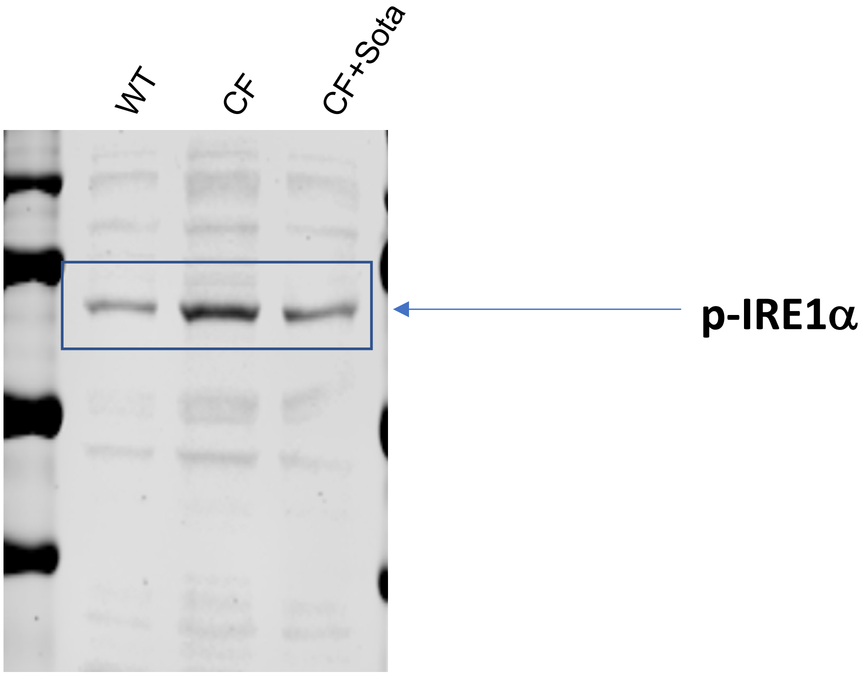

Full unedited gel for Supplementary Figure 9B

IRE1α

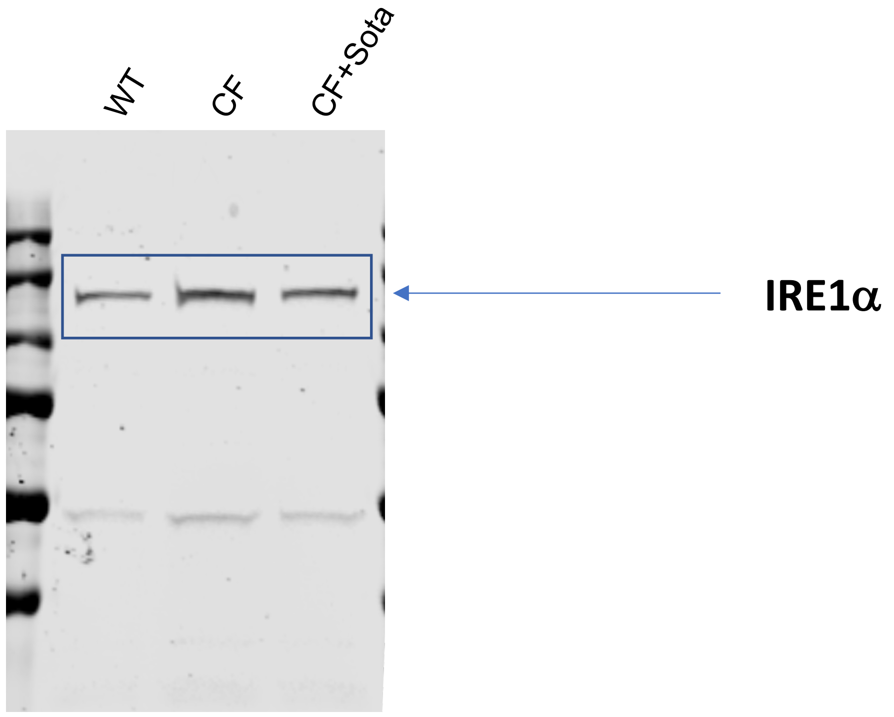

Full unedited gel for Supplementary Figure 9B

XBP1s

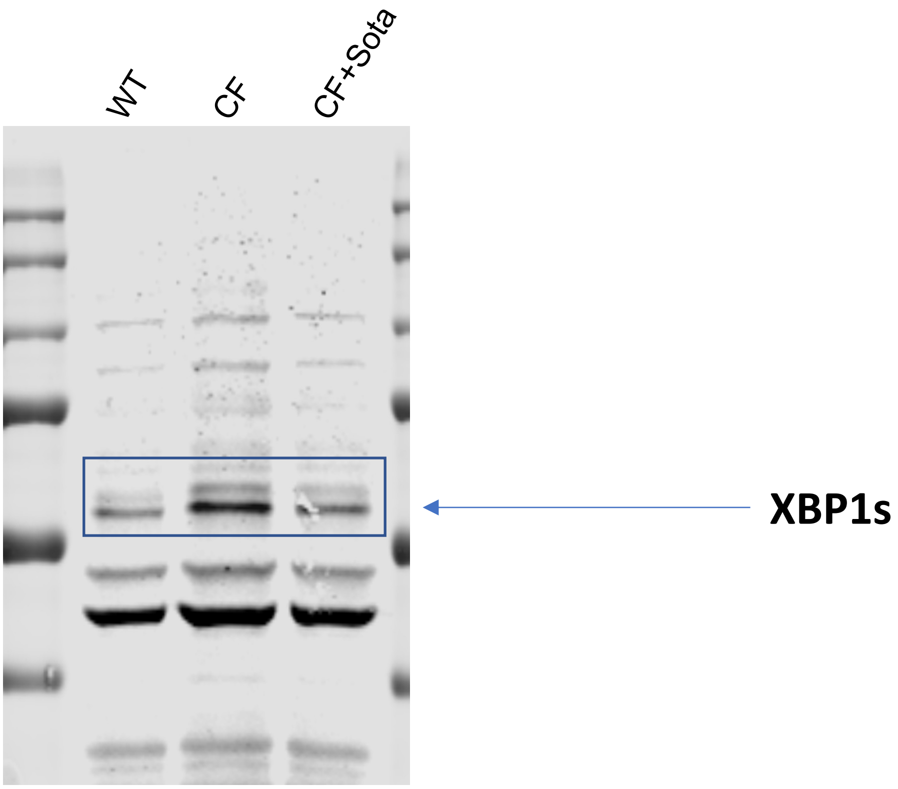

Full unedited gel for Supplementary Figure 9B

β-actin

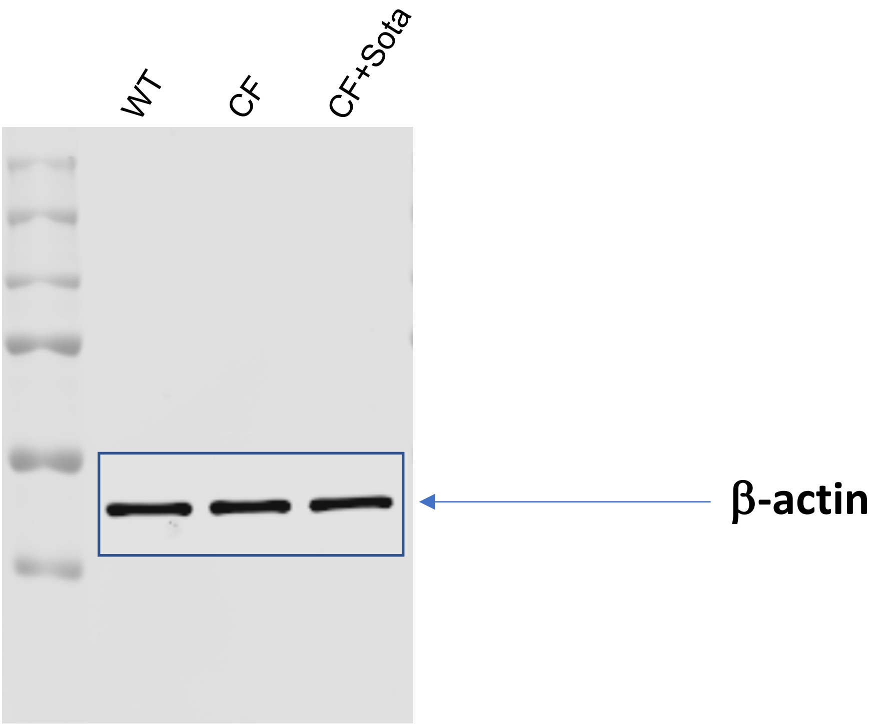

Full unedited gel for Supplementary Figure 10B

SGLT1

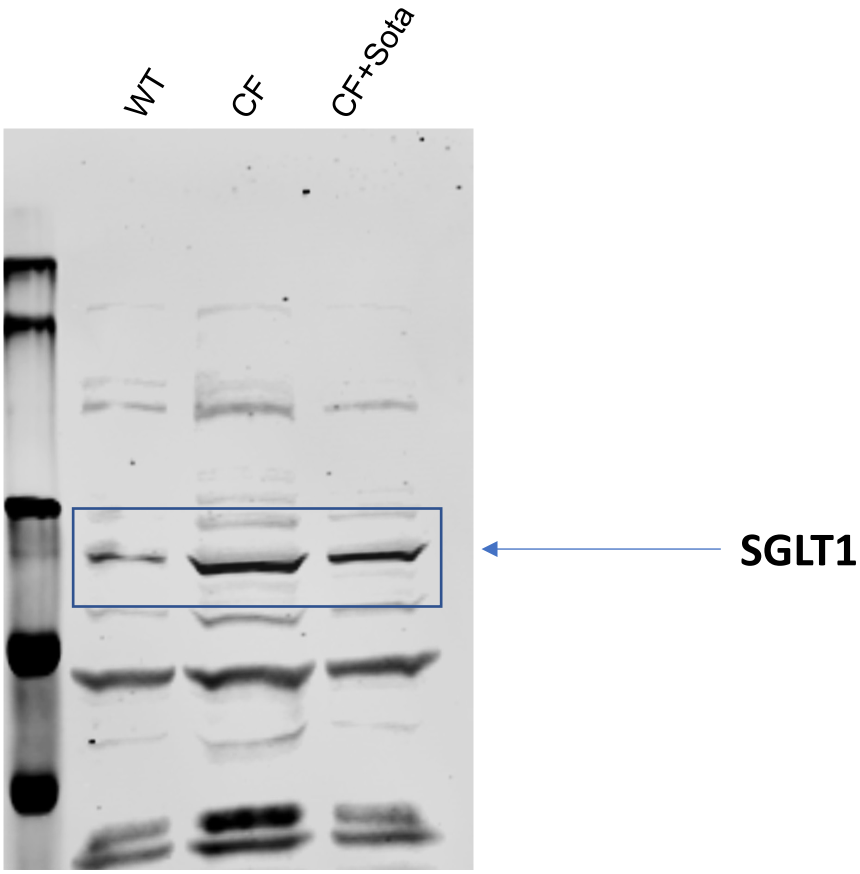

Full unedited gel for Supplementary Figure 10B

GRP78

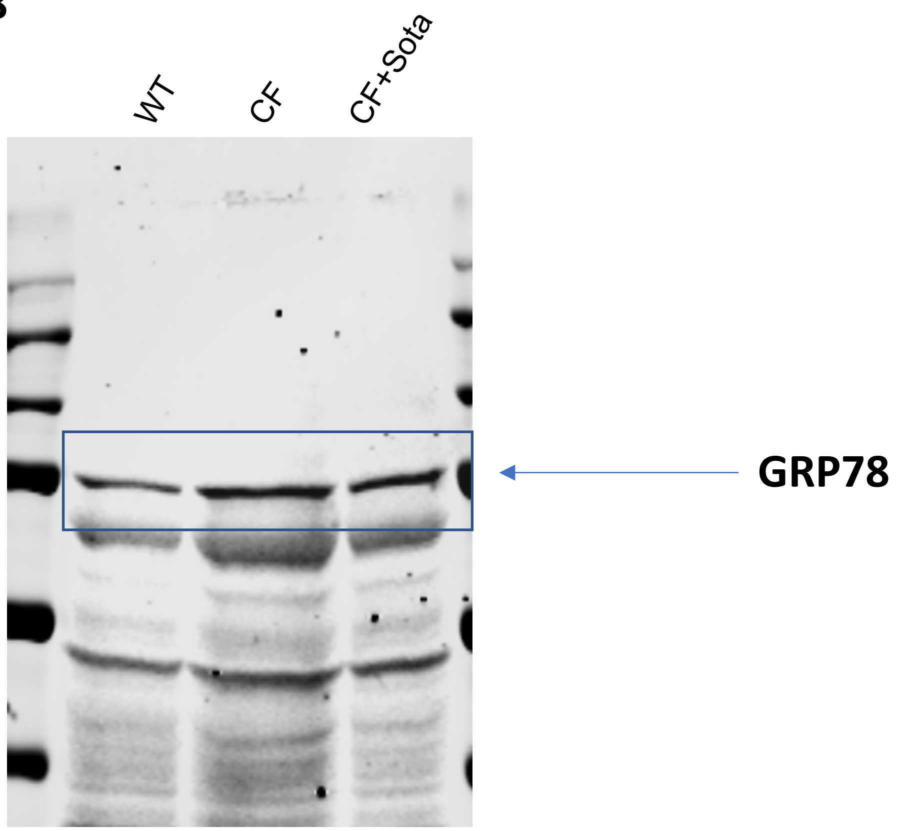

Full unedited gel for Supplementary Figure 10B

p-IRE1α

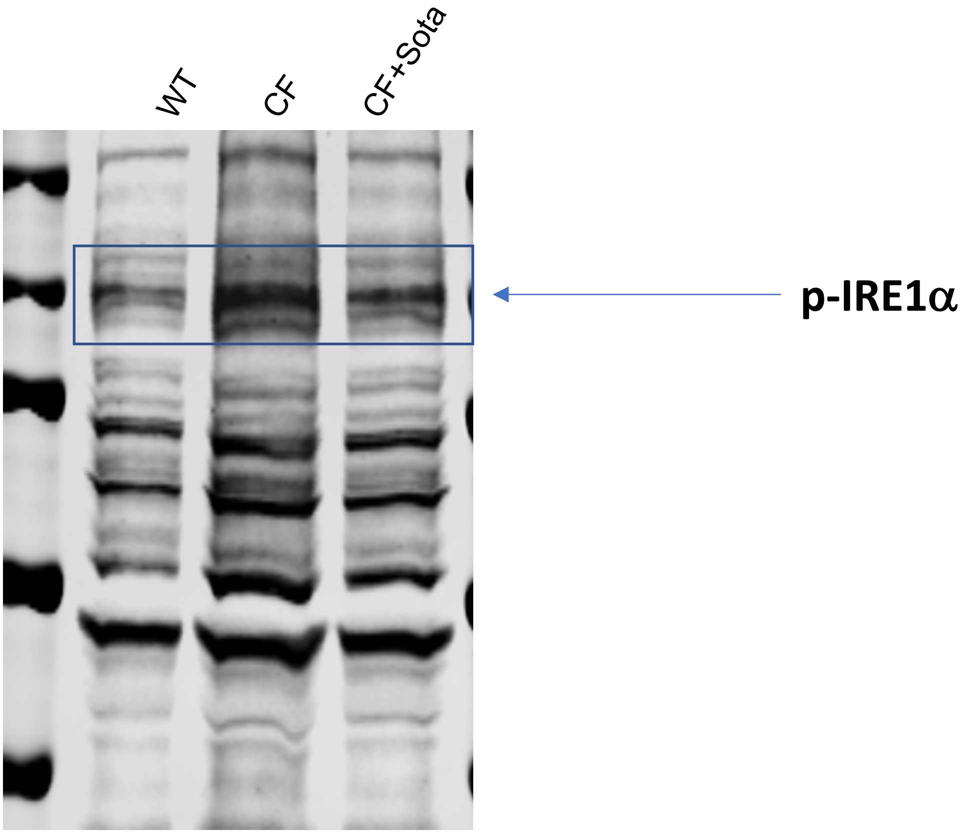

Full unedited gel for Supplementary Figure 10B

IRE1α

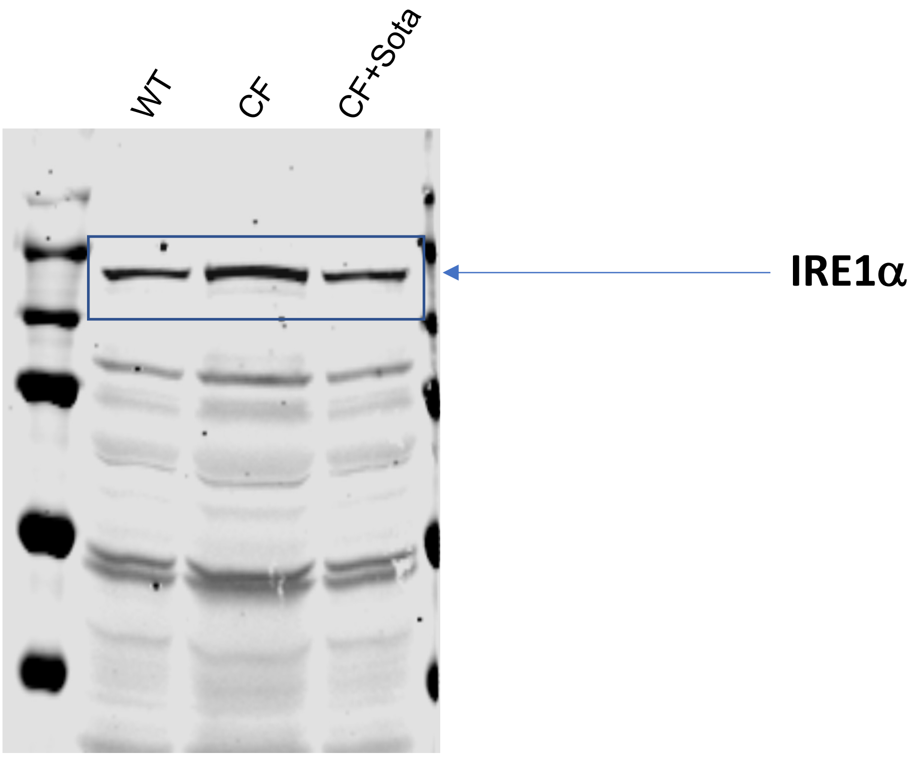

Full unedited gel for Supplementary Figure 10B

XBP1s

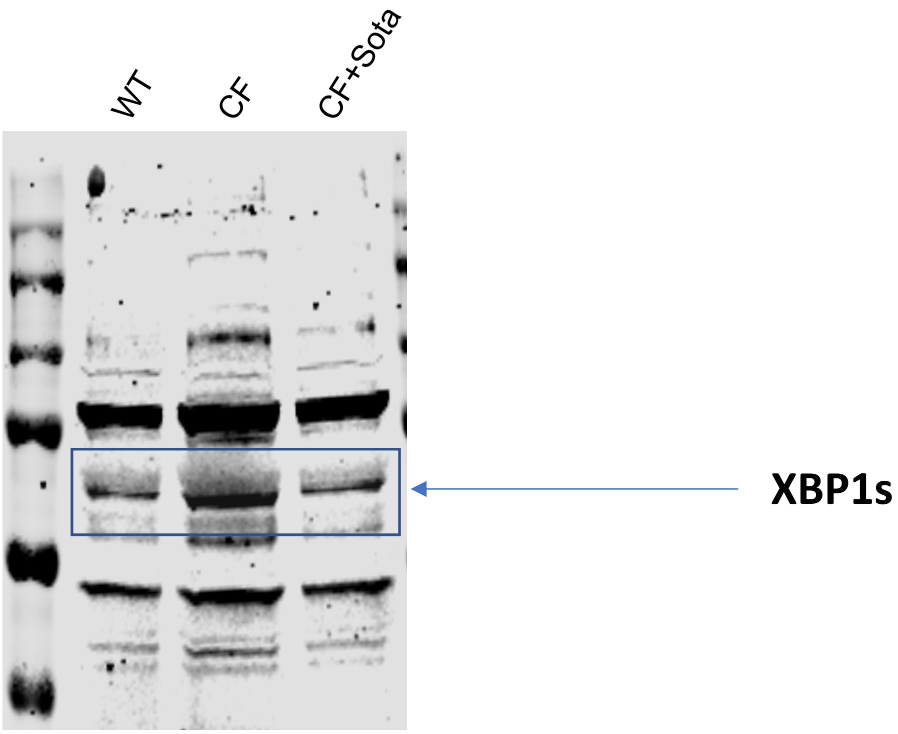

Full unedited gel for Supplementary Figure 10B

β-actin

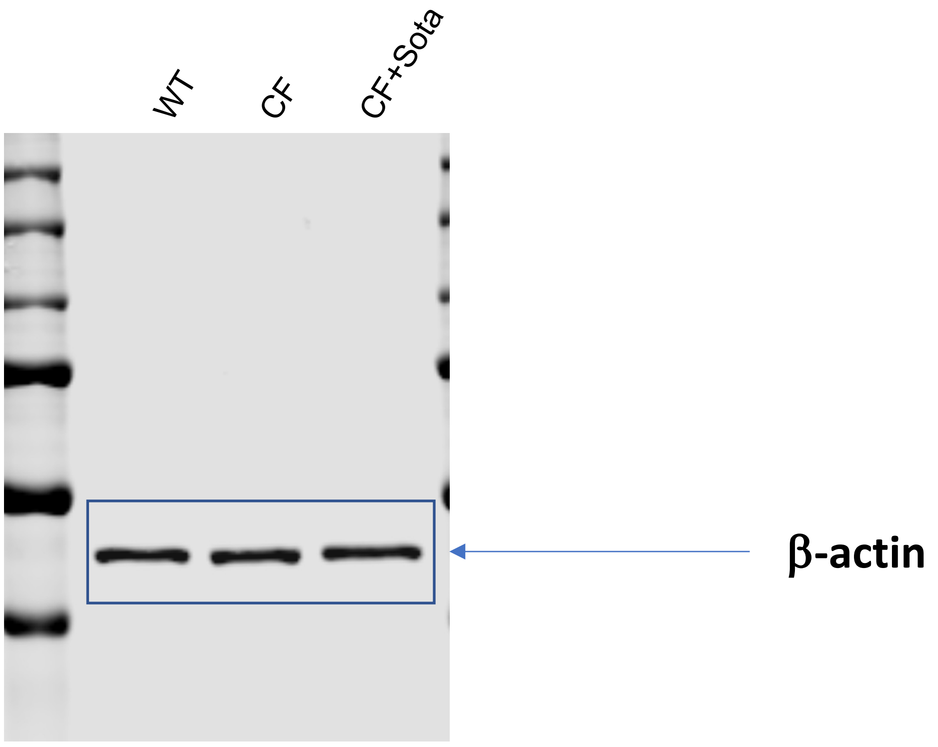

Full unedited gel for Supplementary Figure 11B

SGLT1

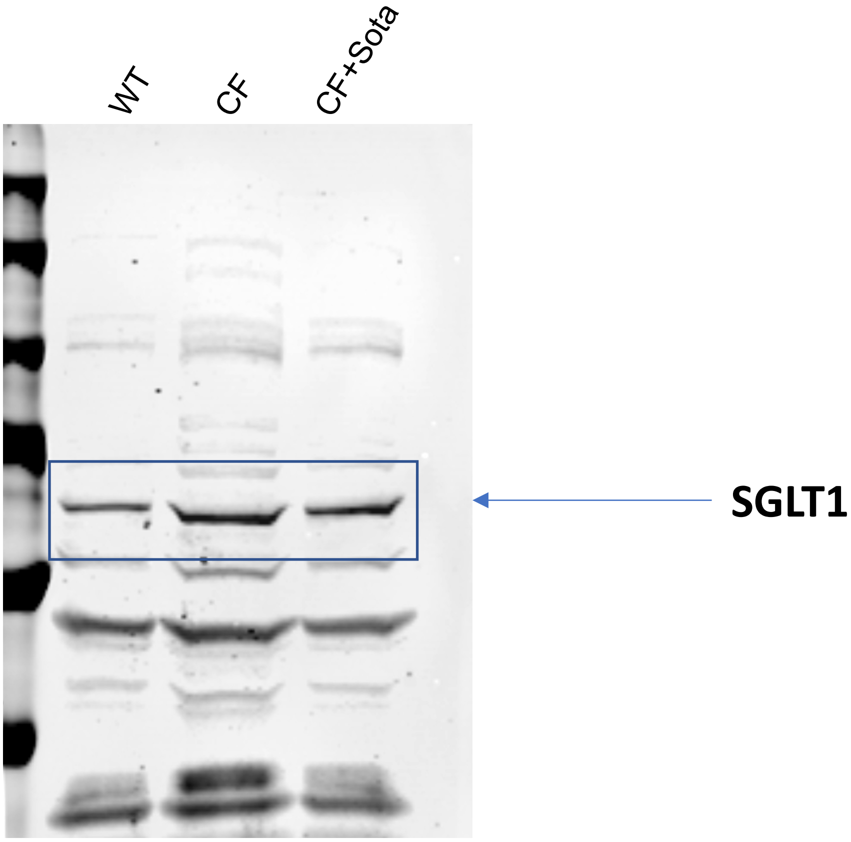

Full unedited gel for Supplementary Figure 11B

GRP78

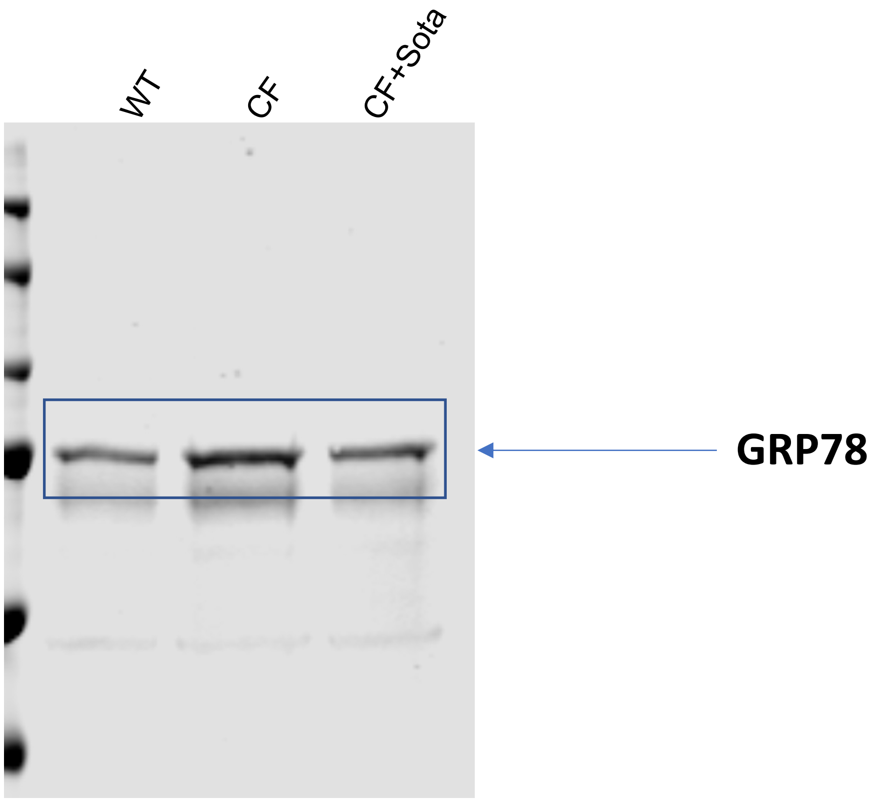

Full unedited gel for Supplementary Figure 11B

p-IRE1α

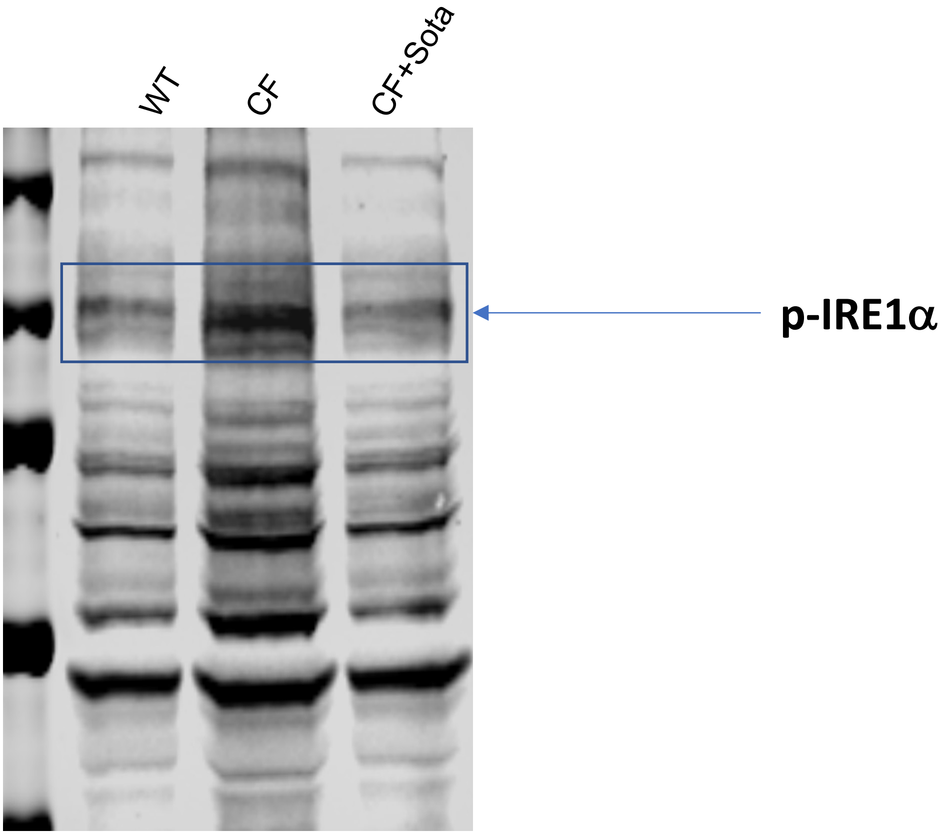

Full unedited gel for Supplementary Figure 11B

IRE1α

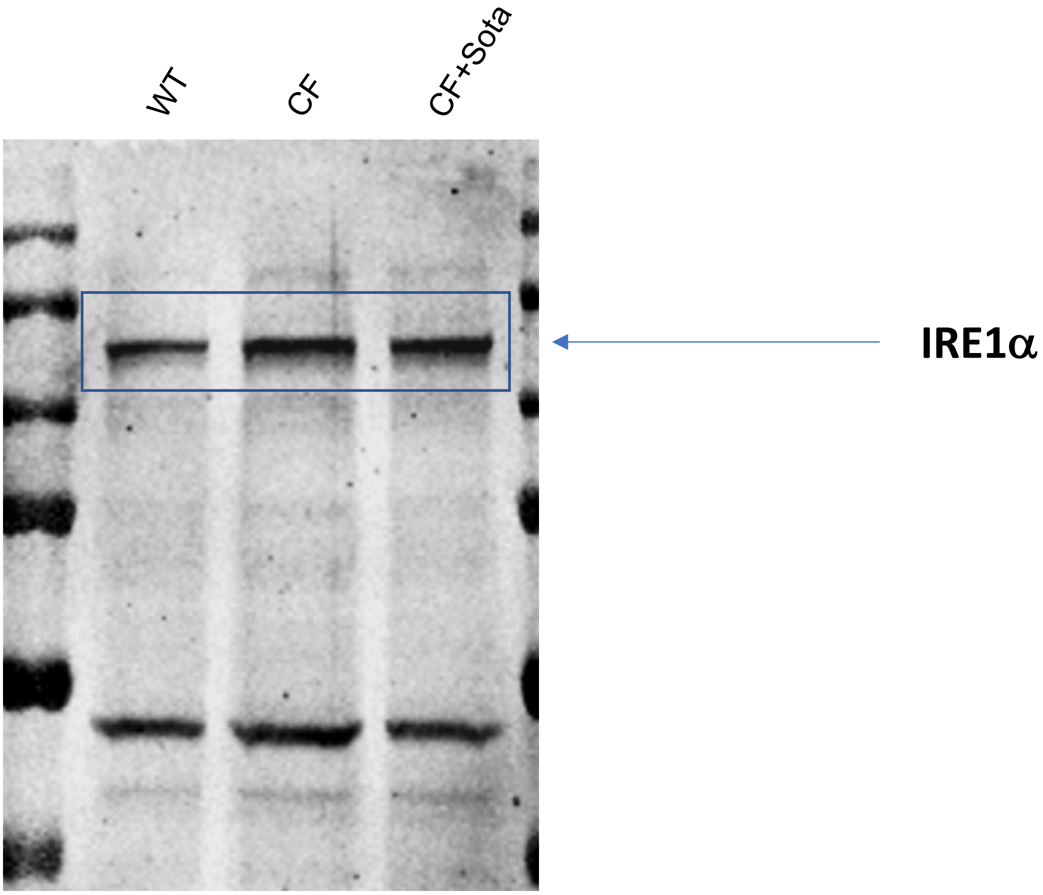

Full unedited gel for Supplementary Figure 11B

XBP1s

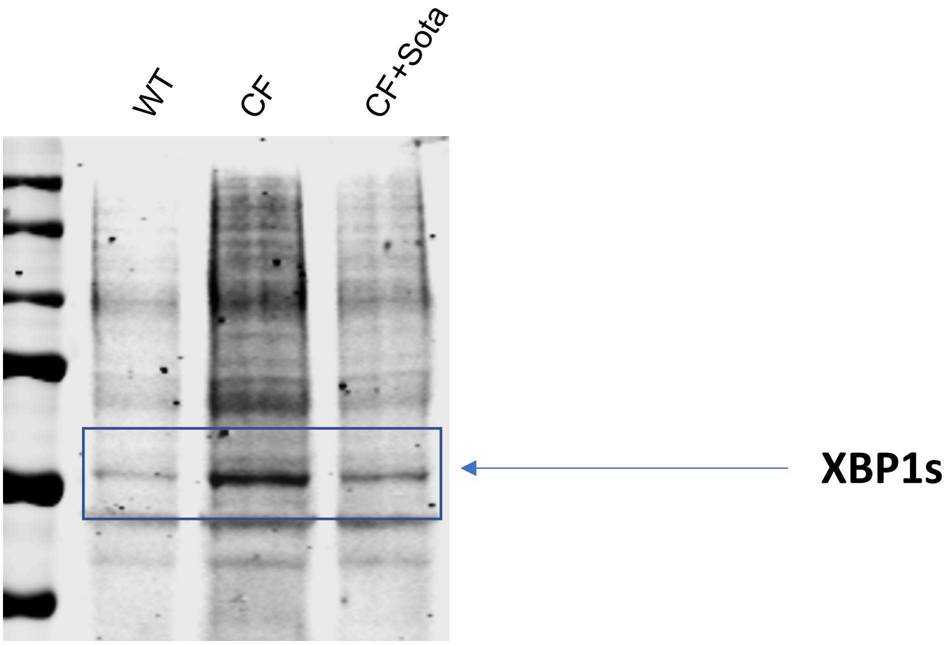

Full unedited gel for Supplementary Figure 11B

β-actin

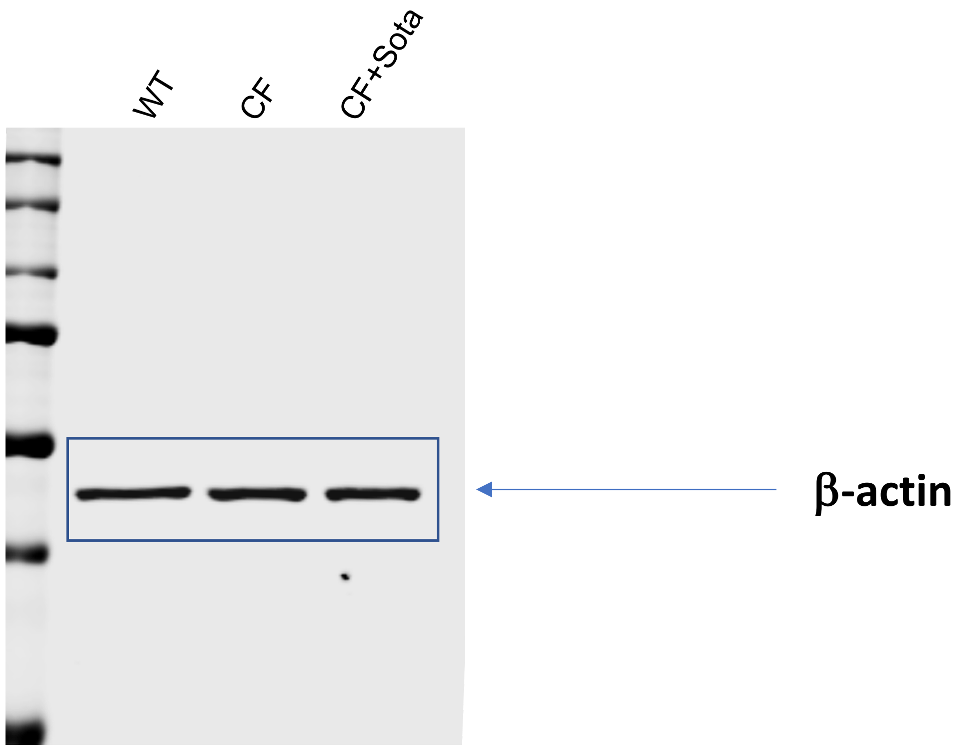

Supplement: Unedited blot and gel images [file jciinsight-9-165826-s034.pdf]
